# A deep generative model for deciphering cellular dynamics and in silico drug discovery in complex diseases

**DOI:** 10.1038/s41551-025-01423-7

**Published:** 2025-06-20

**Authors:** Yumin Zheng, Jonas C. Schupp, Taylor Adams, Geremy Clair, Aurelien Justet, Farida Ahangari, Xiting Yan, Paul Hansen, Marianne Carlon, Emanuela Cortesi, Marie Vermant, Robin Vos, Laurens J. De Sadeleer, Ivan O. Rosas, Ricardo Pineda, John Sembrat, Melanie Königshoff, John E. McDonough, Bart M. Vanaudenaerde, Wim A. Wuyts, Naftali Kaminski, Jun Ding

**Affiliations:** 1 Quantitative Life Sciences, Faculty of Medicine & Health Sciences, McGill University, Montreal, Quebec Canada; 2 Meakins-Christie Laboratories, Translational Research in Respiratory Diseases Program, Research Institute of the McGill University Health Centre, Montreal, Quebec Canada; 3 Pulmonary, Critical Care and Sleep Medicine, Yale University School of Medicine, New Haven, CT USA; 4 Department of Respiratory Medicine, Hannover Medical School, German Center for Lung Research (DZL), Hannover, Germany; 5 Biomedical Research in End-Stage and Obstructive Lung Disease (BREATH), Hannover Medical School (MHH), German Center for Lung Research (DZL), Hannover, Germany; 6 Biological Sciences Division, Pacific Northwest National Laboratory, Richland, WA USA; 7 Laboratory of Respiratory Diseases and Thoracic Surgery (BREATHE), Department of CHROMETA, KU Leuven, Leuven, Belgium; 8 Division of Pulmonary, Critical Care and Sleep Medicine, Baylor College of Medicine, Houston, TX USA; 9 Division of Pulmonary, Allergy, Critical Care and Sleep Medicine, Department of Medicine, University of Pittsburgh, Pittsburgh, PA USA; 10 Mila - Quebec AI Institute, Montreal, Quebec Canada

**Keywords:** Virtual drug screening, Biomedical engineering, Machine learning, Drug discovery, Computational models

## Abstract

Human diseases are characterized by intricate cellular dynamics. Single-cell transcriptomics provides critical insights, yet a persistent gap remains in computational tools for detailed disease progression analysis and targeted in silico drug interventions. Here we introduce UNAGI, a deep generative neural network tailored to analyse time-series single-cell transcriptomic data. This tool captures the complex cellular dynamics underlying disease progression, enhancing drug perturbation modelling and screening. When applied to a dataset from patients with idiopathic pulmonary fibrosis, UNAGI learns disease-informed cell embeddings that sharpen our understanding of disease progression, leading to the identification of potential therapeutic drug candidates. Validation using proteomics reveals the accuracy of UNAGI’s cellular dynamics analysis, and the use of the fibrotic cocktail-treated human precision-cut lung slices confirms UNAGI’s predictions that nifedipine, an antihypertensive drug, may have anti-fibrotic effects on human tissues. UNAGI’s versatility extends to other diseases, including COVID, demonstrating adaptability and confirming its broader applicability in decoding complex cellular dynamics beyond idiopathic pulmonary fibrosis, amplifying its use in the quest for therapeutic solutions across diverse pathological landscapes.

## Abstract (web summary)

UNAGI deciphers cellular dynamics from human disease time-series single-cell data and facilitates in silico drug perturbations to discover drugs potentially active against complex human diseases.

## Main

Complex diseases emerge through the interaction of genetic and environmental factors over time. The complexity of the interactions between these heterogeneous factors among individuals and populations challenges the understanding of disease progression^1–3^. Treating multifactorial diseases requires therapies that address multiple interacting processes, but most therapies are developed using animal or cell culture models that fail to capture the complexity and dynamics of human disease^4,5^. Novel approaches that capture disease dynamics and cellular complexity are needed to facilitate the discovery and implementation of efficient therapeutic interventions for complex diseases.

Methods based on clinical data and electronic health records such as Boolean networks^6^, Bayesian networks, support vector machines^7^ and decision trees^8^ can chart disease continuum states^9^, but do not address the molecular, cellular and genetic mechanisms underlying disease progression. This limitation lies in the lack of high-resolution genomic profiling^10^, which is crucial for understanding gene markers and gene networks, as well as for identifying therapeutics. Single-cell RNA sequencing (scRNA-seq) stands at the frontier of potential solutions, offering an opportunity to analyse cell populations at single-cell resolution^11,12^. This technology can profile complex and heterogeneous biological systems^13,14^, uncovering rare cell populations and aberrant cell states that are pivotal to diseases^15^. Computational methods^16–24^ such as Seurat, SCANPY, scVI, GraphSCC, scGNN and scGGAN analyse the noisy, high-dimensional and large-scale scRNA-seq data and can even sketch cellular dynamics. However, scRNA-seq data is often a snapshot of the cellular states at a specific time point and cannot account for the dynamic changes in cellular phenotypes, responses or differentiation states during disease progression. When applied to time-series scRNA-seq data, these methods tend to perceive the data as discrete snapshots, overlooking the continuity and temporal progression inherent in time-series data. Computational methods have been developed to address the challenges raised by time-series single-cell transcriptome data. However, both conventional methods, such as scdiff^25^ and CSHMMs^26,27^, and deep-learning-based methods, such as RVAgene^28^ and TDL^29^, are engineered for generic single-cell data processing, inadvertently bypassing the specialized necessities tied to complex diseases. The preprocessing and normalization, often required by noisy single-cell data for complex diseases, can shift the data into unconventional distributions, making them ill-suited for the direct application of many existing models^19,30^. In addition, the absence of disease-specific optimization in these approaches limits their understanding of the disease. When it comes to the step of cell embedding learning, existing methods are devoid of the flexibility to integrate disease-specific signatures. This limitation makes them less effective at capturing the nuanced biological variations associated with complex diseases. Finally, a salient gap in current single-cell methodologies is the absence of unsupervised in silico perturbation exploration capabilities. Although methods such as scGPT^31^, GEARS^32^ and scGen^33^ can perform in silico perturbations, they were not designed to process time-series data and often require the experimental screening of cellular response to genetic perturbation as supervision. Even if one were to adopt existing unsupervised generative models, such as scVI, for this particular purpose, their capacity to simulate interventions is hindered by inadequate incorporation of disease information. These existing unsupervised generative methods are often not disease specific, treating all genes in a similar manner across various diseases. Consequently, they often fail to identify critical genes associated with specific disease progression, which hold potential for therapeutics. Furthermore, existing approaches, whether supervised or unsupervised, are often generic and fail to deliver disease-informed in silico drug screening. This shortcoming arises from the lack of information exchange between cell embedding learning and gene regulatory network inference underlying disease progression. These methods usually cannot feedback the understanding of disease progression (for example, critical genes and regulators that modulate disease progression) to improve cellular representation (that is, emphasizing critical genes more than others), and vice versa. Consequently, there is an unmet need for unsupervised methods that can understand disease progression and adapt this comprehension to virtually examine thousands of potential drugs and compounds using single-cell disease data without relying on ground truth training data. The ever-increasing availability of large-scale public drug databases, such as the Connectivity Map (CMAP) database^34,35^, may provide the missing link to the unsupervised single-cell in silico drug perturbations. Coupled with this, given the vast pool of drug candidates and the intricate cellular dynamics of diseases, an interactive visualization tool is important for efficiently probing potential drugs and priming them for further experimental validation.

To bridge these gaps, here we introduce UNAGI, a comprehensive unsupervised in silico cellular dynamics and drug screening framework. UNAGI deciphers cellular dynamics from human disease time-series single-cell data and facilitates in silico drug perturbations to earmark therapeutic targets and drugs potentially active against complex human diseases. All outputs, from cellular dynamics to drug perturbations, are rendered in an interactive visual format within the UNAGI framework. Nestled within a deep-learning architecture variational autoencoder-generative adversarial network (VAE-GAN), UNAGI is tailored to manage diverse data distributions frequently arising post-normalization. It also uses disease-informed cell embeddings, harnessing crucial gene markers derived from the disease dataset. On achieving cell embeddings, UNAGI fabricates a graph that chronologically links cell clusters across disease grades (reflecting changing cellular states during disease progression and quantified using patient-derived samples or cells), subsequently deducing the gene regulatory network orchestrating these connections. UNAGI can leverage time-series data, enabling the characterization of cellular dynamics and capture of disease markers and gene regulators. Lastly, the deep generative nature of the UNAGI framework facilitates an in silico drug perturbation module, simulating drug impacts by manipulating the latent space informed by real drug perturbation data from the CMAP database. This allows for an empirical assessment of drug efficacy based on cellular shifts towards healthier states following drug treatment. The in silico perturbation module can similarly be used to investigate therapeutic pathways, using an approach akin to the one used in drug perturbation analysis.

We demonstrate UNAGI on a comprehensive single-nuclei RNA-seq (snRNA-seq) idiopathic pulmonary fibrosis (IPF) dataset. IPF is a complex lethal lung disease characterized by irreversible lung scarring, leading to progressive decline in lung function and death^36–38^. Present therapeutic options for IPF are markedly narrow; two Food and Drug Administration (FDA)-approved drugs, pirfenidone^39^ and nintedanib^40^, that slow lung function decline, but do not reverse fibrosis^41^. Despite their approval, their specific impact on disease progression mechanisms remains unclear^40–42^. Recent single-cell profiling studies^12,15^ highlighted the molecular and cellular diversity of the IPF lung, revealing extensive changes in lung-resident cells in IPF^43^. We apply UNAGI to the dataset containing single-nuclear sequencing of samples from differentially affected lung regions. This approach aims to better understand the changes that lung fibroblasts, key pathogenic cells in fibrosis, undergo as fibrosis progresses in the human lung and to potentially identify agents that may slow down or reverse these changes. This analysis demonstrates UNAGI’s ability to generate compact low-dimensional representations of the dynamic cellular transcriptomic shifts during disease progression outperforming existing methods. In addition, we conduct proteomics analysis of the same lungs, as well the ex vivo of human pulmonary fibrosis using precision-cut lung slices (PCLS)^44,45^, to experimentally confirm the results and predictions of UNAGI. Taken together, our findings corroborate UNAGI’s capability not only in decoding cellular dynamics and underpinning regulatory networks but also in potentially accelerating drug development by spotlighting potential therapeutic targets and drug candidates.

## Results

### Overview of UNAGI conceptual framework

UNAGI, a unified in silico cellular dynamics and drug screening framework, is a computational framework that integrates time-series single-cell sequencing data with deep-learning techniques to unravel cellular dynamics and identify therapeutic interventions against multifaceted diseases. This is achieved using the following four components.

**Fig. 1:**
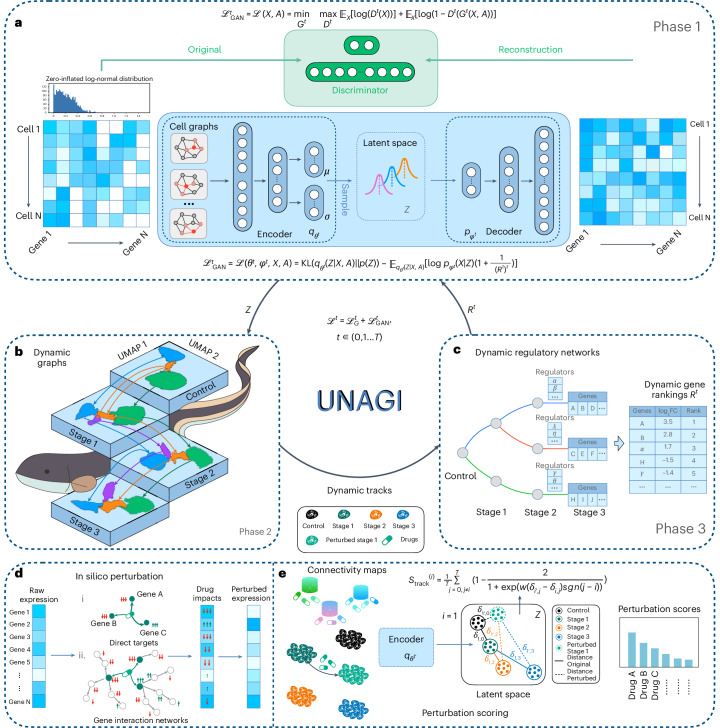
UNAGI overview. **a**, Phase 1: UNAGI uses a VAE-GAN paired with a graph convolution layer. This set-up harnesses the complexities of single-cell data, producing a ‘*Z*’ latent space that bridges encoding and decoding with minimal error. **b**, Phase 2: derived from the ‘*Z*’ embeddings, a temporal dynamics graph emerges. Here the Leiden clustering method discerns cell populations, subsequently UNAGI connects them across disease grades based on their inherent similarity. **c**, Phase 3: the iDREM tool comes into play, spotlighting key gene regulators and genes that influence disease progression. These insights are channelled into an iterative model training, honing in on specific gene markers of the disease. **d**, With the model in place, UNAGI initiates in silico perturbations, either directly tweaking drug target gene expressions (i) or manipulating gene expressions via established gene interaction networks (ii) to simulate drug treatment impact. **e**, UNAGI’s encoder processes the perturbed cell population alongside its peers. The perturbation scores, derived from the ‘*Z*’ space embeddings generated by the UNAGI encoder, assist in identifying potential drug candidates. These candidates are evaluated based on their ability to transition diseased cells towards healthier states, such as those resembling healthy control cells, thereby contributing to the treatment of the disease.

(1) UNAGI applies a VAE-GAN to capture cellular information in a reduced latent space (Fig. 1a). It processes single-cell data as continuous, zero-inflated log-normal (ZILN) distributions (or other distributions that well fit the data in other application scenarios) because this often better matches the distribution of single-cell data post rigorous preprocessing and normalization (for example, in the IPF data used in this study). With a cell-by-gene normalized counts matrix as input, a cell graph convolution (GCN) layer is introduced to manage the sparse and noisy nature of the data. In particular, the GCN layer leverages the structured relationships between cells to mitigate the dropout noise common in single-cell data, enhancing the accuracy of cellular representations. This data, further refined by a VAE, results in lower-dimensional embeddings, with an adversarial discriminator ensuring the synthetic quality of these representations. (2) After embedding, cell populations are identified using the Leiden clustering approach and visualized with UMAP. A temporal dynamics graph spanning disease grades is then constructed by evaluating cell population similarities during the disease progression, linking them based on their likeness (Fig. 1b). Each trajectory within the graph then forms the basis for deriving gene regulatory networks using the iDREM tool^46^ (Fig. 1c). (3) An iterative refinement process toggles between the embedding and temporal cellular dynamics. During the embedding phase, disease-associated genes and regulators (such as transcription factors, cofactors and epigenetic modulators) identified from the reconstructed temporal cellular dynamics are emphasized. This ensures that cell representation learning consistently prioritizes these key elements related to disease progression in every iteration. (4) Upon reaching predefined stopping criteria, UNAGI then uses in silico perturbations to quantify the effectiveness of therapeutic interventions (Fig. 1d). Using the trained VAE-GAN generative model, UNAGI simulates cells under various drug treatments or pathway perturbations. Each perturbation’s impact is scored and ranked based on its ability to shift the diseased cells closer to a healthier cellular state (Fig. 1e). The detailed model architecture and training parameters can be found in Supplementary Note 1.

### Binning IPF samples into tissue fibrosis grades based on the alveolar surface density

**Extended Data Fig. 1:**
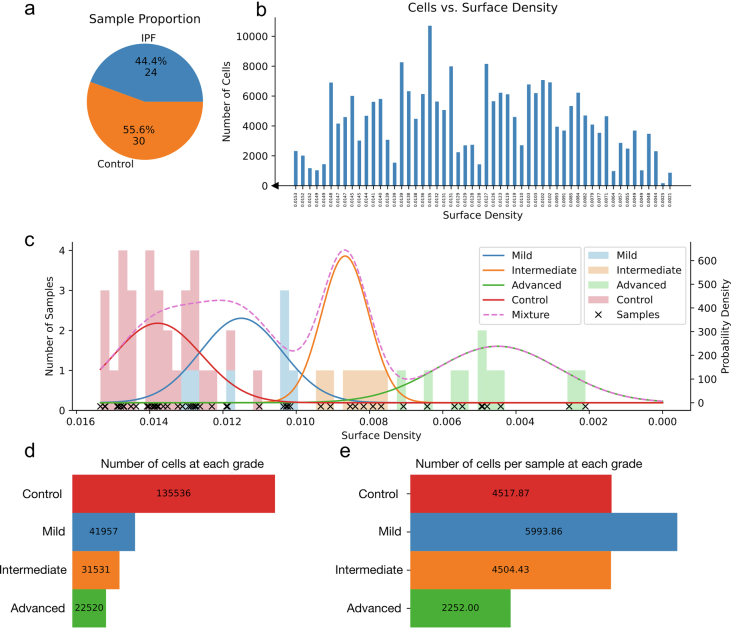
Statistics of the IPF dataset and tissue fibrosis grades binning based on surface density. **a**, A pie chart illustrating the distribution of Control (healthy) and IPF samples in the dataset. **b**, A bar chart showing the number of cells in each sample, ordered by their surface density, indicating the grade of tissue involvement in fibrosis (from Control to Advanced). **c**, A histogram representing the surface densities of the samples, categorized by grades as determined by a Gaussian density estimator. The plot includes curves representing the probability density of each Gaussian component and the overall multi-Gaussian distribution. **d**, A bar chart displaying the number of cells at each tissue fibrosis grade. **e**, A bar chart illustrating the number of cells per sample at each tissue fibrosis grade.

**Extended Data Fig. 2:**
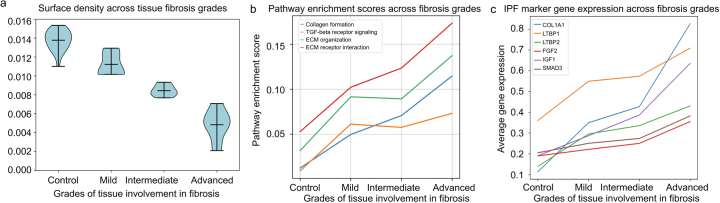
IPF tissue involvement is marked by surface density and fibrotic marker. **a**, Violin plots representing the alveolar surface density (of samples across different tissue fibrosis grades, with the mean surface density indicated by the red lines. **b**, Line plot of pathway enrichment scores across different tissue fibrosis grades **c**, Line plots of the gene expression of the fibrotic markers across different tissue fibrosis grades.

A true longitudinal profiling of the lung cells from the same patient with different grades of tissue involvement in fibrosis is impossible because patients are never biopsied more than once. Cells or micro-dissected regions from the same clinical stage can vary substantially in their actual cellular states and grades of tissue involvement in fibrosis^47^. Thus, to investigate the cellular dynamics along the progression of human IPF tissues, we used a widely adopted validated strategy that analyses samples from variably affected regions of the IPF lung to assess histological fibrosis progression^47–55^. The justification for this strategy is that IPF does not progress randomly; rather, it stereotypically advances from the lung periphery to the centre, and from the lower lung zones to the upper lung zones^56^. This approach has been validated extensively^49–53,55,57^. Therefore, cells from differentially affected regions could be assumed to represent different fibrosis grades in disease progression. To build the surrogate ‘longitudinal’ single-cell data, here we used a Gaussian density estimator (Supplementary Note 2) to classify all samples (and thus all cells) into different grades of tissue involvement in fibrosis (tissue fibrosis grades), measured by the alveolar surface density, a previously validated measure of lung fibrosis^47,50,53^ (Extended Data Fig. 1a,b). The model learns the best number of tissue-fibrosis-grade bins in the IPF tissue and the associated Gaussian parameters (mean and standard deviation) for each bin. We analysed a total of 54 lung region samples from 19 patients, binning them into 4 tissue fibrosis grades based on the extent of tissue fibrosis as reflected by surface density—none (control), mild, intermediate and advanced—based on the surface density. The fibrosis-related pathway enrichment scores and the expression changes of fibrotic markers such as *COL1A1*^58^, *LTBP1*^59^, *LTBP2*^60^, *FGF2*^61^, *IGF1*^62^ and *SMAD3*^63^ (Extended Data Fig. 2) show a clear trend of increasing tissue fibrosis grades in IPF. This four-tissue fibrosis grade binning has been previously validated^47,49,50,52^. Following the density estimation analysis, we assigned samples and cells to these four tissue fibrosis grades (Extended Data Fig. 1c). Specifically, 30 samples from 10 patients were categorized as none/control (135,509 cells). Seven samples from 5 patients were classified as mild (41,949 cells). Intermediate included 7 samples (31,512 cells) from 5 patients, while advanced comprised 10 samples (22,507 cells) from 6 patients (Extended Data Fig. 1d). As shown in Extended Data Fig. 1e, there is a discernible increase in stromal cells starting from mild, hinting at a possible rise in fibroblasts from this tissue fibrosis grade onwards.

### UNAGI identifies varying stromal cell populations across IPF progression

**Fig. 2:**
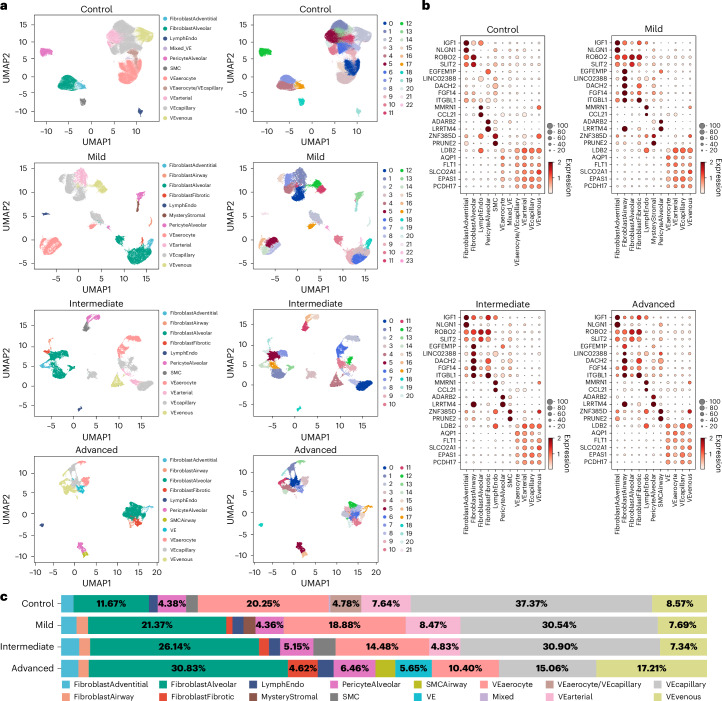
UNAGI identifies progressive heterogeneous cell populations across tissue fibrosis grades in IPF. **a**, UMAP visualization: stromal cells across various tissue fibrosis grades in IPF are depicted. Each point corresponds to a cell; the first column categorizes them by cell type (for example, SMC, smooth muscle cell; VE, vascular endothelial), and the second by Leiden cluster IDs. **b**, Gene dot plots: dot plots illustrate the key biomarkers for each identified cell type across four grades of fibrosis involvement in IPF. In these plots, the size of each circle indicates the proportion of cells expressing the gene, and the circle’s colour reflects the level of normalized gene expression. **c**, Cell composition chart: a visualization of the shifts in cell-type composition along with IPF disease progression. Colours indicate the specific cell type. Notably, there is a discernible expansion of fibroblast cells as the disease progresses.

After applying UNAGI to the IPF snRNA-seq dataset and performing clustering and visualization on the latent space, we explored the shifts and changes in stromal cell populations using UNAGI. The average adjusted Rand index (ARI) and normalized mutual information (NMI) were both 0.74 for all tissue fibrosis grades. UNAGI identified 11 distinct cell types in controls, with more emerging in subsequent tissue fibrosis grades (Fig. 2a), which we annotated based on the expression of canonical cell markers (Fig. 2b and independent manual cell-type annotations in Supplementary Fig. 1). UNAGI can capture cell subpopulations, such as fibrotic fibroblasts and airway fibroblast cells, suggesting extended fibrosis through the progression. UNAGI uncovered differences in cellular heterogeneity: smooth muscle cells (SMC; marked by *ZNF385D* and *PRUNE2*) and alveolar pericyte cells (characterized by *ADARB2* and *LRRTM4*) were predominantly homogeneous. By contrast, fibroblast cell populations showed greater heterogeneity, within both alveolar (denoted by *ROBO2* and *SLIT2*^64^) and adventitial fibroblasts. Fibroblast proportions largely increase in IPF compared with controls—from less than 15% to more than 40%—validating that fibroblast accumulation is a hallmark of IPF progression^65^ (Fig. 2c). The alveolar fibroblast cell population exhibits the most substantial increase, while the fibrotic fibroblast archetype appeared only in subsequent tissue fibrosis grades. The proportion of vascular endothelial cells consistently decreases as IPF progresses. The cell embeddings from IPF data reveal progressive shifts in cell populations across tissue fibrosis grades in IPF, which serve as a foundation for constructing a temporal dynamic graph depicting disease progression.

### UNAGI reconstructs temporal dynamics and gene regulatory networks in disease progression

**Fig. 3:**
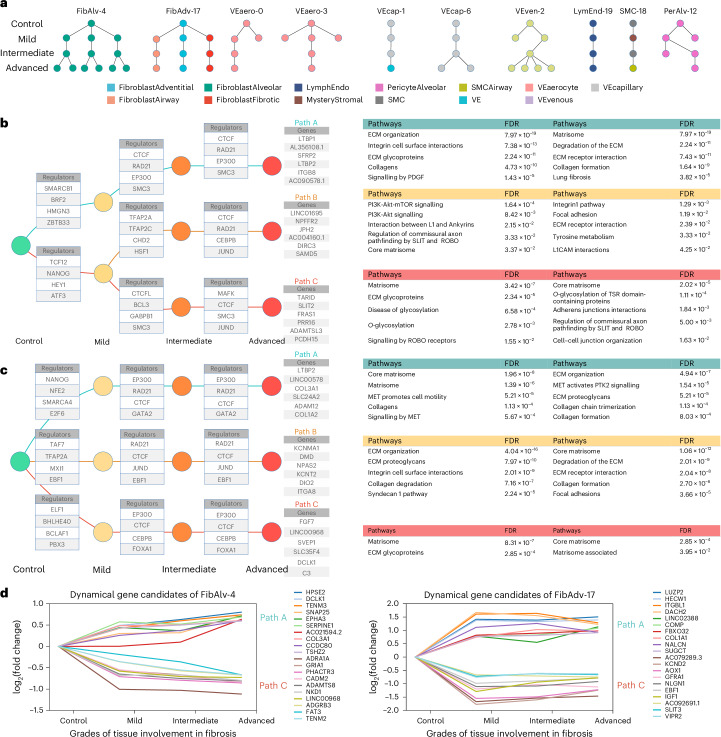
UNAGI reconstructs the temporal dynamics and the underlying gene regulatory networks of cellular dynamics during IPF progression. **a**, Dynamics graph of IPF progression within the stromal cell lineage, comprising four tissue fibrosis grades. Each node symbolizes a cell population, coloured according to cell type, and the edges between two nodes depict the progression trajectory across tissue fibrosis grades. Tracks (trajectories), spanning from control to advanced, are named with the specific cell type and the corresponding control cluster ID. **b**, Gene regulatory networks for the FibAlv-4 track were reconstructed using the iDREM tool. Individual nodes signify a set of genes, and edges connecting two nodes represent gene regulators regulating expression changes. Paths encompassing nodes from control to advanced depict a consistent set of genes showing the same expression changes throughout IPF progression. The enriched pathways associated with gene paths were also provided. **c**, The temporal regulatory networks for the FibAdv-17 track. **d**, Line chart of expression of the top dynamic gene candidates on the FibAlv-4 and FibAdv-17 tracks, the top 10 most increasing and the top 10 most decreasing candidate marker genes through the IPF progression. We applied FDR correction in **b** using the Benjamini–Hochberg (BH) procedure.

UNAGI reconstructs the cellular dynamics associated with time-series or disease progression data based on the cell embeddings learned by the model. Within our analytical framework, a ‘track’ delineates a distinct trajectory within the reconstructed dynamics graph, marking the sequential cellular state transitions corresponding to specific cell clusters or populations. These tracks not only identify pathways but also chronicle the journey of cellular progression and evolution. Within stromal cells, we have discerned ten distinct progression tracks (Fig. 3a), transitioning from the control to advanced tissue fibrosis grade. Because of the established role of fibroblasts in pulmonary fibrosis^58,66,67^, we focused on two tracks that delineate fibroblast progression in human IPF. FibAlv-4 traces the cellular state shifts of alveolar fibroblast cells during IPF progression, while FibAdv-17 illustrates the cellular dynamics of adventitial, airway and fibrotic fibroblasts. Of note, the fibroblast tracks in the dynamics graph contain multiple branches, potentially reflecting the multifaceted roles of fibroblast cells in fibrosis^68^.

The gene regulatory network of FibAlv-4, as reconstructed by UNAGI, highlights the central role of gene regulators CTCF, RAD21, SMC3 and especially fibrosis-promoting EP300^69,70^. This is further supported by the genes in path A of the FibAlv-4 track, which include recognized fibrosis biomarkers such as *LTBP1* and *LTBP2*^60,71^ (Fig. 3b). Pathways enriched in track FibAlv-4 include the following: in path A, collagen and extracellular matrix (ECM) pathways^72^; in path B, the PI3K-Akt-mTOR signalling pathway and the focal adhesion pathway (both are important in lung fibrosis)^73–75^ (Fig. 3b); and in path C, SLIT/Robo signalling pathway, less studied but with a potential role in regulation of fibrosis^64,76^. UNAGI also uncovered pathways that are implicated in fibrosis but have not been firmly established as contributors to IPF development, such as NCAM1 interactions^77,78^.

The FibAdv-17 track highlights the contribution of adventitial fibroblasts to matrix remodelling. Enriched pathways encompass general ECM-related pathways, including the ones of collagen formation, organization, trimerization and degradation, with some variation between paths A and C (Fig. 3c). The MET-activated PTK2 signalling pathway^79^, a substantial player in pulmonary fibrosis progression, is also highlighted. The genes in path B, including *KCNMA1*^80^, *NPAS2*^81^, *ITGA8*^82^ and *DIO2*^83^, have all been associated with IPF. The depth and precision of the reconstructed gene regulatory network are underscored by its ability to pinpoint not only pivotal gene regulators and pathways but also the target genes that they regulate. These target genes, especially those that exhibit differential expression across tissue fibrosis grades, provide insights into the temporal dynamics of IPF progression. In the context of the FibAlv-4 track, the method identifies both *COL3A1* and *SERPINE1*, which are induced by the transforming growth factor-β (TGFβ) pathway^84^ and are hallmarks of the IPF lung^85^. Moreover, it identifies less-studied fibrotic marker candidates such as *DCLK1*, *TENM3*, *TENM2*, *ADRA1A* and *GRIA1*, which have also been implicated in pulmonary fibrosis^86–89^ (Fig. 3d).

Taken together, UNAGI’s full-spectrum discovery of well-established as well as less-known, but still associated, gene regulators, pathways and their target genes underscores the method’s robustness in unravelling the intricate molecular interplay underlying the IPF progression.

### UNAGI discovers dynamical and hierarchical static markers across disease grades

Conventional single-cell analysis primarily identifies differentially expressed markers between healthy and diseased cells. By contrast, we developed UNAGI to identify dynamic marker genes that offer a longitudinal profile of cellular state changes throughout IPF progression. It discerns dynamic markers for individual cell populations, tracing gene expression shifts across disease grades. All identified candidate biomarker genes from the temporal gene regulatory network for each track are subjected to a permutation test to assess their statistical significance. This test involves randomly shuffling cells from the track across various grades to establish a background distribution for comparative analysis. Candidate genes that are deemed statistically significant through this test are considered as dynamic markers, closely associated with the track in the analysis (as detailed in the ‘Dynamic and hierarchical static markers discovery’ section of Methods).

**Fig. 4:**
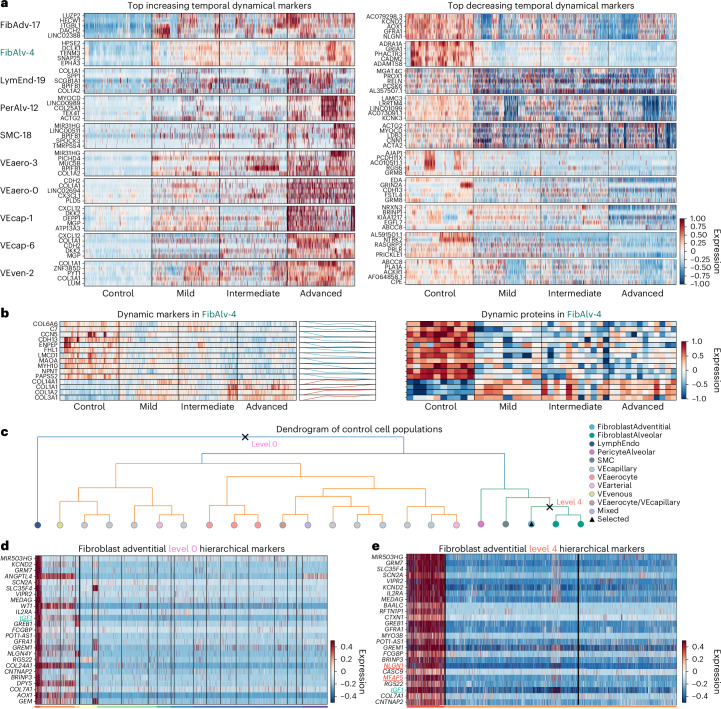
UNAGI comprehensively discovers dynamical and hierarchical static markers across various tissue fibrosis grades in IPF. **a**, Heat maps of top increasing and decreasing temporal dynamic markers, *z*-score normalized. **b**, Heat maps of dynamic gene markers (left) and protein expressions (right) from the FibAlv-4 cluster, with line plots showing gene expression shifts during IPF progression. **c**, Dendrogram visualizing control cell populations. Each node signifies a cell-type-specific population. The fibroblast adventitial cluster is accentuated. Using UNAGI, various hierarchical biomarkers are discernible at different levels, either contrasting with other cell types or juxtaposing subpopulations within the same cell type. **d**, Heat map of top 25 hierarchical static markers in the fibroblast adventitial cluster at level 0, showing general cell-type markers. **e**, Heat map of top 25 hierarchical markers in the fibroblast adventitial cluster at level 4, compared with two fibroblast alveolar clusters, showing cell subtype markers.

Figure 4a shows heat maps of the top 5 dynamic markers for each track, both those that increase and decrease during disease progression (a comprehensive list is available in Supplementary Table 1). For instance, in the FibAdv-17 track, markers such as *LUZP2*, *ITGBL1* and *AOX1*, previously reported as differentially expressed in IPF^90^, are highlighted. Notably, *NLGN1*, *GFRA1* and *AOX1* are markers for adventitial fibroblasts^11^ and emerge as a top-decreasing temporal dynamic marker in this track, suggestive of a loss of respective cell identity. The FibAlv-4 track, however, features markers such as *DCLK1*, *TENM3*, *ADRA1A*, *GRIA1* and *EPHA3*, all of which have strong ties to lung fibrosis^86–89,91^. Some of them are also differentially expressed in all cells during disease progression (Supplementary Fig. 2). It is important to mention that while our discussion primarily focused on monotonically increasing and decreasing biomarkers, which are of main interest in our study, our model can also identify biomarker genes with other patterns. An example of this is genes that initially increase and then decrease, as observed in path B of the FibAdv-17 track.

A common limitation of single-cell transcriptomic data is that it only reflects transcript levels. To validate the markers discovered by UNAGI, we used proteomics data, demonstrating gene–protein overlaps and corroborating our transcript-level findings. We performed proteomics of 30 matched tissue blocks from 10 IPF samples, with 3 samples each across different tissue fibrosis grades (based on the same surface density criteria), and 10 control donors, with 1 sample each (Supplementary Table 2). We identified 886 dynamic proteins, with 120 overlapping with our single-cell data (out of 2,484 genes). This overlap is significant and much higher than expected by chance (chi-square test *P* value = 9.354 × 10^−18^). There are 40 out of 120 dynamic markers that overlap with dynamic proteins. Hypergeometric testing on individual tracks revealed statistical significance for protein-coding genes of dynamic proteins in four specific tracks (Supplementary Fig. 3).

A reassuring observation from our snRNA-seq and proteomics data was again the combination of the identification of well-known and validated molecules, with molecules that have been implicated but not deeply studied in fibrosis. The FibAlv-4 track notably contained 137 dynamic protein-encoding genes, with 14 of these genes producing dynamic proteins (Fig. 4b). Among these overlapping dynamic markers, five relate to collagens (COL1A1, COL1A2, COL3A1, COL6A6 and COL14A1), confirming that progressive matrix remodelling is intrinsically linked to the development of fibrosis^92^. Besides, many other overlapping dynamic markers have been previously associated with pulmonary fibrosis in computational analysis of bulk RNA-seq^93^ or mechanistic studies^94–96^. Beyond these well-established IPF markers, UNAGI also uncovers markers such as ROBO1, ROBO2^64^ and GLI2^97^, which have not been firmly linked to IPF but warrant further investigation.

UNAGI can identify both dynamic and static markers. While dynamic markers offer insights into cellular state changes throughout disease progression, static markers are crucial for distinguishing between different cell types and subpopulations within a given tissue fibrosis grade. Existing static biomarker discovery pipelines^16,17^ usually use a ‘one versus the rest’ strategy and may fail to distinguish the difference between different subtypes.

UNAGI explores the hierarchies of marker genes that not only distinguish different cell populations but also capture the heterogeneity among cell subpopulations. For instance, focusing on the FibAdv-17 cluster of controls, cell subpopulations are primarily divided into three main groups: fibroblasts, vascular endothelial cells and lymphatic endothelial cells (Fig. 4c and dendrograms of all four tissue fibrosis grades are in Supplementary Fig. 4). The fibroblast adventitial population spans five levels in the dendrogram. Figure 4d shows the top 25 positive hierarchical static markers for fibroblast adventitial cells at dendrogram level 0. These markers distinguish the fibroblast adventitial cluster from all other clusters. UNAGI’s results are consistent with the dendrogram structure, indicating the close relationship between fibroblast adventitial and fibroblast alveolar clusters. Notably, UNAGI identified key markers such as *IGF1* and collagen-encoded genes such as *COL24A1* and *COL7A1*, emphasizing the role of elevated interstitial collagen levels in IPF^98^. Other markers such as *ANGPTL4*^99^ and *WT1* further demonstrate the method’s precision in identifying relevant genes^100^ (top 25 level 0 positive and negative markers are detailed in Supplementary Fig. 5).

Figure 4e presents the top 25 positive hierarchical static markers for the fibroblast adventitial cluster at level 4 (subtype level). While there are some markers overlapped with level 0 markers, level 4 introduces unique markers potentially for subtypes such as *NLGN1* and MFAP5, and they are cell-type markers for adventitial fibroblasts^11,101,102^ (top 25 level 4 positive and negative markers are detailed in Supplementary Fig. 6). UNAGI’s ability to identify both temporal dynamic markers and hierarchical static markers offers a dual approach for detailed profiling of the disease from both intra-disease grade and longitudinal (inter-disease grade) perspectives, enhancing our understanding of its complexities.

### UNAGI identifies potential therapeutic pathways for IPF treatments

In the preceding sections, we described how UNAGI enhances our comprehension of biomarkers and cellular dynamics in the progression of IPF. Building upon this foundational understanding, we now shift our focus to the therapeutic frontiers opened by UNAGI. This involves leveraging its in silico perturbation capabilities, which are rooted in diseased-informed cell embeddings and the temporal dynamics of the disease. This approach facilitates the identification of potential therapeutic targets and pathways, which may contribute to advancements in IPF treatment. Detailed results of these pathway perturbations are systematically presented in Supplementary Table 3.

**Fig. 5:**
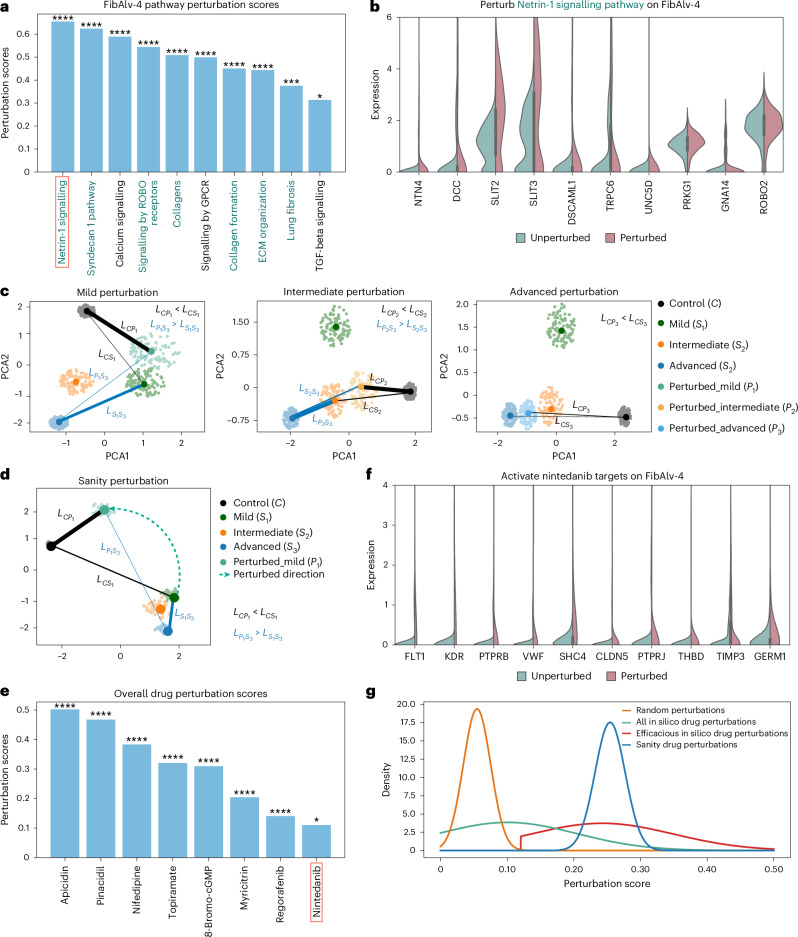
UNAGI identifies potential therapeutic pathways and potent drugs for IPF treatments. **a**, Bar chart of the track FibAlv-4 pathway perturbation results. The highlighted pathways are also identified in the reconstructed gene regulatory network of the track. **b**, Split-violin plot of the gene expression differences for the top 10 most changing genes of in silico Netrin-1 signalling pathway perturbation in mild of the FibAlv-4 track. **c**, PCA plots of latent space *Z* of in silico Netrin-1 signalling pathway perturbation effects and dots represent cells from distinct tissue fibrosis grades. Lines connected to two nodes are the PAGA connectivity score between two clusters, where the width of a line is proportional to the strength of the score, and the length of the line can represent the distance between the UNAGI embeddings of the two connected clusters (for example, line connecting control and perturbed mild (${L}_{C{P}_{1}}$)). **d**, PCA representation highlighting the impact of sanitary perturbation, which involves reversing the gene expression at mild back to the patterns observed in the control. **e**, Bar chart of the top overall drug perturbation results. **f**, Split-violin plot of gene expressions for the top 10 changing targets of nintedanib in the gene interaction network both before and after perturbation in mild of the FibAlv-4 track. **g**, Distribution patterns for various drug/compound perturbations. The *x*-axis represents the perturbation score, while the *y*-axis portrays the density of the fitted Gaussian distribution for each specific setting. We applied FDR correction in **a** and **e** using the BH procedure. Asterisks denote statistical significance as follows: *0.01 < FDR < 0.05; ***1 × 10^−^^4^ < FDR < 1 × 10^−3^; ****FDR < 1 × 10^−4^.

UNAGI provides a full spectrum of pathway perturbation results, ranging from well-established pathways to unexplored ones. Many of the top pathways predicted by UNAGI (Fig. 5a) align with known IPF-centric pathways, including pathways associated with TGFβ^84,103–105^ and collagen formation^98,105^. Among the top 10 identified therapeutic pathways, UNAGI identifies pathways whose role in IPF is relatively less studied such as the Netrin-1 signalling pathway (score = 0.6548, false discovery rate (FDR) = 3.4698 × 10^−19^), which is indicated to be mechanistically important in pulmonary fibrosis^87,106^; signalling by ROBO receptors (score = 0.5890, FDR = 1.1028 × 10^−14^)^64,107^; and GPCR signalling pathways, which are associated with G proteins, known to promote fibrosis, and have also generated interest as targets for IPF interventions^108^. Other less-studied pathways in IPF such as the calcium signalling pathway may hold important promise in fibrosis^109^. UNAGI also predicts unexplored pathways in IPF, including ion homeostasis and the inactivation of CDC42 and RAC1. Although these pathways were not previously linked to IPF, they may play a substantial role in IPF progression. For instance, CDC42 and RAC1, as members of the Rho family of small GTPases, are involved in fibroblast activation, suggesting that inhibiting these pathways could help mitigate fibrosis^110,111^.

Figure 5b shows the gene expression of the target genes of Netrin-1 pathways after perturbation. As can be seen in Fig. 5c, in silico pathway perturbations shift cellular states towards healthier conditions. Perturbed cell embeddings generated by the graph VAE-GAN model are visualized in a principal component analysis (PCA) plot, showing the effects of repressing the Netrin-1 signalling pathway across tissue fibrosis grades. In the mild fibrosis perturbation, the perturbed cell population (*P*_1_) is closer to the control (*C*) than to the mild cells (*S*_1_) and more distant from advanced cells (*S*_3_). The similarity in the embedding space is indicated by the thickness and length of connection lines, with a thicker and shorter line (${L}_{C{P}_{1}}$) representing higher similarity between control (*C*) and *P*_1_. Overall, shifting the pathway gene expression to control drives perturbed cellular states closer to controls and away from progressive tissue fibrosis grades (Fig. 5d). These results visually demonstrate the ability of UNAGI to simulate and potentially predict whether a specific pathway of gene set perturbations can improve cellular health—or reduce fibrosis.

### UNAGI screens potential drug candidates for IPF treatments

UNAGI’s in silico drug perturbation approach, akin to its pathway perturbation, leverages and integrates the CMAP dataset. Comprehensive results of all drug perturbations are detailed in Supplementary Table 4. UNAGI also offers a full spectrum of drug candidate predictions, from known IPF treatments to compounds with unexplored potential. UNAGI’s unsupervised in silico perturbation identified nintedanib (score = 0.1102, FDR = 0.0111), which is an FDA-approved drug for IPF, and ifenprodil^112^ (score = 0.2441, FDR = 2.275 × 10^−20^), an FDA orphan drug for IPF that has completed phase 2 trials (clinicalTrials.gov ID NCT04318704). These alignments with known treatments confirm UNAGI’s ability in identifying clinically relevant compounds. Some top predicted drug candidates that are not yet linked to IPF but have potential for further investigation are shown in Fig. 5e and are highlighted below.

Apicidin, with a score of 0.5021 and an FDR = 4.551 × 10^−105^), is a histone deacetylase (HDAC) inhibitor used in preclinical research. Previous studies have suggested that HDACs may be beneficial in pulmonary fibrosis, but their study has not progressed beyond the preclinical stage potentially because of safety concerns^113,114^. Another similar HDAC inhibitor, belinostat, was also picked up by UNAGI specifically, with no mention with regard to IPF so far. Nifedipine, scoring 0.3834 with an FDR = 1.152 × 10^−57^, is a calcium channel blocker widely used with a good safety profile. Despite some early encouraging results suggesting that calcium signalling inhibition in murine fibroblasts may be anti-fibrotic^115^, nifedipine has not been studied in humans. Cilomilast, a phosphodiesterase 4 (PDE4) inhibitor, has a score of 0.3082 and an FDR = 4.407 × 10^−35^. It has demonstrated potential in attenuating pulmonary fibrosis in mice^116^. Niguldipine, scoring 0.3842 and an FDR = 6.160 × 10^−58^, is a calcium channel blocker and an α1-adrenergic receptor antagonist, showing anti-fibrotic effects in the lung^115^. The compound 8-bromo-cGMP, which impacts *PRKG1*, has a score of 0.3099 and an FDR = 1.562 × 10^−35^, and is associated with the TGFβ pathways in the fibrosis process^117^. Other drugs, including ibudilast (score = 0.3053, FDR = 2.465 × 10^−34^) and topiramate (score = 0.3203, FDR = 2.411 × 10^−38^), have been identified, with the former potentially having anti-fibrotic effects similar to other PDE4 inhibitors^118^, and the latter regulating *GRIA1*, which is associated with lung fibrotic diseases^86,119^. Of note, a similar selective PDE4B inhibitor, nerandomilast, is currently evaluated in a phase 3 trial in patients with IPF (clinicalTrials.gov ID NCT05321069). Myricitrin (score = 0.2045, FDR = 2.590 × 10^−13^) has been shown to exhibit anti-fibrotic activity in certain conditions^120^, while regorafenib (score = 0.1407, FDR = 2.653 × 10^−5^) attenuates fibrosis by inhibiting the TGFβ pathway^121^. Furthermore, UNAGI also identified compounds with yet no established connection to IPF, such as eliprodil, an NMDA receptor antagonist^122^, worth further exploration.

The target gene intervention of nintedanib is shown in Fig. 5f. The corresponding perturbation results, visualized in Supplementary Fig. 7 across tissue fibrosis grades (mild–advanced), emphasize the potential of these drugs to shift cell populations towards healthier tissue fibrosis grades. The consistently higher PAGA connectivity scores between perturbed cell populations and healthier cellular tissue fibrosis grades indicate that the perturbed cell populations are more akin to healthier cells. Overall, UNAGI’s efficacious drug candidates (those that received significant FDR values) consistently surpass the therapeutic scores of random perturbations (Fig. 5g). These results were congruent with the outcomes from sanity drug perturbations (see Supplementary Note 3 for sanity drug perturbation method), during which we intentionally manipulated target gene expressions to the adjacent, healthier tissue fibrosis grades.

### Experimental validation of in silico drug perturbations via PCLS

To experimentally validate UNAGI predictions, we utilized a translational ex vivo fibrosis model—in which human PCLS are exposed to a fibrotic cocktail^123^. We tested the model predictions for nifedipine and nintedanib. PCLS were treated for 5 days with a control cocktail (CC) including all vehicles or a pro-fibrotic cocktail (FC) previously described^123,124^. Nifedipine and nintedanib of vehicle control treatment started on day 3 until day 5.

**Fig. 6:**
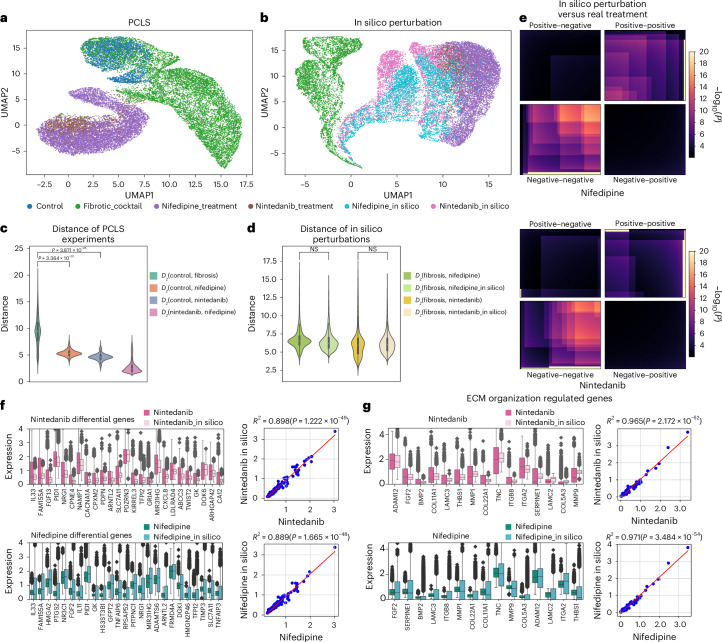
The predictions of UNAGI align with human PCLS drug validations. **a**, UMAP of PCLS data, each dot representing an individual cell. **b**, UMAP highlighting similarity between real and in silico treatments of nifedipine and nintedanib. **c**, Violin plots showing fibrotic cells shift towards healthy control cells after nintedanib and nifedipine treatments. For example, *D*_*z*_ (fibrosis, nifedipine) is the distance between fibrosis cells and fibrosis cells after nifedipine treatment. The number of cells *n* = 6,318, 9,346, 3,586 and 10,288 from left to right. The *P* value is calculated using one-sided Student’s *t*-test. **d**, Violin plots demonstrate strong alignment between in silico and real-world drug treatments. The number of cells *n* = 12,942, 9,836, 7,182 and 9,836 from left to right. The *P* value is calculated using the two-sided Kolmogorov–Smirnov test. NS, not statistically significant. **e**, The RRHO plots comparing gene expression changes in in silico perturbations and actual treatments for nifedipine and nintedanib, showing high similarity. **f**, Comparing the expression of the top 100 differential genes of real treatments (nintedanib or nifedipine versus fibrosis) to in silico perturbation results. The box plot visualizes the top 25 differential genes for each treatment. The top 100 differential genes are used to calculate the adjusted *R*^2^ metric and generate *R*^2^ plots. **g**, Comparing ECM organization target gene expressions from real treatments and in silico perturbations. The box plots visualize the top 15 differentially expressed ECM genes and use all ECM target genes to calculate the adjusted *R*^2^ metric. The number of cells *n* = 2,264, 4,918, 8,024 and 4,918 for nintedanib, nintedanib_in silico, nifedipine and nifedipine_ in silico in **f** and **g**. The *P* values of *R*^2^ (coefficient of determination) in **f** and **g** were calculated using the one-sided *F*-test. The boxes in **c**, **d**, **f** and **g** represent the interquartile ranges (IQRs), and the solid lines indicate the medians. The whiskers extend to points within 1.5 IQRs of the lower and upper quartiles.

As read-out, we performed snRNA-seq. When assessed based on experimental conditions, cells under both nifedipine and nintedanib treatments exhibit similar latent representations on the UMAP. This suggests their parallel roles in inhibiting fibroblast activation (Fig. 6a). Utilizing UNAGI’s perturbation module, nintedanib and nifedipine in silico perturbed cells gravitate towards the nintedanib-treated population, demonstrating potential therapeutic effects (Fig. 6b). Pairwise Euclidean distances between latent embeddings indicate that both treatments effectively steer the cellular state of fibrosis cells toward a healthier baseline (Fig. 6c) and the in silico treatments behave as real treatments (Fig. 6d). This observation is evidenced by the Mann–Whitney *U* test confirming the analogous anti-fibrotic properties of both treatments. The rank–rank hypergeometric overlap (RRHO) confirms that the markers identified in silico closely align with the biomarkers observed in the PCLS experiments (Fig. 6e). The adjusted *R*^2^ scores for nintedanib in silico (0.898, *P* = 1.222 × 10^−49^) and nifedipine in silico (0.889, *P* = 1.665 × 10^−48^) with respect to the top 100 differentially expressed genes (DEGs) in actual treatment versus fibrosis, as well as the top 25 markers in side-by-side comparisons (Fig. 6f; top 100 DEGs comparisons are detailed in Supplementary Fig. 8), demonstrate the consistency of gene expression patterns between in silico and real treatment markers. Known IPF markers such as *IL33*^125^, *ADAM12*^126^ and *CXCL8*^127^ exhibit similar changes in gene expression in both real treatment experiments and in silico predictions. The *R*^2^ scores and side-by-side comparisons of real treatments and in silico gene expression of the ECM organization pathway further validate the capability of the UNAGI model to accurately simulate in silico perturbations on IPF-related targets (Fig. 6g; all ECM organization pathway genes comparisons are listed in Supplementary Fig. 9). The alignment between in silico drug perturbations and actual drug treatments on the PCLS demonstrates the reliability of UNAGI.

### UNAGI unveils COVID-19 cellular dynamics and therapeutic opportunities

To demonstrate the expansive applicability of UNAGI to various complex diseases, we studied the temporal dynamics of coronavirus disease 2019 (COVID-19). We used a subset of a COVID-19 dataset^128^ consisting of 246,948 peripheral blood mononuclear cells (PBMCs) from 47 age-matched patients with various severities of COVID-19. We categorized them into four COVID-19 stages based on the disease severity of patients: healthy (control, or stage 0), asymptomatic or mild (stage 1), moderate (stage 2) and severe or critical (stage 3). We independently trained the UNAGI framework from scratch on the COVID-19 dataset to reveal temporal dynamics in COVID-19 disease progression and screen potential therapeutic targets.

**Fig. 7:**
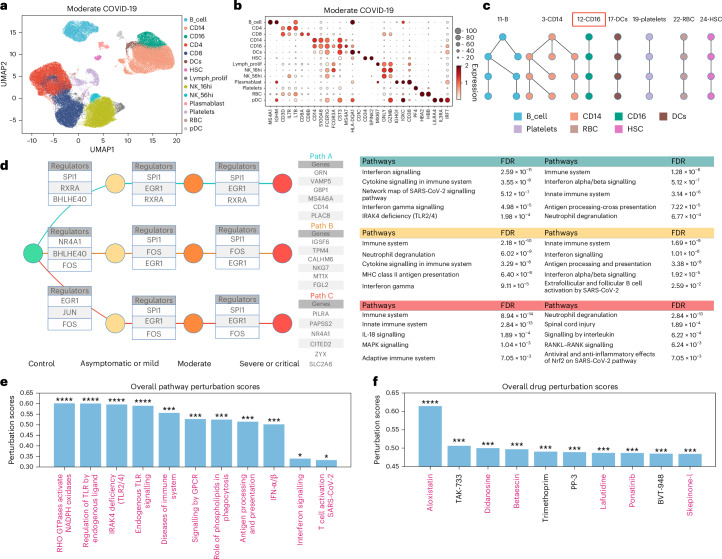
UNAGI in silico analysis unveils COVID-19 cellular dynamics and therapeutic opportunities. **a**, UMAP display of stage 2 COVID-19 data with each dot symbolizing an individual cell. Cells are colour-coded based on their respective cell types. **b**, Dot plot illustrating the expression levels of canonical cell-type markers present within the stage 2 COVID-19 dataset. **c**, Dynamic graphs representing cellular dynamics underlying the COVID-19 progression. Within these graphs, each node corresponds to a cell cluster, and the connecting edges signify the relationships between these nodes (shift of the cell population along with COVID-19 progression). **d**, Depiction of the reconstructed gene regulatory network for track 12-CD16. Prominent gene regulators, genes and pathways discerned from the enrichment analysis are enumerated. **e**, Bar chart detailing the principal pathway perturbation outcomes. Pathways highlighted have literature support, indicating their potential as therapeutic targets against COVID-19. **f**, Bar chart outlining the top 10 drug perturbation results. Drugs that are emphasized have been highlighted based on literature support, suggesting their candidacy for treating COVID-19. We applied FDR correction in **d** using the BH procedure. Asterisks denote statistical significance as follows: *0.01 < FDR < 0.05; ***1 × 10^−4^ < FDR < 1 × 10^−3^; ****FDR < 1 × 10^−4^.

**Extended Data Fig. 3:**
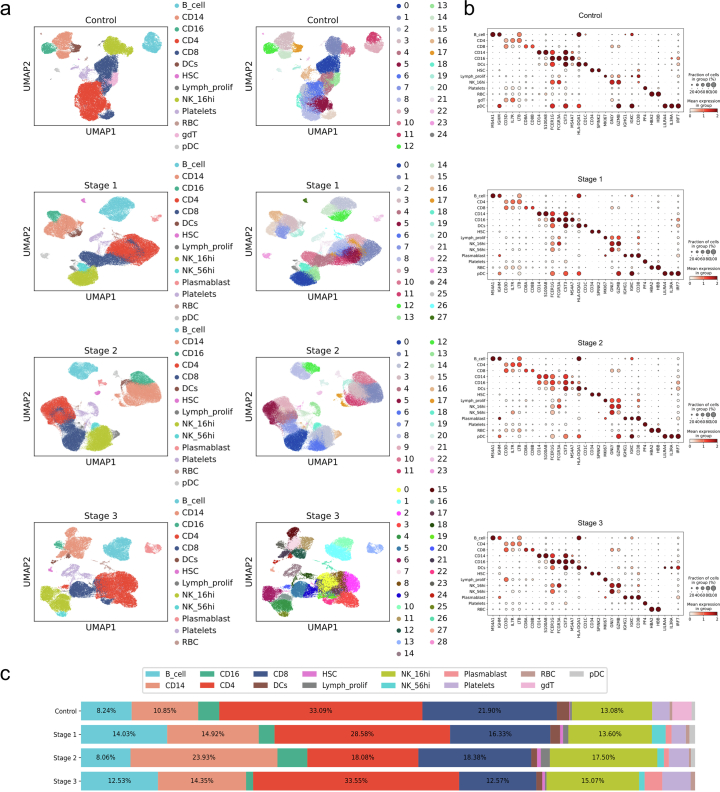
UNAGI identifies progressive heterogenous cell populations across COVID-19 stages. **a**, UMAP visualization of COVID-19 PBMC cells across various COVID-19 stages (Control, Stage 1: Asymptomatic or Mild, Stage 2: Moderate, Stage 3: Severe or Critical). Each point represents a cell, with the first column categorizing cells by type and the second column displaying Leiden cluster IDs. **b**, Gene dot plots of the canonical cell-type biomarkers associated with each of the identified cell types across four COVID-19 stages. The expression is normalized. **c**, Cell type composition chart of COVID-19 dataset. This chart provides a visual representation of changes in cell type composition throughout the progression of COVID-19, with different colors indicating specific cell types.

After learning the latent cell representations (Extended Data Fig. 3), UNAGI identified 14 unique cell populations at stage 2 (Fig. 7a). This spotlights potential biological associations, such as those between platelets and T cells, which align with previous research^128^. Here UNAGI can elucidate cell-type markers for cell populations, such as *MS4A1* and *CD79A* in B cells, and underscore differential expressions, notably *CD8A* and *CD8B*, in CD8 T cells—findings that harmonize with manual annotations (Fig. 7b).

Focusing on the cellular dynamics across the trajectory of COVID-19, UNAGI identified seven distinctive tracks reflecting the evolving cellular interplay across COVID-19 severity levels (Fig. 7c). Figure 7d adds detail by highlighting key genes involved in the progression of the COVID-19 in CD16^+^ monocytes, such as *BHLHE40*, which finds an upregulation in moderate patients^129^, and *EGR1*, recognized for influencing severe acute respiratory syndrome coronavirus 2 (SARS-CoV-2) replication and antiviral responses^130^. Notably, genes such as *GRN*^131^ and *PLAC8*^132^ emerge as upregulated in COVID-19. Gene enrichment analyses further discern crucial pathways tied to the disease such as interferon signalling and immune system pathways^133–135^. Transitioning to predictive capabilities, UNAGI identified potential therapeutic pathways such as the RHO GTPases Activate NADPH Oxidases pathway, which aligns with modern findings emphasizing its substantial role in COVID-19^136,137^ (Fig. 7e). A deep dive into pathways related to Toll-like receptors and interferon responses^138^ further broadens the therapeutic landscape.

Figure 7f shows the in silico drug perturbation results predicted by UNAGI. Aloxistatin stands out, achieving the highest drug perturbation scores and drawing attention owing to its potential against SARS-CoV-2 proteases^139^. In addition, didanosine, notable for its efficacy against COVID-19 polymerase and exonuclease^140^, and ponatinib are recognized as potent COVID-19 drugs by other machine learning methods^141^, aligning with several other recent published studies^139–143^.

### UNAGI enhances cell embedding and disease dynamics understanding

To demonstrate UNAGI’s advantages over existing methods in understanding the dynamics of diseases, we benchmarked it against established methods, including scVI^19^, GraphSCC^22^, scGEN^33^, scGGAN^20^, scGPT^31^, Geneformer^144^, scGNN^21^, Seurat^16^ and SCANPY^17^, on the IPF dataset and scRNA COVID-19 PBMC data^128^. To present a comprehensive benchmarking, we conducted evaluations on various tasks: (1) generating cell embeddings, (2) computing efficiency, (3) identifying disease markers and (4) identifying disease-associated pathways. Supplementary Table 5 summarizes the functionality and ranks performance of these benchmarked methods across key tasks.

#### Cell embedding benchmarking

**Fig. 8:**
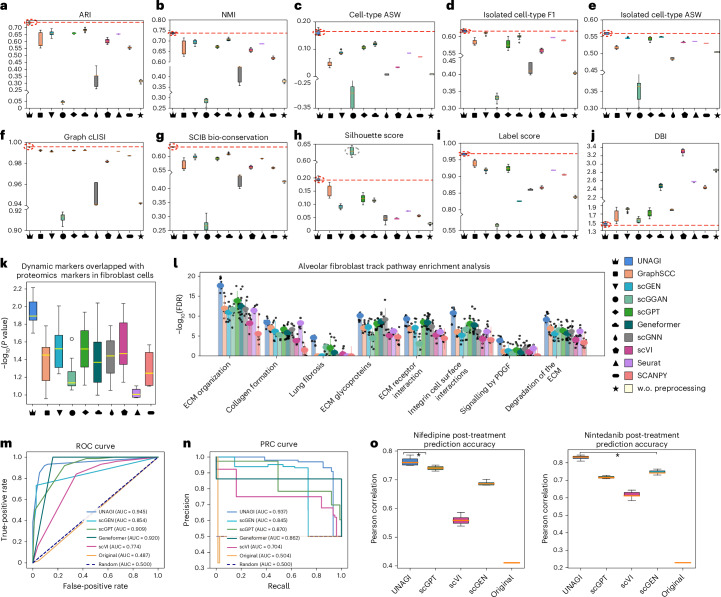
Benchmarking UNAGI against existing methods. **a**, ARI. **b**, NMI. **c**, Cell-type ASW. **d**, Isolated cell-type F1 score. **e**, Isolated cell-type ASW. **f**, Graph cLISI score. **g**, SCIB overall bio-conservation score. **h**, Silhouette score. **i**, Label score. **j**, DBI; a lower DBI signifies better clustering. From left to right, the benchmarking methods are UNAGI, GraphSCC, scGEN, scGGAN, scGPT, Geneformer, scGNN, scVI, Seurat, SCANPY and SCANPY using raw data without rigorous preprocessing. For the meaning of each score, see the ‘Benchmarking’ section in Methods. **k**, Disease marker identification: the box plots illustrate the *P* value resulting from the hypergeometric test of the overlap between proteomics markers and identified disease markers. **l**, Disease-associated pathway identification: bar plots of –log_10_(FDR) significance for each pathway. The experiments in **a**–**l** run with different seeds (*n* = 10). **m**, ROC curves for in silico drug screening performance, showing true-positive rate versus false-positive rate. **n**, PRC curves for precision and recall in in silico drug screening. **o**, Box plots of Pearson correlations between predicted gene expression changes and ex vivo experimental changes for top 100 DEGs (treatment marker genes) after in silico perturbation for nifedipine and nintedanib treatments. In silico perturbations were conducted on the top 10 treatment marker genes, respectively. The experiments in **o** run with different seeds (*n* = 5). The boxes in **a**–**k** and **o** represent the IQRs, and the solid lines indicate the medians. The whiskers extend to points within 1.5 IQRs of the lower and upper quartiles. The error bars in **l** represent s.d. and data are presented as mean values ± s.d. We applied the one-sided hypergeometry test and FDR correction using the BH procedure in **k** and **l**.

**Extended Data Fig. 4:**
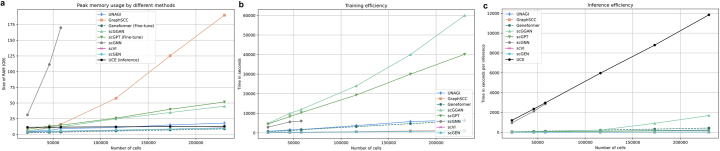
Benchmarking of running efficiency using the IPF dataset. **a**, Benchmarking of the peak memory (CPU) usage by different methods. The x-axis is the number of cells, and the y-axis is the peak memory usage of the method. **b**, Benchmarking of running time. The x-axis is the number of cells, the y-axis is the running time. The peak memory required to run scGNN exceeds the limits of our workstation (256GB RAM) when the number of cells surpasses 60,000. **c**, Benchmarking of inference time. The x-axis is the number of cells, the y-axis is the inference time.

**Extended Data Fig. 5:**
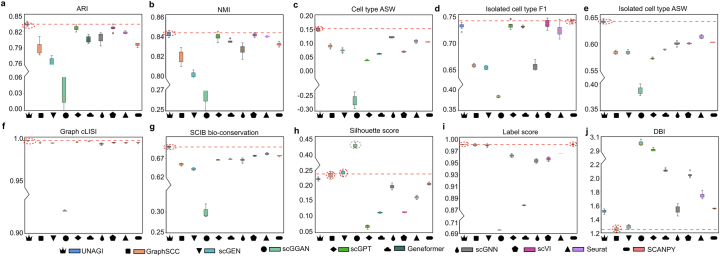
Benchmarking of embedding quality against existing methods on the COVID-19 dataset. **a**, Adjusted Rand Index (ARI). **b**, Normalized Mutual Information (NMI). **c**, Cell type ASW. **d**, Isolated cell type F1 score. **e**, Isolated cell type ASW. **f**, Graph cLISI score. **g**, SCIB overall bio-conservation score. **h**, Silhouette score. **i**, Davis-Bouldin index (DBI); a lower DBI signifies better clustering. **j**, Label score. From left to right, the benchmarking methods are UNAGI, GraphSCC, scGEN, scGGAN, scGPT, Geneformer, scGNN, scVI, Seurat and SCANPY. The boxes represent the interquartile ranges (IQRs), and the solid lines indicate the medians. The whiskers extend to points within 1.5 IQRs of the lower and upper quartiles. The experiments in panels a-j run with different seeds (n = 10).

To evaluate the capability to generate disease-informed cell embeddings, we compared the quality of embeddings generated by different methods through various biological conservation metrics suggested by Luecken et al.^145^. UNAGI consistently outperformed existing single-cell analysis methods on the IPF dataset over various benchmarks, except for the silhouette score (Fig. 8a–j). Although scGGAN achieved the highest silhouette score, it fell short on metrics related to cell-type specificity, illustrating that its embeddings do not adequately capture the underlying biological variation (Supplementary Fig. 10). UNAGI outperformed other methods in generating cell-type distinct embeddings. This was evidenced by the highest cell-type-associated metrics, including a 5.15% higher ARI, a 4.30% higher cell-type average silhouette width (ASW) and a 2.97% higher NMI, compared with the second-best methods. scGNN can only work on downsampled datasets because of its memory-hungry features (Extended Data Fig. 4a) and our experiments comparing UNAGI and scGNN with the same 25% downsampled dataset setting suggested that scGNN’s inadequate performance was not a bias caused by the reduced data size (Supplementary Fig. 11). scGPT and Geneformer pretrained on large-scale single-cell dataset and fine-tuned on the IPF dataset can achieve the joint second-best overall performance. Our comprehensive benchmarking also demonstrates that UNAGI outperforms these foundation models in both zero-shot and fine-tuning settings (Supplementary Fig. 12). The results of SCANPY using the standardized single-cell analysis pipeline on raw data without preprocessing (w.o. preprocessing) strength the need to perform rigorous data cleaning and normalization for analysing the complex single-cell data. The UMAP visualizations of the benchmarking methods applied to the IPF dataset are presented in Supplementary Fig. 13. However, the COVID-19 data is less noisy and complex, and better fits a zero-inflated negative binomial (ZINB) distribution. In general, UNAGI achieved similar or better performance compared with existing methods (Extended Data Fig. 5). Besides achieving high performance in ARI, NMI and label scores like other methods, it surpasses them by achieving a 2.75% higher cell-type ASW and a 2.81% higher isolated label silhouette score. The benchmarking results of embedding quality highlighted that UNAGI can generate more disease-informed cell embeddings than existing methods. Besides the high performance, UNAGI is also computation efficient and strikes a balance between memory demands and execution time in large-scale single-cell datasets compared with benchmarked methods (Extended Data Fig. 4a,b).

#### Disease marker and disease-associated pathway identification benchmarking

Beyond better cell embeddings, UNAGI also outperforms benchmarking methods in identifying disease-associated markers and pathways. This is attributed to the learned disease-informative embeddings and the iterative training strategy, which emphasizes disease markers during optimization. Figure 8k shows that UNAGI’s disease markers have stronger agreement with proteomics markers in the fibroblast cells. Besides the diminished embedding quality relative to UNAGI, the existing techniques were not equipped with iterative training and often not consider the longitudinal disease progression information during the optimization process. This is due to the insufficient information exchange between cell embedding learning and gene regulatory network inference. Consequently, their comprehension of the disease’s advancement cannot match that of UNAGI. In addition, through benchmarking UNAGI with existing methods on disease-associated pathway discovery tasks, we demonstrated that UNAGI can better reveal the biological process underlying the development of disease. Similar to the dynamic marker discovery, UNAGI consistently outperforms existing methods by achieving more significant FDR for the detection of disease-associated pathways (Fig. 8l). Notably, UNAGI stands out for its ability to detect the lung fibrosis pathway from the alveolar fibroblast track, something that other methods struggle with.

### UNAGI outperforms existing methods in unsupervised in silico drug perturbation

The UNAGI framework’s nonlinear nature and its capability to understand the temporal gene regulatory networks (GRNs) of the disease progression help it surpass other methods in performing the in silico drug perturbation. We conducted benchmarking experiments on (1) in silico drug screening and (2) post-treatment gene expression changes prediction tasks to show that UNAGI outperforms existing methods in the unsupervised in silico drug perturbation.

#### In silico drug screening benchmarking

We benchmarked UNAGI against scGPT, Geneformer, scVI and scGEN, and directly calculated the shifts in the gene space (denoted as ‘Original’) on the in silico drug screening task using simulation data. These benchmarked methods do not natively support unsupervised in silico drug screening, so we integrated them into the UNAGI framework to enable this functionality and facilitate a comparative analysis. To conduct the simulation study, we created positive and negative simulation datasets using 25 drugs with the lowest target gene expressions. This involved shuffling the gene expression of individual tissue fibrosis grades and adding signals or random noise to the targets of one simulated drug in fibroblast cells. We then performed in silico perturbation of the implanted drugs using pre-trained models on the simulation datasets to obtain perturbed cell embeddings and calculate the perturbation score. The FDR-BH (Benjamini–Hochberg) of the perturbation score was calculated against the distribution of random perturbation scores to determine whether the models could identify the implanted drugs (see ‘In silico drug screening simulation’ in Methods). UNAGI obtained an area under the receiver operating characteristic curve (AUROC) of 0.945 and an area under the precision-recall curve (AUPRC) of 0.937, 3.6% and 6.7% higher than the second-best method (Fig. 8m,n). The performance of linear methods ‘Original’ (AUROC of 0.487 and AUPRC of 0.504) is close to random and far below UNAGI’s performance. The poor performance of linear methods is caused by overlooking the downstream effects of GRNs and lacking understanding of critical genes underlying disease progression. Nonlinear methods scVI (AUROC, 0.774; AUPRC, 0.704) and scGEN (AUROC, 0.854; AUPRC, 0.845) performed much better than linear methods; they still fall short of matching UNAGI’s performance owing to a lack of understanding of the disease progression mechanisms. We further benchmarked UNAGI with single-cell foundation models, scGPT and Geneformer, in in silico drug screening tasks using both zero-shot and fine-tuned settings. We found that fine-tuning on the IPF dataset indeed improved their performance compared with zero-shot settings (Supplementary Fig. 14). However, UNAGI still achieved higher performance in the in silico drug screening task (AUROC of 0.945, AUPRC of 0.937) compared with fine-tuned scGPT (AUROC 0.909, AUPRC 0.870) and Geneformer (AUROC 0.920, AUPRC 0.862) on the IPF dataset. The improved performance of UNAGI can be attributed to its nonlinear nature to simulate the downstream effects of GRN in the perturbation and the iterative training strategy to improve the power of nonlinear layers by providing a better understanding of the GRN underlying the disease progression mechanism.

#### In silico post-treatment prediction benchmarking

Moreover, we conducted a benchmark of UNAGI against scGPT, scGEN and scVI for predicting gene expression changes after treatments using the snRNA-seq PCLS dataset (Fig. 8o). We also directly modified the gene expression of the top treatment markers (‘Original’) as the baseline to evaluate UNAGI. We trained UNAGI, scVI and scGEN on the control and fibrosis cells, and perturbed the top 10 nifedipine and nintedanib treatment markers (that is, DEGs after ex vivo treatments) to predict the outcome of ex vivo treatments. To predict the post-treatment gene expression, the decoder of UNAGI can map the perturbed cell embeddings to the gene space. Note that the above three methods (UNAGI, scVI and scGEN) were run in an unsupervised manner to predict gene expression changes after treatment and were not exposed to the actual post-treatment single-cell data from the ex vivo experiments. By contrast, the fine-tuning for the scGPT method was conducted with supervision using the fibrosis cells and drug-treated cells because training its perturbation module requires cells before and after intervention. We split the data into training and testing sets and fine-tuned the method on the training set. To predict the post-treatment gene expression, we applied the fine-tuned scGPT model on the testing set and perturbed the top 10 nintedanib or nifedipine treatment markers. Directly modifying the gene expression of the top 10 disease markers (‘Original’) does not enable accurate prediction of perturbation outcomes. Compared with ‘Original’, UNAGI’s Pearson correlation improves 34.4% in the nifedipine treatment prediction and 60.3% in the nintedanib treatment prediction. Compared with scVI and scGEN, UNAGI can more accurately predict the outcome of the top 100 ex vivo treatment markers. UNAGI achieved 6.99% and 9.03% improvements in the Pearson correlation of nifedipine and nintedanib treatment marker predictions compared with other unsupervised methods, respectively. Furthermore, UNAGI even outperformed scGPT, the supervised perturbation prediction method, with a margin of 2.29% and 11.7% in these two treatments, respectively. The improved performance can be attributed to the gene-weight mechanism and the iterative training strategy. Supplementary Fig. 15a shows that treatment markers were assigned higher weights in UNAGI, while they were treated equally with other less important genes in other benchmarked methods. For instance, the median gene weight of the top 100 nifedipine treatment markers is the 81st percentile of all genes’ weights in UNAGI. The pathway enriched in the top 100 weighted genes is closely associated with the development of lung fibrosis, including the TGFβ signalling pathway, elastic fibre formation and ECM organization (Supplementary Fig. 15b). Through the analysis of the bio-conservation of highly weighted genes, we demonstrated UNAGI’s ability in understanding the temporal GRN of disease progression mechanisms. As a result, UNAGI enables more precise predictions of post-treatment gene expression.

### Evaluation of the contribution of UNAGI’s modules through ablations

**Extended Data Fig. 6:**
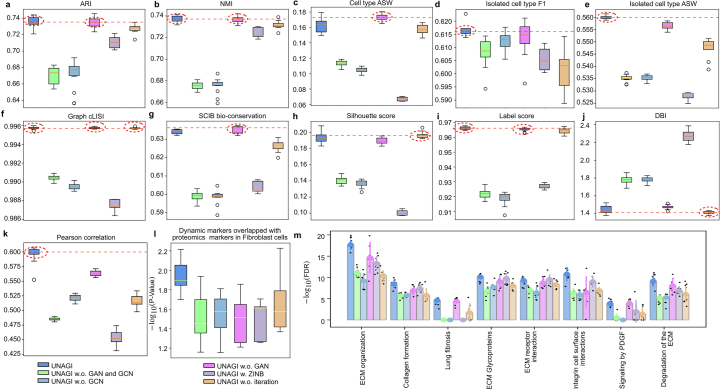
Ablation study to analyze the contribution of individual components to UNAGI’s performance. **a**, Adjusted Rand Index (ARI). **b**, Normalized Mutual Information (NMI). **c**, Cell type ASW. **d**, Isolated cell type F1 score. **e**, Isolated cell type ASW. **f**, Graph cLISI score. **g**, SCIB overall bio-conservation score. **h**, Silhouette score. **i**, Label score. **j**, Davis-Bouldin index (DBI); a lower DBI signifies better clustering. **k**, evaluates the similarity between original cells and generated cells. **l**, Benchmarking of the ability to identify disease markers. Boxplots illustrate the P-value resulting from the hypergeometric test of the overlap between proteomics markers and identified disease markers. **m**, Benchmarking of the ability to identify disease-associated pathways. The experiments in panels a-m run with different seeds (n = 10). The bar plots show the –log10(FDR) of the significance of each identified pathway. The boxes in a-l represent the interquartile ranges (IQRs), and the solid lines indicate the medians. The whiskers extend to points within 1.5 IQRs of the lower and upper quartiles. The error bars in m represent standard deviation (SD) and data are presented as mean values +/− SD. We applied one-sided hypergeometry test and FDR correction using the BH procedure in panels l and m.

**Extended Data Fig. 7:**
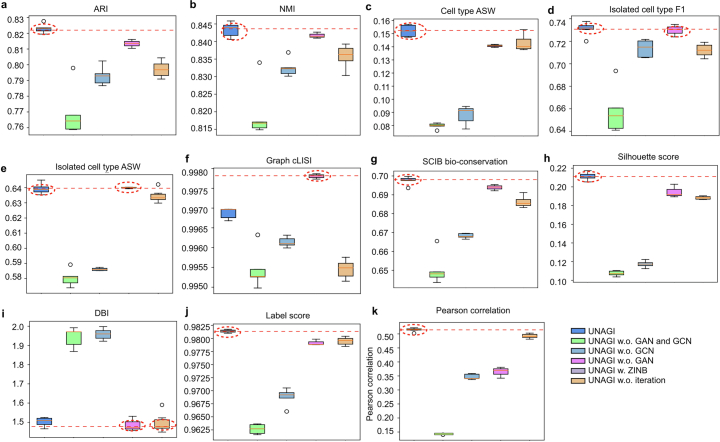
Ablation studies show the contribution of individual components to the embedding quality using the COVID-19 dataset. **a**, Adjusted Rand Index (ARI). **b**, Normalized Mutual Information (NMI). **c**, Cell type ASW. **d**, Isolated cell type F1 score. **e**, Isolated cell type ASW. **f**, Graph cLISI score. **g**, SCIB overall bio-conservation score. **h**, Silhouette score. **i**, Davis-Bouldin index (DBI); a lower DBI signifies better clustering. **j**, Label score; **k**, evaluates the similarity between original cells and generated cells. From left to right, the ablation models are UNAGI, UNAGI without GAN and GCN component, UNAGI without GCN layers, UNAGI without the GAN module, UNAGI with the ZINB distribution, and Train UNAGI directly to the convergency without applying the iterative training strategy. The experiments in panels **a**-**k** run with different seeds (n = 10). The boxes in panels **a**-**l** represent the interquartile ranges (IQRs), and the solid lines indicate the medians. The whiskers extend to points within 1.5 IQRs of the lower and upper quartiles.

We performed a comprehensive analysis using IPF (Extended Data Fig. 6) and COVID-19 (Extended Data Fig. 7) datasets to investigate the impact of individual components on the performance of UNAGI. The ablation study was conducted in the following three aspects: (1) embedding quality, (2) cell generation and (3) disease marker and disease-associated pathway identification.

#### Embedding quality

In terms of the embedding quality, the largest contribution comes from the GCN layers (Extended Data Figs. 6a–j and 7a–j). GCN consistently improves the performance by at least 3.69% across various metrics, including ARI, NMI, label score, silhouette score, cell-type ASW and SCIB (single-cell integration benchmarking) overall bio-conservation score^145^. Compared with using ZINB distribution, the common practice, the results show that the ZILN distribution better fits the IPF dataset, thus leading to better embeddings. In addition to UNAGI, we demonstrated that ZILN distribution can enhance the performance of other methods, such as scVI (Supplementary Fig. 16). The iterative training strategy can also further improve the quality of IPF cells embedding evidenced by achieving higher ARI, isolated label F1 score and isolated label ASW than direct training to the convergence. The UMAP visualization of the ablation studies on the IPF dataset is shown in Supplementary Fig. 17.

#### Cell generation

While the GAN component did not necessarily improve embedding quality (Extended Data Figs. 6a–j and 7a–j), it played a crucial role in guiding the VAE to generate high-quality cells. This contribution is evident in the improved Pearson correlation between the PCA embeddings of the original and generated cells when using the GAN module compared with UNAGI without (w.o.) GAN, which increased by 3.80% in the IPF dataset and 14.57% in the COVID-19 dataset (Extended Data Figs. 6k and 7k).

#### Disease marker and disease-associated pathway identification

Beyond the cell embeddings and cell generation, we conducted ablation studies to evaluate the impact of individual components on disease progression understanding through disease marker discovery and disease-associated pathway identification. The ablation studies on dynamic marker discovery and fibrosis-associated pathway discovery revealed that iterative training is the key factor in understanding the disease (Extended Data Fig. 6l,m). Apart from adopting the iterative training strategy, all ablation models achieved similar performance in the dynamic marker discovery. In the disease-associated pathway identification tasks, the GCN layers improve the model’s performance by incorporating neighbouring cell information into biological activities that failed to be captured in the sequencing process.

## Discussion

In this paper, we describe UNAGI, a computational tool for modelling the temporal cellular dynamics of the complex disease progression. UNAGI leverages the graph VAE-GAN model to handle high-dimensional single-cell data and extract latent embeddings, crucial for formulating progression tracks and reconstructing temporal GRNs. Applied to IPF, UNAGI enables high-resolution modelling of cellular trajectories, key gene regulators and genes associated with progressive lung fibrosis. Through iterative training, it focuses on IPF-specific features, simulating and evaluating perturbations on potential target genes and drugs. UNAGI provides an in-depth understanding of cellular dynamics and GRNs, identifying potential therapeutic pathways and drugs for IPF, showcasing its potential in disease modelling and therapeutic development.

UNAGI differentiates itself from other methods owing to its ability to comprehensively model disease progression and identify potential therapeutic targets through in silico perturbations. UNAGI offers a suite of characteristics that distinguish it in the domain of disease comprehension and therapeutic discovery. UNAGI can create disease-focused cell embeddings and generate cells using a deep generative neural network. This precision enhances cell clustering and identification, surpassing existing methods focused primarily on generic cell representation learning.

UNAGI unravels the intricate cellular dynamics associated with disease progression using the GRN reconstruction module. By generating cell embeddings, UNAGI constructs a ‘cellular dynamics tree’ that maps the transitions of various cell states and populations as the disease advances. This approach incorporates key genes, including dynamic markers and gene regulators, integral to specific disease progression. Consequently, UNAGI identifies underlying GRNs governing these cellular dynamics, highlighting potential biomarkers and therapeutic targets.

Different from other existing methods, the graph VAE-GAN model in UNAGI benefits from the causal insights provided by the GRN reconstruction module, which improves the interpretability of its latent space and reconstruction. Toggling between graph VAE-GAN and the GRN reconstruction model allows UNAGI to integrate the strengths of associative learning and causal inference, leading to more accurate disease progression modelling and interpretation.

Finally, UNAGI generates cell embeddings by leveraging its understanding of disease progression mechanisms. This enables in silico perturbations, unsupervised analysis of pathways and drug perturbations, and distinguishes it from existing methods owing to its comprehension of disease progression. This allows for the identification of potential therapeutic pathways and potential drug candidates without needing pre-existing drug perturbation training datasets, which are often difficult to acquire. Its unsupervised nature enhances applicability and practicality across various complex diseases, offering an advantage over many current approaches that rely on supervised learning and extensive training sets.

UNAGI can yield a full spectrum of outcomes, from well-supported findings to unexplored hypotheses. It revealed that stromal cells follow specific trajectories during fibrosis progression, notably the marked accumulation of fibroblast cells, which correlates with extensive fibrosis in IPF, while adventitial and alveolar cells are dynamically involved, and vascular endothelial cells decrease as IPF progresses. In addition, UNAGI identified cell-specific gene regulators such as CTCF, EP300 and SMC3, along with dynamic markers such as *COL1A1* and *COL14A1*, and static markers for sub-cell types, such as *NLGN1* and *MFAP5*, for fibroblast adventitial cells, potentially leading to new biomarkers and precise therapies. Furthermore, UNAGI highlighted potential IPF therapeutic pathways, including Netrin-1 signalling and ROBO receptors, and potential drugs such as nifedipine as an anti-fibrotic, as well as identified repurposed drugs for COVID-19, such as aloxistatin and didanosine, demonstrating its broad potential in biomedical research.

Despite its array of abilities, it is imperative to recognize UNAGI’s limitations, especially its dependency on the CMAP database for in silico drug perturbation. The CMAP database, though invaluable, has its set of challenges. It does not encompass all potential drugs and compounds, thereby narrowing UNAGI’s drug screening horizon. In addition, the impact of drug perturbations on a variety of cell types within CMAP remains either inadequately explored or ambiguous. Incorporating a more detailed and expansive drug perturbation or drug target database could amplify UNAGI’s prowess in in silico drug perturbation. Lastly, as different patients may develop distinct disease progression patterns^146^, it is crucial to classify patients into progressors and non-progressors for precision medicine. While the UNAGI model was not specifically developed for this application, it can be customized by incorporating a classifier to predict the patient category from the learned cell embeddings. In addition, UNAGI can predict effective drug candidates through in silico screening, but it is not able to fully elucidate their mechanism of action. However, these efforts are beyond the scope of this study.

UNAGI is an AI-based computational framework designed to uncover distinct cellular trajectories during disease progression, analyse regulatory and perturbation shifts, and predict drugs that can reverse these shifts. We demonstrated its performance on a unique dataset of tissues from patients with IPF, providing detailed observations, proteomic and experimental validations, and its applicability to another disease, COVID-19. The widespread availability of UNAGI is expected to enhance our understanding of complex diseases and accelerate therapeutic development by repositioning known compounds and modelling their effects. Beyond disease-related applications, UNAGI can potentially be applied to developmental systems such as embryogenesis^147^, organogenesis^148^ and neurogenesis^149^ to infer underlying temporal GRNs and identify potential interventions for manipulating cell fates.

## Methods

### Dataset description and preprocessing

#### snRNA-seq IPF dataset

In this study, we used snRNA-seq technology to profile the IPF disease progression. For the advantages of using snRNA-seq over scRNA-seq in this study, see Supplementary Note 4. The snRNA-seq IPF dataset was collected from a total of 19 individuals, comprising 10 healthy donors and 9 patients with IPF. Biobanking was approved by the local medical ethics committee of the KU Leuven University Hospital (ML6385). A secondary approval (number 2000025427) at the Yale Institutional Review Board was obtained. Recognizing that different regions of the lung may be at varying tissue fibrosis grades of disease progression^47^, we utilized cells isolated from these distinct regions within the IPF lung to model the temporal progression of IPF. Altogether, the dataset consists of 30 samples from control subjects and 24 samples from patients with IPF. We elaborated the details of step-by-step data preprocessing and cell-type assignments (the ‘ground truth’ column in Supplementary Fig. 1) of the IPF dataset in Supplementary Note 4. Following the preprocessing, we adopted the stromal cell line that encompassed 231,477 cells and 2,484 genes to validate the UNAGI method.

#### scRNA-seq COVID-19 PBMC dataset

We used an annotated PBMC COVID-19 dataset^128^ containing more than 780,000 cells from 130 patients. We subsetted the dataset by using patients with ages between 50 and 69 to evaluate the generalizability of UNAGI. In total, we have 246,948 cells from 47 patients, 26 of them are males and 21 of them are females. According to the severity of patients, we categorized them into four COVID-19 severity levels. Specifically, 10 patients were categorized as healthy (36,198 cells), and 10 patients were classified as asymptomatic or mild (62,856 cells). The moderate data is composed of 15 patients (97,266 cells), while the severe or critical comprises 12 patients (50,628 cells). In the preprocessing step, we selected the top 6,000 highly variable genes for downstream analysis.

### Graph VAE-GAN

Our UNAGI method introduces a graph VAE-GAN model. To leverage cellular neighbours to diminish the effects of dropouts and noise^21^, we stacked a cell graph convolution (GCN) layer on top of VAE. A graph convolution layer is a specialized type of neural network that can capture the topological structure of data, particularly by identifying features within local neighbourhoods. GCN aggregates cell–cell relationships to construct a graph (*V*,*E*), where *V* denotes the vertices (cells) and *E* represents the edges (connections between cells). To establish this graph, the *K*-nearest neighbours (KNN) algorithm is used to build the connectivity matrix *A*, which defines the similarity between cells. The graph convolution is defined as ${f}_{\mathrm{GCN}}\left(X,A\right)=\alpha \left({AX}{W\;}^{\mathrm{GCN}}\right)$, where *W*^GCN^ refers to the trainable weights of the GCN layer and *α* is the activation function. Importantly, cells from different disease grades (phases of cellular states during disease progression, characterized by patient samples or cells) are not connected in the connectivity graph *A*, maintaining a disease grade-specific cell graph convolution.

UNAGI uses a VAE-based deep-learning model^30^ to model the cellular dynamics behind complex disease progression and simulate the drug perturbations. The VAE’s encoder–decoder structure can model the probability distribution of high-dimensional data in a lower-dimensional space and generate new samples from this reduced-dimensional distribution. As a variational method, it facilitates the in silico perturbation of cells by modulating their gene expressions. To refine the generative ability of VAE, we followed the previous method^150^ to use GAN to guide the generation of VAE with the min–max training strategy^151^. The encoder of the graph VAE-GAN, *E*_*θ*_:*R*^*n*^ → *R*^*l*^, consists of a GCN layer and several multi-layer perceptrons (MLPs). It can transform a cell **x**_**i**_∈*R*^*m*^ to its corresponding *l*-dimensional latent vector **z**_**i**_. The GCN layer takes the normalized cell-by-gene count matrix *X* and connectivity matrix *A*, generating a graph representation *f*_GCN_(*X*,*A*) = *α*(*AXW*^GCN^), where *W*^GCN^ are weights of the GCN layer and *α* is the activation function. Acknowledging that the latent distribution of single-cell data follows a multivariate normal distribution, two MLPs are used to determine the mean vectors ${\mu }_{z}={f}_{{{\rm{\mu }}}_{\theta }}\left({{\rm{\mu }}}_{z}|{\;f}_{\mathrm{GCN}}\left(X,A\right)\right)$ and log-standard deviation vectors $\log {\sigma }_{z}={f}_{{\sigma }_{\theta }}\left(\log {\sigma }_{z}|\;{f}_{\mathrm{GCN}}\left(X,A\right)\right)$ of the latent representation. The standard deviation of the latent representation is ${\sigma }_{z}=\,{e}^{{\sigma }_{z}}$. The latent representation for a cell is represented as ${\mathbf{z}}{{\sim}}{\mathscr{N}}\left({\mu}_{{\mathbf{z}}},{\sigma}_{{\mathbf{z}}}^{2}\right)$, and the approximated posterior distribution is represented as *q*_*θ*_ (*Z*|*X*,*A*).

The decoder ${p}_{\varphi }:{R}^{l}\to {R}^{3n}$ takes *Z* as input to reconstruct the cell-by-gene count matrix. We used the ZILN distribution to model the gene expression. The ZILN model is a composite distribution that integrates two distinct distributions: the first part is a Bernoulli distribution, Bernoulli $\left(\varrho \right)$, which accounts for the dropout events commonly observed in single-cell sequencing. The second component of the ZILN model captures the actual gene expression levels following a log transformation, represented by $\log {\mathscr{N}}\left(\mu ,{\sigma }^{2}\right).$ The likelihood function of a reconstructed cell ${\mathrm{x}}{\in}\,{X}^{m\times n}$, where *m* is the number of cells and *n* is the number of genes in a cell, can be written as

1 $$\begin{array}{l}{p}_{\varphi }({{\mathbf{x}}}|{{\mathbf{z}}})=\mathop{\prod}\limits _{j\in n}{\mathrm{ZILN}}({x}_{{{\mathbf{j}}}}|{{\varrho}}_{j},{\mu}_{j},{\sigma}_{j}^{2})\\=\mathop{\prod}\limits_{j\in n}[{{\varrho}}_{j}{\delta}_{0}({x}_{{{\mathbf{j}}}})+(1-{{\varrho }}_{j}){\mathrm{LN}}({x}_{{{\mathbf{j}}}}|{\mu }_{j},{\sigma}_{j}^{2})(1-{\delta }_{0}({x}_{{{\mathbf{j}}}}))]\end{array}$$

2 $$\begin{array}{l}\mathrm{LN}\left({x}_{{\boldsymbol{\mathrm{j}}}}|{\mu }_{j},{\sigma }_{j}^{2}\right)=\left\{\begin{array}{l}\frac{1}{{x}_{{\boldsymbol{\mathrm{j}}}}{{\rm{\sigma }}}_{j}\sqrt{2\pi }}{e}^{\frac{-{(\mathrm{ln}{x}_{{\boldsymbol{\mathrm{j}}}}-{\mu }_{j})}^{2}}{2{\sigma }_{j}^{2}}},\,\mathrm{if}\,{x}_{{\boldsymbol{\mathrm{j}}}}\,>\,0\\0,\,\qquad\qquad\quad\quad\;\,\mathrm{if}\,{x}_{j}\,=0\end{array}\right.\end{array}$$

3 $$\begin{array}{c}{\delta }_{0}({x}_{{\boldsymbol{\mathrm{j}}}})=\left\{\begin{array}{c}1,\mathrm{if}\,{x}_{{\boldsymbol{\mathrm{j}}}}=0\\ 0,\,\mathrm{if}\,{x}_{{\boldsymbol{\mathrm{j}}}} > 0\end{array}\right.\end{array}$$

To reconstruct the cell-by-gene matrix *X*, the decoder *p*_*φ*_ learns parameters of the ZILN distribution, including the zero-inflation probability ${\varrho }={f}_{{{\varrho }}_{\phi }}({\varrho }|Z)$, scale of the log-normal distribution *σ* for each gene (a vector of learnable parameters) and mean *μ* of the log-normal distribution, denoted as $\mu ={f}_{{\mu }_{\phi }}(\;\mu |Z,\sigma )$. The prior distribution *p*(*Z*) is a multivariate standard normal distribution. Within our framework, we designated the entire graph VAE model as the generator *G*. The loss function of the generator *L*_*G*_ can be formulated as

4 $$\begin{array}{c}{L}_{G}=L(\theta ,\varphi ,X,A)=\mathrm{KL}({q}_{\theta }(Z|X,A)||p(Z\;))-{E}_{{q}_{\theta }(Z|X,A)}[\log {p}_{\varphi }(X|Z\;)]\end{array}$$

The first term of *L*_*G*_ is the Kullback–Leibler (KL) divergences, which quantifies the difference between the latent representation *q*_*θ*_ (*Z*│*X*,*A*) learned by the encoder and the predefined prior distribution *p*(*Z*). The second term is the expected log-likelihood of the input data given the reconstruction generated by the decoder, acting as a reconstruction loss. Together, *L*_*G*_ promotes the model’s generative performance with the probabilistic constraints of the latent space.

To further refine the generative capabilities of the graph VAE, an adversarial discriminator is incorporated into the model’s architecture. This discriminator is a classifier based on MLPs to distinguish between original cells *X* and the reconstructed cells *G*(*X*,*A*) generated by the graph VAE. A min–max adversarial training strategy is then applied, aimed at optimizing the loss function *L*_GAN_:

5 $${L}_{\mathrm{GAN}}=L(X,A)=\mathop{\min}\limits_{G}\,\mathop{\max}\limits_{D}\,{{\mathbb{E}}}_{X}[\log (D(X))]+{{\mathbb{E}}}_{X}[\log (1-D(G(X,A)))]$$

Here *D* is the adversarial discriminator, and *G* is the generator (graph VAE). During the training phase, cells are labelled as real or fake (produced by the generator for the purpose of adversarial training). The discriminator, *D*, is optimized to effectively distinguish between real and fake cell labels, aiming to maximize the probability of correctly identifying real and generated cells. Simultaneously, the second term of *L*_GAN_ incentivizes the generation of cell reconstructions that are highly similar to the original data that *D* cannot distinguish them from real cells. The overall loss function of UNAGI, denoted as *L*, is a composite of the graph VAE loss and the GAN, written as *L* = *L*_*G*_ + *L*_GAN_. Although it appears to suggest that VAE-GAN back propagates the sum of *L*_*G*_ and *L*_GAN_, in practice, the optimization involves distinct phases for each component. Within the same epoch, a two-step optimization is applied: in the first step, the graph VAE-GAN is optimized based on the *L*_*G*_ and the parameters are optimized using ${L}_{\mathrm{GAN}}$ in the second step. By integrating these components, UNAGI harnesses the strengths of various architectures, the GCN can leverage the cell–cell relationship information, the VAE can model the complex single-cell data, and the GAN can refine the quality of cell generation.

### Dynamics graph and underlying GRN inference

UNAGI builds a dynamic graph to illustrate the progression of each cell population (cell type or subtypes) throughout disease progression. We applied Leiden clustering^152^ on the latent embeddings, generated by graph VAE-GAN, to identify distinct cell populations at each disease grade (see Supplementary Note 5 for the clustering parameters optimization strategy). To measure distances between cell populations in adjacent disease grades, we used the KL divergence rather than the Euclidean distance, which can be problematic in high-dimensional data contexts^153,154^. For each cell population (for example, cell type), we approximated its distribution using a Monte Carlo sampling strategy^155^ involving the sampling of each dimension of the latent embeddings a thousand times to form a multivariate normal distribution. The KL divergence is calculated to measure the distance between these populations’ multivariate normal distributions.

In addition, we identified the top 100 DEGs in each cell population. We then calculated DEG distances among cell populations across disease grades. The DEG distance is defined as ${{\mathcal{T}}}_{d}\left({\mathrm{DEG}}_{c1},{\mathrm{DEG}}_{c2}\right)\times \sum _{j\in {\mathrm{DEG}}_{c1}}|{R}_{j}^{c1}-{R}_{j}^{c2}|$, where the first term is the Jaccard distance between ${\mathrm{DEG}}_{c1}$ and ${\mathrm{DEG}}_{c2}$, DEGs of two cell populations. The second term considers the ranking difference between two DEG lists. Here ${R}_{j}^{c1}{\rm{and}}{R}_{j}^{c2}$ represent the ranking of gene *j* in ${\mathrm{DEG}}_{c1}{\rm{and}}{\mathrm{DEG}}_{c2}$, respectively. To render the KL divergence and the distances of DEGs comparable, we implemented min–max normalization for each metric across all potential connections within a specific cluster. After normalization, we represented the distances between each cluster pair as the sum of the normalized KL divergence and the normalized DEG distances. We then compiled these normalized distances for all possible connections across various disease grades to create a background distance distribution. This distribution is essential for assessing the statistical significance of connections between clusters throughout the different grades of the disease. In scenarios where a cluster is connected to more than one cluster in an adjacent grade, the most statistically significant one is used. These significant connections form tracks that trace from the control to the final grade of the disease, defining the disease progression. Consequently, the dynamic graph ${G}_{\mathrm{dynamic}}$ includes these progression tracks, each representing a comprehensive cellular state transition associated with a specific cell population during disease progression.

Moreover, we used iDREM (Interactive Dynamic Regulatory Events Miner)^46^, a machine learning model based on an input–output hidden Markov model, to reconstruct the temporal GRN underlying the reconstructed cellular dynamics graph ${G}_{\mathrm{dynamic}}$ (Supplementary Note 6). iDREM also captures the gene regulators that modulate those gene paths during disease progression. The dynamic genes and gene regulators identified through this process are considered dynamic marker candidates and hold potential as therapeutic targets for the disease.

### Iterative training strategy of UNAGI

The training strategy for UNAGI is structured as an iterative process, consisting of two primary phases that are cyclically repeated: (1) learning cell embeddings using the VAE-GAN framework and (2) constructing a cellular dynamics graph and identifying critical genes and gene regulators. Initially, with the cell embeddings learned with equal importance of all genes in the loss function (generic learning as in existing methods), we used the dynamics graph module to reconstruct the cellular dynamics and identify critical genes that influence disease progression, using the iDREM algorithm. UNAGI establishes a gene-weight table for each cell, increasing the weights of key genes and their regulators to reflect their roles in disease progression. To mitigate cell mis-clustering at initialization, UNAGI uses a weight-decay strategy where genes strongly associated with disease progression retain consistently increasing weights, while noisy genes have their weights progressively reduced in each iteration, preventing their influence from accumulating by the end of training. See Supplementary Note 7 for methodological details. Supplementary Figs. 18–20 illustrate the effectiveness and robustness of this weight-decay-based iterative training strategy.

Next, in the cell embedding learning of the subsequent iteration, the VAE model undergoes fine-tuning with a modified loss function that accentuates the high-weight genes. This enhancement is accomplished by integrating the gene weights in all cells into the reconstruction loss function, thereby shifting the model’s focus from generic genes to those disease-associated genes identified through GRN inference. During each iteration, after the cell embeddings are updated, the cellular dynamics module steps in to rebuild the cellular dynamics graph and the associated GRNs. This step plays a crucial role in refining and updating the disease-associated genes. These enhancements feed back into and improve the cell embedding learning in the next iteration. However, the revised cell embeddings generate an updated cellular dynamics graph and its GRN, offering a deeper understanding of disease progression and potentially advancing the identification of disease-specific genes, which in return improves the cell embedding learning in the next iteration.

Upon model convergence, the highest-weighted genes are associated with the disease and thus indicating that UNAGI can indeed ‘comprehend’ the disease and recognize important disease-relevant genes during the iterative training. For instance, enrichment analysis shows that the top 100 weighted genes are closely associated with IPF (Supplementary Fig. 21). At each training iteration *t*, the gene weights are transformed into a ranking matrix, *R*^*t*^. The objective functions of UNAGI during its iterative training can be then refined as follows to integrate the distilled disease knowledge in the gene-weight table for each cell:

6 $$\begin{array}{l}{L}_{G}^{t}=L({\theta }^{t},{\varphi }^{t},X,A)={\mathrm{KL}}({q}_{{\theta }^{t}}(Z|X,A)||{\rm{p}}(Z))\\ \qquad-{{\mathbb{E}}}_{{q}_{{\theta }^{t}}(Z|X,A)}\left[\log {p}_{{\varphi }^{t}}(X|Z)\left(1+\displaystyle\frac{1}{{({R}^{t})}^{\tau }}\right)\right]\end{array}$$

7 $$\begin{array}{c}{L}_{\mathrm{GAN}}^{t}=L(X,A)=\mathop{\min}\limits_{{G}^{t}}\,\mathop{\max}\limits_{{D}^{t}}\,{{\mathbb{E}}}_{X}[\log ({D}^{t}(X))]+{{\mathbb{E}}}_{X}\left[\log (1-\,{D}^{t}({{\rm{G}}}^{{t}}({X},{A})))\right]\end{array}$$

8 $${L}^{t}={{L}^{t}}_{G}+{{L}^{t}}_{\mathrm{GAN}},t\in \left(0,1,\ldots ,T\;\right)$$

Here *G*^*t*^ represents the generator at the *t*th iteration, and *D*^*t*^ is the discriminator at the same iteration. ${L}_{G}^{t}$ denotes the loss of generator, ${L}_{\mathrm{GAN}}^{t}$ denotes the loss of GAN at the *t*th iteration and *τ* is a hyper-parameter that is responsible for controlling the influence of gene weights on the reconstruction loss (empirically set *τ* as 0.5). UNAGI increases the weights for high-ranking genes to emphasize disease-associated genes and regulators. The weights for low-ranking genes remain roughly unchanged, ensuring that information associated with those genes is not discarded. Through this iterative training, UNAGI progressively improves its ability to generate disease-specific cell embeddings. This approach allows for the identification of disease-specific markers and supports disease-specific in silico perturbations.

### Dynamic and hierarchical static markers discovery

To characterize the temporal progression of the disease for each cell population, UNAGI identifies dynamic markers that are genes that change considerably throughout the disease’s progression. For each track in the cellular dynamics graph, iDREM identifies the gene paths with co-expression patterns during disease progression. Then UNAGI generates the background simulation tracks to identify dynamic markers. This simulation process is repeated *N* times (*N* > 1,000) to establish a random background distribution. We then evaluated the *P* values for each candidate marker based on its accumulated sum fold change against this background distribution. We imposed a more stringent FDR cut-off (FDR < 0.01) than the default (FDR < 0.05). These selected dynamic markers are important in delineating the progression tracks and provide a detailed understanding of the longitudinal evolution of the disease within each distinct cell population.

The hierarchical static marker discovery approach supports the identification of intra-disease grade static markers through hierarchical clustering. UNAGI conducts hierarchical clustering based on the embeddings of cell populations at each disease grade, thereby generating dendrograms to depict the relationships among these populations. In this dendrogram, when focusing on a particular cluster, we analysed it at various levels to identify hierarchical static markers. At lower levels of the dendrogram, the selected cluster compares with a broader range of sibling clusters. Conversely, at higher dendrogram levels, the siblings are more closely related to the selected cluster. This closeness allows for the identification of markers that highlight the subtle heterogeneities among cell subpopulations within the same cell type. For details of marker discovery, see Supplementary Note 8.

### In silico perturbation strategies

In silico perturbation can be executed through two strategies: (1) direct gene expression regulation. This approach involves the direct upregulation or downregulation of specific genes of interest. For a cluster of cells, we defined an expression regulation vector $\Delta =\left[{\Delta }_{g1},{\Delta }_{g2},\ldots ,{\Delta }_{{gn}}\right]$, where each ∆_*gn*_ represents the expression change of gene *gn* (for example, ∆_*g*1_ = 0.5 would indicate an increase in the expression of gene *g*1 by 0.5). The gene expression for a perturbed cell population *X*′_*c*_ can be defined as

9 $$\,{X{\prime} }_{C}=\max \left({X}_{C}+{{\bf{1}}}_{{M}_{c}}\Delta ,0\right)$$

Here *X*_*c*_ represents the original cell-by-gene matrix of a cell population *c*, and *M*_*c*_ represents the number of cells within the cell population. (2) Gene interaction (GI) network-based regulation allows simulating the downstream effects of GRNs. In this strategy, we could regulate the genes of interest and their interacting partners based on the GI network. If one gene expression is changed, the changes are transmitted to connected gene in the GI networks according to the influence factor *I* between them. The GI networks were built based on the HIPPIE database^156^ and STRINGDB^157^. From these two databases, we obtained the strength of GIs *γ* of different gene pairs. For a certain cell population *c*, we transformed the cell-by-gene matrix *X*_*c*_ into a gene-by-cell matrix *Y*_*c*_ and used PCA to generate low-dimensional embeddings *P*_gene_ for each gene across the cell population. The influence factor *I*(*Q*,*R*)∈(−1,1) quantifies the extent to which the perturbation of a given gene *Q* impacts on another gene *R*. *I*(*Q*,*R*) is defined as

10 $$\begin{array}{l}I(Q,R)=\left\{\begin{array}{l}0,\,{\mathrm{if}}\,{{Q}}\,{\mathrm{and}}\,{{R}}\,{\mathrm{are}}\,{\mathrm{not}}\,{\mathrm{connected}}\,\\{\mathrm{sgn}}({\mathrm{cor}}({\;{\boldsymbol{\mathrm{y}}}}_{Q},{{\boldsymbol{\mathrm{y}}}}_{R})){\mathrm{exp}}\left(-w_\mathrm{s} \frac{{\Vert {P}_{Q}-{P}_{R}\Vert }_{2}}{{\prod }_{k\in (Q,R)}{\gamma }_{k}}\right),{\mathrm{otherwise}}\end{array}\,\right.\end{array}$$

11 $$\begin{array}{c}{\mathrm{sgn}}(x)=\left\{\begin{array}{c}1,x > 0\\ 0,x=0\\ -1,x < 0\end{array}\right.\end{array}$$

Here **y**_*Q*_ and **y**_*R*_ are gene expression vectors of genes *Q* and *R*, respectively, in the *Y*_*c*_. The term (*Q*,*R*) denotes a sequence of hops from *Q* to *R* in the GI network, *γ*_*k*_ denotes the strength of GIs of a hop in (*Q*,*R*), *w*_s_ is the steepness weight (*w*_s_ > 0 and empirically set to 0.2 by default) to control the influence factor, cor(***y***_*Q*_,***y***_*R*_) quantifies the correlation between two genes, and sgn(*x*) indicates the direction of their interactions. The gene of interest tends to impose higher impacts on genes that directly interact with. Conversely, genes that are further away in the GI network are less influenced. When regulating a specific gene *η* by changing a certain magnitude Δ_*η*_ (for example, Δ_*η*_ = −0.5 can decrease the expression of gene *η* by 0.5). The expression regulation vector for this scenario is formulated as $$\Delta =\left[{\Delta }_{\eta }I\left(\eta ,{g}_{1}\right),\right.$$$$\left.{\Delta }_{\eta }I\left(\eta ,{g}_{2}\right),\ldots ,{\Delta}_{\eta }I\left(\eta ,{g}_{n}\right)\right]$$. If multiple genes $${G}_{P}$$ are perturbed with individual magnitudes, the expression regulation vector is

12 $$\begin{array}{c}\varDelta =\left[\mathop{\sum}\limits _{i{\epsilon }{G}_{P}\,}{\varDelta }_{i}I(i,{g}_{1}),\mathop{\sum}\limits _{i{\epsilon }{G}_{P}\,}{\varDelta }_{i}I(i,\,{g}_{2}),\ldots ,\mathop{\sum}\limits _{i{\epsilon }{G}_{P}\,}{\varDelta }_{i}I(i,{g}_{n})\right]\end{array}$$

The gene expression for a perturbed cell population *X*′_*c*_ is then calculated as defined in equation (9). Not only does the GI-based in silico perturbation impact genes that are not direct drug targets, but the nonlinearity feature of deep neural networks can also affect indirect target genes (even only directly changing the expression of drug targets). The graph VAE model can extract the gene–gene relationships within a cell and reconstruct cells based on these features. When perturbing genes using the pre-trained encoder, the nonlinear architecture helps propagate the expression changes to downstream targets. This is facilitated by the weights and biases of the encoder, which are optimized to describe the gene regulatory information within the cell. This mechanism allows the model to simulate the downstream effects of GI networks, thereby impacting other genes at the cell embedding level by modifying only a few genes.

### In silico perturbation scoring

We performed perturbations on every disease grade of individual tracks using the perturbed cell-by-gene expression matrix *X*′. This matrix *X*′ is fed into the encoder of the graph VAE-GAN, yielding the perturbed latent cell representation *Z*′ = *E*_*θ*_(*X*′,*A*). The efficacy of these perturbations is assessed by examining the changes in the distances between cell populations within the latent cell embedding space. Specifically, the distance between two cell populations in the latent space *Z* can be quantified as ${{\rm{\delta }}}_{{i}{\prime} ,\;{j}}={\Vert {Z}_{i}^{{\prime} }-{Z}_{j}\Vert }_{2}$, where *i*′ is the perturbed cell population and *j* is another cell population within the same track. The perturbation score of a track *S*_track_∈[−1, 1] at a perturbed disease grade *i* is defined as

13 $${S}_{\mathrm{track}}\left(i\right)=\frac{1}{T}\mathop{\sum }\limits_{j=0,\;j\ne i}^{T}\left(1-\frac{2}{1+\exp \left(w\left({\delta }_{{i}^{{\prime} },\;j}-{\delta }_{i,\;j}\right)\mathrm{sgn}\left(j-i\right)\right)}\right)$$

Here *T* represents the total number of disease grades, *i* is the perturbed disease grade, *w* is a hyper-parameter to control the scaling (empirically, *w* is set as 100 in our case), *δ*_*i*,*j*_ is the distance between disease grades *j* and *i* (unperturbed), and *δ*_*i*′,*j*_ is the distance between disease grades *j* and *i* (perturbed). The function sgn(*x*) (as defined in equation (11)) is a perturbation indicator function to ensure the perturbed cell population that comes closer to the control grade will always have a positive and higher score while moving away leads to a negative and lower score. In addition to track-level perturbation scoring, an overall score *S* assesses perturbation effects across all tracks. This overall score is normalized based on the proportion of cells in each perturbed track within the dataset. It also incorporates the gene-regulating directions of compounds, as indicated in the relevant database, including their reversed directions. The overall score *S* for all disease grades is defined as follows:

14 $$\begin{array}{c}S=\mathop{\sum}\limits _{h\in \mathrm{tracks}}\frac{{N}_{h}}{N}\mathop{\sum}\limits _{i\in \mathrm{stages}}\frac{|{S}_{h}^{{\mathscr{A}}}(i)-{S}_{h}^{ {\mathcal B} }(i)|}{2}\end{array}$$

where ${\mathscr{A}}$ represents the perturbation direction that aligns with the reported direction of the drug target expression change, while ${\mathscr{B}}$ denotes the opposite drug target expression change direction as reported in the CMAP database. The overall score *S*∈[0, 1] is calculated by considering in silico perturbations in both directions, enhancing robustness. This approach is based on the premise that perturbing the targets of an effective drug in opposite directions should lead to a higher ${S}_{h}^{{\mathscr{A}}}\left(i\right)$ and lower ${S}_{h}^{{\mathscr{B}}}\left(i\right)$, resulting in an increased score *S*. *N* here is the total number of cells and *N*_*h*_ is the number of cells in the perturbed track.

### Therapeutic pathways screening

We used pathway data from REACTOME^158^, MatrisomeDB^159^ and KEGG^160^ databases, providing lists of genes associated with various biological pathways. Since the set of genes in individual single-cell transcriptome datasets can vary, we only included expressed genes of pathway targets after preprocessing for in silico pathway perturbations. We applied the scoring and ranking strategies as discussed in the ‘In silico perturbation strategies’ and ‘In silico perturbation scoring’ sections above to identify potential therapeutic pathways. To assess the significance of our in silico pathway perturbations, we established a random background dataset by randomly sampling *n* genes 1,000 times, where *n* is set to the median number of genes across all pathways. The perturbation strength Δ used for random background perturbations was matched to that used for the actual pathway in silico perturbations. We executed in silico perturbations using the random dataset described above to generate a random background therapeutic score distribution. By contrasting the perturbation scores with this background distribution, we could ascertain the statistical significance of the in silico pathway perturbations. This approach aids in identifying potential therapeutic pathways with an FDR-BH of less than 0.05. To further validate the robustness of our pathway perturbation strategy, we conducted a simulation study using the Netrin-1 pathway. We replaced 15% of the genes in this pathway with random genes and conducted in silico perturbations, comparing these results with perturbations using a completely random set of genes. Across 100 experiments with different random seeds, the median perturbation score of the modified Netrin-1 pathway remained very close to the original score (0.6351 versus 0.6548), while the random gene sets scored considerably lower (Supplementary Fig. 22).

### Candidate drugs and compounds screening

We used compounds and their target genes from the CMAP database^34,35^, which contains 34,396 compound or drug profiles. Similar to the pathway perturbation, we used expressed genes after preprocessing and are listed as drugs’ targets for in silico drug perturbations. We applied the scoring and ranking strategies as discussed in the ‘In silico perturbation strategies’ and ‘In silico perturbation scoring’ sections above to identify potential drug candidates. The method for calculating the statistical significance of in silico drug perturbations was akin to that used for therapeutic pathway perturbations, as mentioned previously. The primary distinction lies in the number of genes selected for creating the random background score distribution.

### Verify UNAGI biomarkers by proteomics data

Proteins were extracted from pulmonary tissues using the MPLEx protocol^161–164^. Thirty tissue blocks from IPF donors and 10 from control donors were used. For detailed experiments, protocols and data preprocessing, see Supplementary Note 9. After preprocessing, we adopted a more stringent FDR cut-off (FDR < 0.01) than the default (FDR < 0.05) to identify highly confident dynamic proteins. To verify the temporal dynamic markers determined for each progression track, we applied hypergeometric testing. This test assessed the overlapping ratio between dynamic proteins and dynamic markers. The overlapping between these two marker lists associated with a track is considered statistically significant if the FDR from the hypergeometric test is less than 0.05. We then used heat maps to visualize the LFQ intensities and gene expression from proteomics data and snRNA-seq data, respectively.

### PCLS experiments

To assess UNAGI predictions in a human-relevant context, we utilized PCLS. Recent studies suggest that PCLS provides a more accurate representation of human IPF compared with traditional animal models^165^. The commonly used bleomycin mouse model suffers from notable discrepancies between human and mouse biology, particularly in the context of human pulmonary fibrosis^166–168^. We adopted nifedipine in our PCLS experiments because nifedipine or any other calcium entry blockers are not on the radar for pulmonary fibrosis drug development and nifedipine’s anti-fibrotic effectiveness had not been tested in human samples before. Therefore, PCLS serves as an important tool for providing a more human-relevant model to investigate the anti-fibrotic efficacy of nifedipine^165,169^.

Fresh lung tissue of explanted donor lungs was used for human PCLS according to previously published protocols^44,123,170^. Donor lung samples were sourced from six males and four females and were obtained from the Center for Organ Recovery and Education (CORE) at the University of Pittsburgh. Donor lung samples originated from lungs deemed unsuitable for organ transplantation. For the fibrosis induction in hPCLS, PCLS were treated for 5 days with a control cocktail (CC), including all vehicles or a pro-fibrotic cocktail (FC) consisting of TGFβ (5 ng ml^−1^, Bio-Techne), PDGF-AB (10 ng ml^−1^, Thermo Fisher), TNF-α (10 ng ml^−1^, Bio-Techne) and LPA (5 µM, Cayman Chemical) as described before^123,171^. For drug treatments, PCLS were treated with FC allowing for the induction of fibrosis, and drug treatment started at day 3 until day 5. At the end of the experiment, PCLS were snap-frozen individually in liquid nitrogen for single-nuclei analysis, as described above. The study was approved by the University of Pittsburgh (IRB PRO14010265). Written informed consent was obtained for all study participants. Nuclei were extracted using the Nuclei Isolation kit (CG000505, 10x Genomics). Nuclei (20,000) were loaded on a Chip G with Chromium Single Cell 3′ v3.1 gel beads and reagents (3′ GEX v3.1, 10x Genomics). Final libraries were analysed on an Agilent Bioanalyzer High Sensitivity DNA chip for qualitative control purposes. cDNA libraries were sequenced on a HiSeq 4000 Illumina platform aiming for 150 million reads per library and a sequencing configuration of 26 base pair (bp) on read1 and 98 bp on read2. We used Cell Ranger^124^ (v4.0.0), Cutadapt^172^ (4.1) and STAR (v2.7.9a) to build fastq reads, contaminant trimming and reads alignment. Then we used Seurat for data preprocessing (see Supplementary Note 10 for details).

We then applied the graph VAE-GAN to learn the latent embeddings of the PCLS data. To quantify the effects after treating the fibrosis cells with the drugs, we calculated the pairwise Euclidean distance from control cells to real treatment cells and fibrosis cells in the reduced latent space. We used the difference between the centroid of fibrosis cells and the centroids of real treatments as the perturbation strength vector Δ. We conducted in silico drug perturbations on fibrosis cells using a consistent perturbation strength Δ. The efficacy of these in silico perturbations was evaluated through UMAP visualizations and by measuring the pairwise Euclidean distances between cell embeddings in latent space. Our primary objective was to ascertain if in silico drug perturbations could replicate the cell embeddings in latent space as observed with actual drug treatments, thereby validating the accuracy of UNAGI-driven in silico drug perturbations. In addition, to compare the similarity of the differential genes associated with the in silico drug perturbations (in silico drug perturbation versus fibrosis) and those of real drug treatment (drug versus fibrosis), we used RRHO plots. Moreover, box plots and the *R*^2^ score with *F*-test were used as analytical tools to quantify gene expression similarities between cells under actual drug treatments and cells produced from our in silico perturbations for both nintedanib and nifedipine.

### Benchmarking

#### Embedding quality

To evaluate UNAGI’s performance in learning latent embeddings from single-cell data, we compared it with several other methods by running individual methods ten times with different random seeds. These included VAE-based dimensionality reduction techniques such as scVI^19^ and scGEN^33^, the foundation models, scGPT^31^, Geneformer^144^ and Universal Cell Embeddings (UCE)^173^, other deep-learning methods using GAN or GCN, including GraphSCC^22^, scGGAN^20^ and scGNN^21^, as well as standard single-cell analysis pipelines such as Seurat and SCANPY. To show the necessity of using rigorous data cleaning and normalization strategies to preprocess the complex single-cell dataset (for example, the IPF dataset), we kept the top 2,000 highly variable genes and ran standard SCANPY pipeline to analyse the raw IPF dataset. We adopted bio-conservation metrics from Luecken et al.^145^, including ARI, NMI, graph cell-type local inverse Simpson’s index (graph cLISI), silhouette score, cell-type ASW, isolated label F1 and isolated label silhouette score, SCIB bio-conservation overall score^145^ along with Davies–Bouldin index (DBI)^174^ and label score^175^ to evaluate the benchmarking methods. The label score assesses the consistency of cell types in the cell neighbourhoods. The DBI measures the average similarity ratios between clusters. The silhouette score evaluates the cohesion and separation of clusters in the embedding space, and cell-type silhouette score assesses the cohesion and separation of cell populations in the embedding space. Isolated cell-type F1 score describes how well isolated cell types are distinguished from other cell types. ARI and NMI calculate the coherence between the cell populations identified by clustering methods and ground truth cell types. Graph cLISI measures the preservation of cell populations across datasets and is a critical metric for assessing the robustness and generalizability of cell embeddings. The SCIB overall bio-conservation score is the average of ARI, NMI, cell-type ASW, isolated cell-type F1, isolated cell-type ASW and graph cLISI. In our IPF dataset, we provided handcraft cell-type annotations derived by recursively annotating and refining cell types on individual samples. Thus, the results from the standard Seurat pipeline cannot serve as a perfect proxy for the ground truth scores to evaluate the clustering results from other methods. Because UCE is designed specifically for the zero-shot usage, we only tested it in zero-shot mode (Supplementary Fig. 12 and Supplementary Note 11), while scGPT and Geneformer were evaluated in both fine-tuned and zero-shot settings. See benchmarking method details in Supplementary Note 12.

#### Computing efficiency

To evaluate the computational efficiency of processing large-scale single-cell data, we analysed the memory requirements and running time of various deep-learning methods, including UNAGI, scGEN, scGPT, scVI, GraphSCC, scGGAN and scGNN. The experiments were conducted on a workstation equipped with an RTX 4090, AMD Ryzen Threadripper Pro 5965wx and 256GB RAM. We downsampled the IPF dataset (231,477 cells with 2,484 genes) into subsets of 23,000 cells (~10%), 46,000 cells (~20%), 58,000 cells (~25%), 116,000 cells (~50%) and 173,000 cells (~75%) to assess the efficiency of each method on different scales. After data cleaning and normalization, we ran each method, recording the total running time and memory usage for preprocessing, model training, clustering and UMAP generation. We also compared the inference efficiency of all benchmarking methods using the IPF dataset with 231,477 cells and 2,484 genes.

#### Disease-associated pathway identification

We benchmarked against existing methods to identify disease-associated pathways by using the embeddings generated by other methods to build the dynamic graphs and run iDREM to reconstruct the temporal regulatory networks for individual cell tracks (the trajectories represent the change of cellular states associated with a certain cell population during disease progression, from healthy to the end disease grade). We used the most increasing set of genes from the iDREM results of fibroblast alveolar tracks to perform pathway enrichment analysis to identify disease-associated pathways. We used the cell embeddings generated from the embedding quality benchmarking experiments to build the dynamics graphs and perform the pathway identification experiments using Toppgene^176^. We used the –log_10_(FDR) to represent the significance of identified pathways from the Toppgene.

#### Disease marker identification

In a manner similar to the identification of disease-associated pathways, we derived the temporal dynamics graph using identical experiment settings. We then proceeded to identify the dynamic markers in fibroblast cell tracks by using the method described in the ‘Dynamic and hierarchical static markers discovery’ section. To evaluate the agreement between the disease markers and the proteomics markers, we performed the hypergeometric test to evaluate the overlapping of dynamic markers and proteomics markers described in the section ‘Verify UNAGI biomarkers by proteomics data’ of Methods.

#### In silico drug screening simulation

We benchmarked UNAGI against scGPT^31^, scVI^19^, Geneformer^144^ and scGEN^33^, and directly calculated the shifts in the gene space (denoted as ‘Original’) on the in silico drug screening task. We excluded UCE from this drug screening benchmarking owing to its high computational complexity (Extended Data Fig. 4c). We separated the dataset into healthy control and IPF disease groups to train the scGEN to learn the transition between IPF and healthy cells. For scVI, we used tissue fibrosis grades as the batch label to learn cell embeddings. scGPT was fine-tuned on the IPF data to generate the cell embeddings. To directly calculate the shifts in the original gene space (‘Original’), we did not perform dimensionality reduction. Using the same strategy as UNAGI, we modified the gene expression values as the input to send to these methods. Deep-learning-based methods calculate the in silico perturbation score in the cell embedding. For the ‘Original’ method, we used the (1-Corr_Spearman_) as the distance metric to calculate the perturbation score.

To run the benchmarking experiments, we generated simulated data by shuffling gene expression profiles and implanting drug effects as ground truth by manually altering the expression of their target genes (see Supplementary Note 13 for details). For UNAGI, scGPT, Geneformer, scVI and scGEN, we pre-trained the model (fine-tuned scGPT and Geneformer) on the original dataset before executing simulation tasks.

The modified fibroblast cells were then sent to the fine-tuned deep-learning models to obtain the perturbed cell embeddings. We established the random background score distribution by performing in silico drug perturbations 2,000 times on the original dataset by randomly sampling *n*_*p*_ genes, where *n*_*p*_ is sampled from the probability based on the number of target genes for the drugs that we used. The FDR of simulation perturbation scores was calculated against the random score background distribution. We used FDR < 0.05 as the cut-off to determine whether the in silico perturbation could identify the simulated drug or not. Using this strategy, we can evaluate the model as a classifier in a binary classification task, specifically in determining the effectiveness of simulated drugs. The model’s performance in drug screening was assessed using the AUROC and the AUPRC metrics, as implemented in scikit-learn^177^. In this binary classification setting, both the ROC and PRC curves have only one classification threshold of 0.5.

#### Predicting post-treatment gene expression changes

We benchmarked UNAGI with scGPT, scGEN and scVI, and directly changed the gene expression (‘Original’) to predict the gene expression after treatments using the snRNA-seq PCLS dataset. We excluded Geneformer from post-treatment gene expression prediction benchmarking owing to its BERT-based structure. This dataset consists of four groups of data, control, fibrosis cells, fibrosis cells after nifedipine treatment and fibrosis cells after nintedanib treatment. First, we trained UNAGI, scVI and scGEN on the control and fibrosis group of data. UNAGI treated them as the control and disease grades, scGEN considered them as two states, and scVI treated control and fibrosis groups as two batches. Second, we identified the top 10 DEG markers after ex vivo nintedanib and nifedipine treatments on fibrosis cells (treatment markers). We modified the expression of the top 10 nifedipine or nintedanib treatment markers and sent the modified cells into the model to predict cells after treatments. For the ‘Original’ method, we directly modified the top nifedipine or nintedanib treatment markers in the gene space. For scGPT, the performance of scGPT in embedding cells increases largely after fine-tuning (Supplementary Note 14). Thus, we fine-tuned the model on the control and fibrosis cells and performed supervised perturbation prediction using fibrosis cells and treatment cells (see details in Supplementary Note 15). During the testing process, we investigated how the model can predict the perturbation using only the top 10 treatment markers, like other methods. We excluded GEARS in the benchmarking, because it fell short compared with scGPT and it lacks the ability to produce cell embeddings for in silico drug screening^31^. Then, we calculated the Pearson correlation of the changes from fibrosis cells to cells generated by models and cells after ex vivo treatments. In addition, we analysed the relationship between top-weighted genes and the treatment markers. We used the Monte Carlo sampling strategy to determine the percentile of the ranking for a random set of 100 genes. We also performed the pathway enrichment analysis using the Toppgene^176^.

### Ablation study

To investigate the contribution of individual parts to UNAGI’s performance, we conducted ablation studies on cell embedding, cell generation and the identification of disease markers and disease-associated pathways tasks using both IPF and the COVID-19 datasets. We compared UNAGI with UNAGI w.o. GCN and GAN, UNAGI w.o. GCN and UNAGI w.o. GAN to analyse the impacts of individual deep-learning components. We used ZINB distribution in UNAGI (UNAGI w. ZINB) to process the IPF dataset to evaluate the effectiveness of ZILN distribution. We also conducted experiments to compare scVI and scVI-ZILN in the IPF dataset to show that ZILN distribution can also improve other methods. The UNAGI w.o. iteration strategy directly trains the UNAGI model to convergence without the iterative training strategy. The same as in the benchmarking experiments, we ran individual methods ten times with different random seeds.

### Reporting summary

Further information on research design is available in the Nature Portfolio Reporting Summary linked to this article.

## Supplementary information

Supplementary Information Supplementary Figs. 1–22 and Notes 1–16.

Reporting Summary

Supplementary Tables 1–5 Dynamic markers, proteomics data analysis, pathway perturbation results, drug perturbation results, overview of the applicability and performance of selected methods across various tasks.

## Data Availability

IPF snRNA-seq (GSE286182)^178^ can be publicly accessible at https://www.ncbi.nlm.nih.gov/geo/query/acc.cgi?acc=GSE286182. The COVID-19 dataset (COVID-19 PBMC Ncl-Cambridge-UCL) is currently available from the COVID-19 Cell Atlas at https://covid19cellatlas.org/. The proteomics data are publicly available via MassIVE with project identifier MSV000093129 (or Zenodo repository at 10.5281/zenodo.15597088 (ref. ^179^)). The preprocessed PCLS data are available at our GitHub repository (https://github.com/mcgilldinglab/UNAGI). Hippie database^156^ can be publicly accessed at https://cbdm-01.zdv.uni-mainz.de/~mschaefer/hippie/download.php. STRINGDB^157^ is publicly available at https://string-db.org/. REACTOME^158^ can be accessed at https://reactome.org/, MatrisomeDB^159^ is available at https://matrisomedb.org/, and KEGG^160^ can be found at https://www.genome.jp/kegg/pathway.html. The Connectivity MAP (CMAP)^35^ database is publicly available at https://clue.io/data/CMap2020#LINCS2020.

## Code availability

The UNAGI software package and source code are available at our GitHub repository (https://github.com/mcgilldinglab/UNAGI)^180^. The results and downstream analysis are available at our web server (http://dinglab.rimuhc.ca/unagi). All preprocessed.h5ad files used in this study are also available in the same GitHub repository. The software and third-party packages used in this work, including PyTorch (version 2.0.0), SCANPY (version 1.9.5) and Pandas (version 2.1.0), are listed in Supplementary Note 16.
