# Supplementary material for: A deep generative model for deciphering cellular dynamics and in silico drug discovery in complex diseases
Source: Nat Biomed Eng. 2025 Jun 20;9(12):2155–80. doi: 10.1038/s41551-025-01423-7 (PMC12705450; doi:10.1038/s41551-025-01423-7)
Supplement: Supplementary file 1 — Supplementary Figs. 1–22 and Notes 1–16. [file 41551_2025_1423_MOESM1_ESM.pdf]

nature biomedical engineering

Supplementary information

https://doi.org/10.1038/s41551-025-01423-7

A deep generative model for deciphering cellular dynamics and in silico drug discovery in complex diseases

In the format provided by the authors and unedited

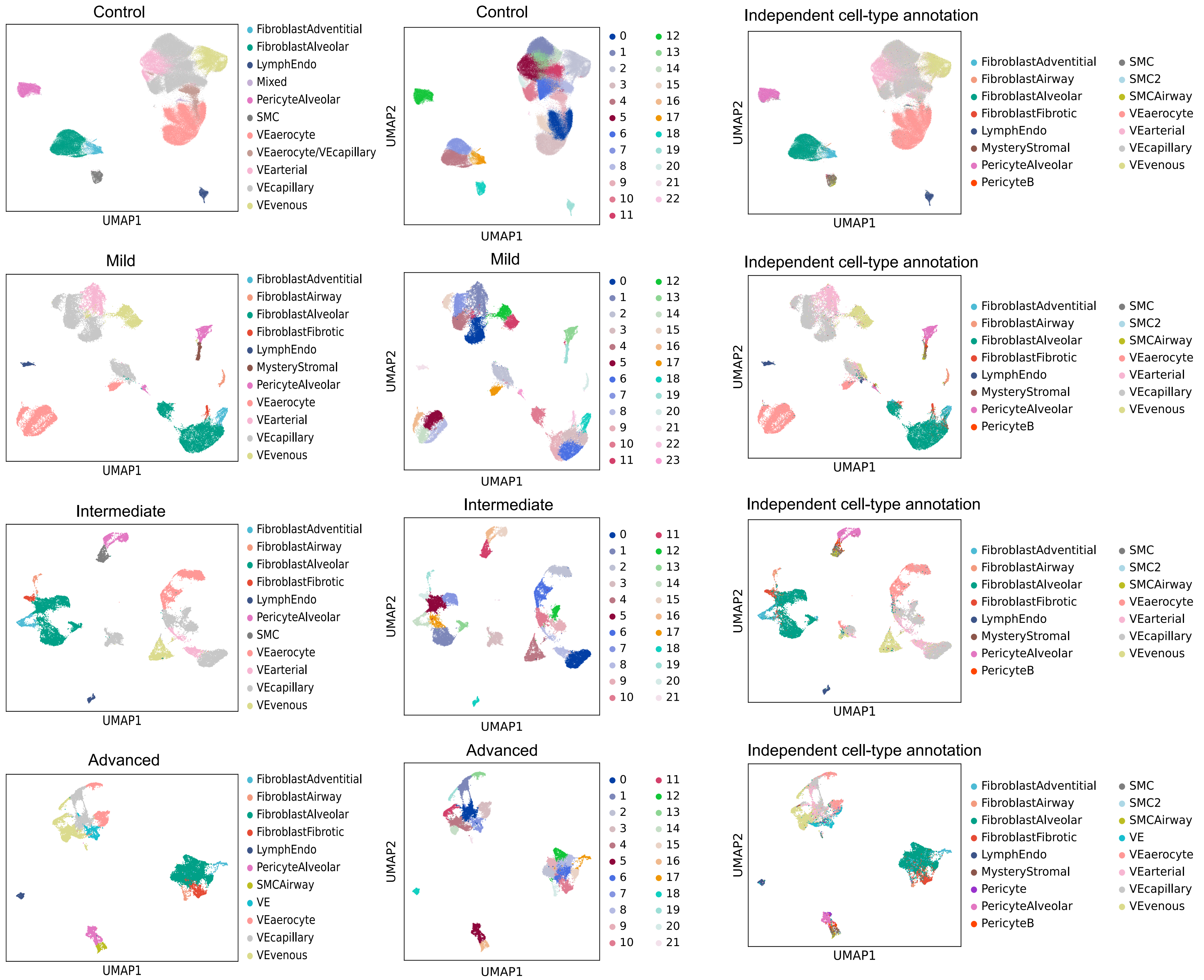

Supplementary Fig. 1 UMAP Visualization of cell embeddings and clusterings from UNAGI and the independent manual cell-type annotations by Seurat. This figure depicts stroll cells across various tissue fibrosis grades in IPF. Each point represents a cell. The figure is organized into three columns: the first column displays cell types annotated based on clusters, the second column shows Leiden cluster IDs, and the third column shows independent manual cell-type annotations as labelled by Seurat.

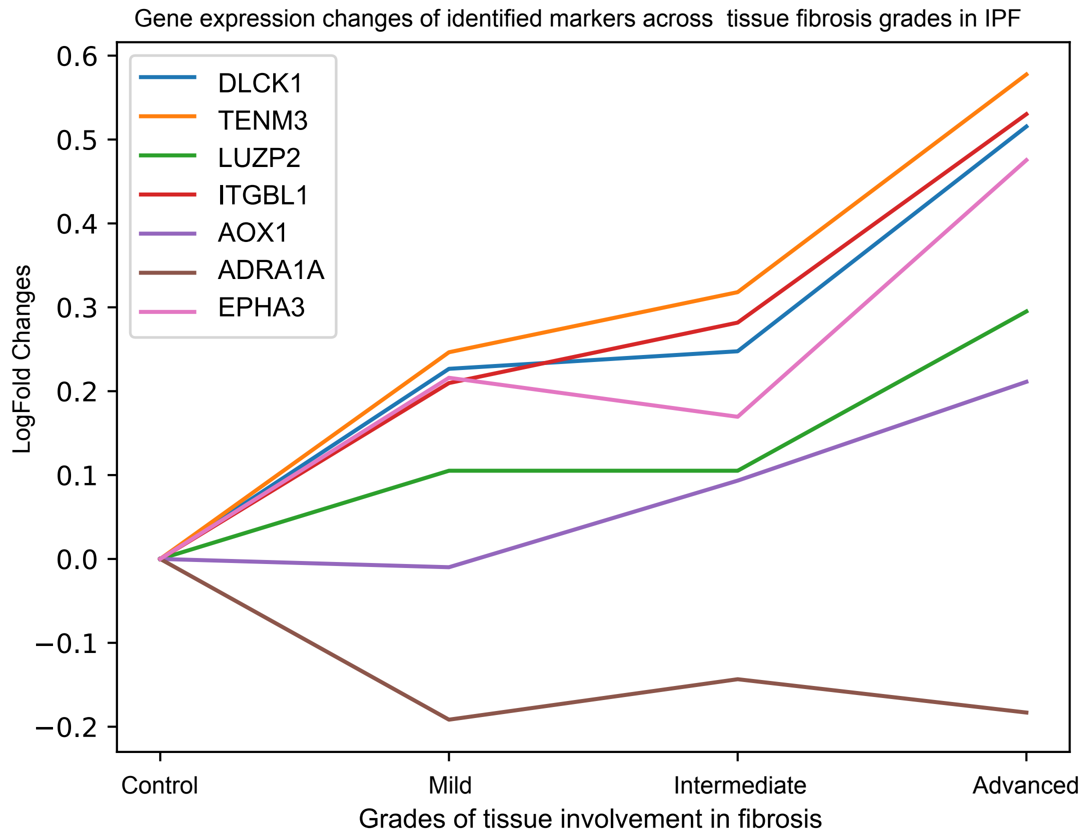

Supplementary Fig 2. The Line chart displays the average expression changes of some identified IPF markers across different grades of tissue involvement in fibrosis.

a

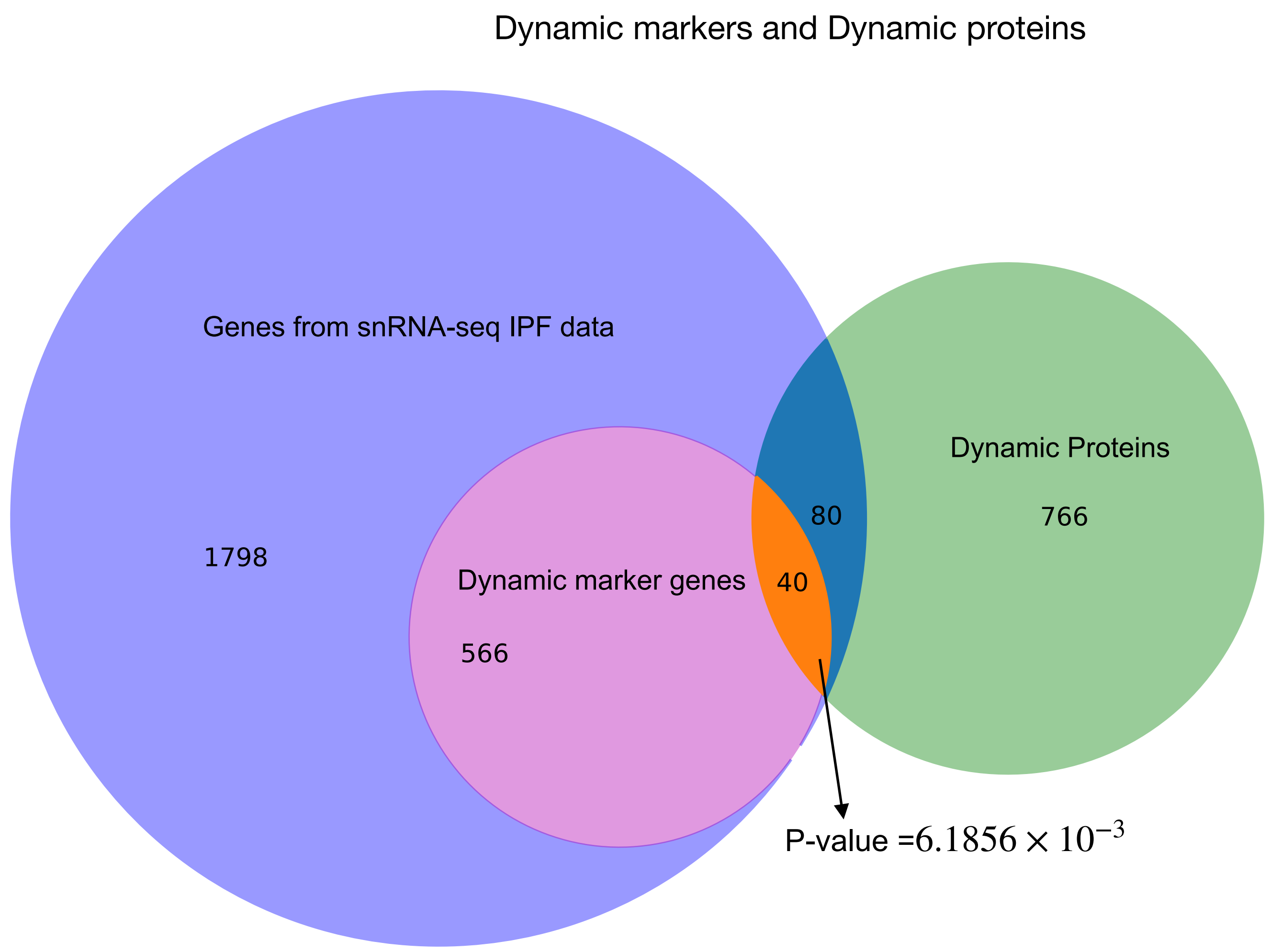

b

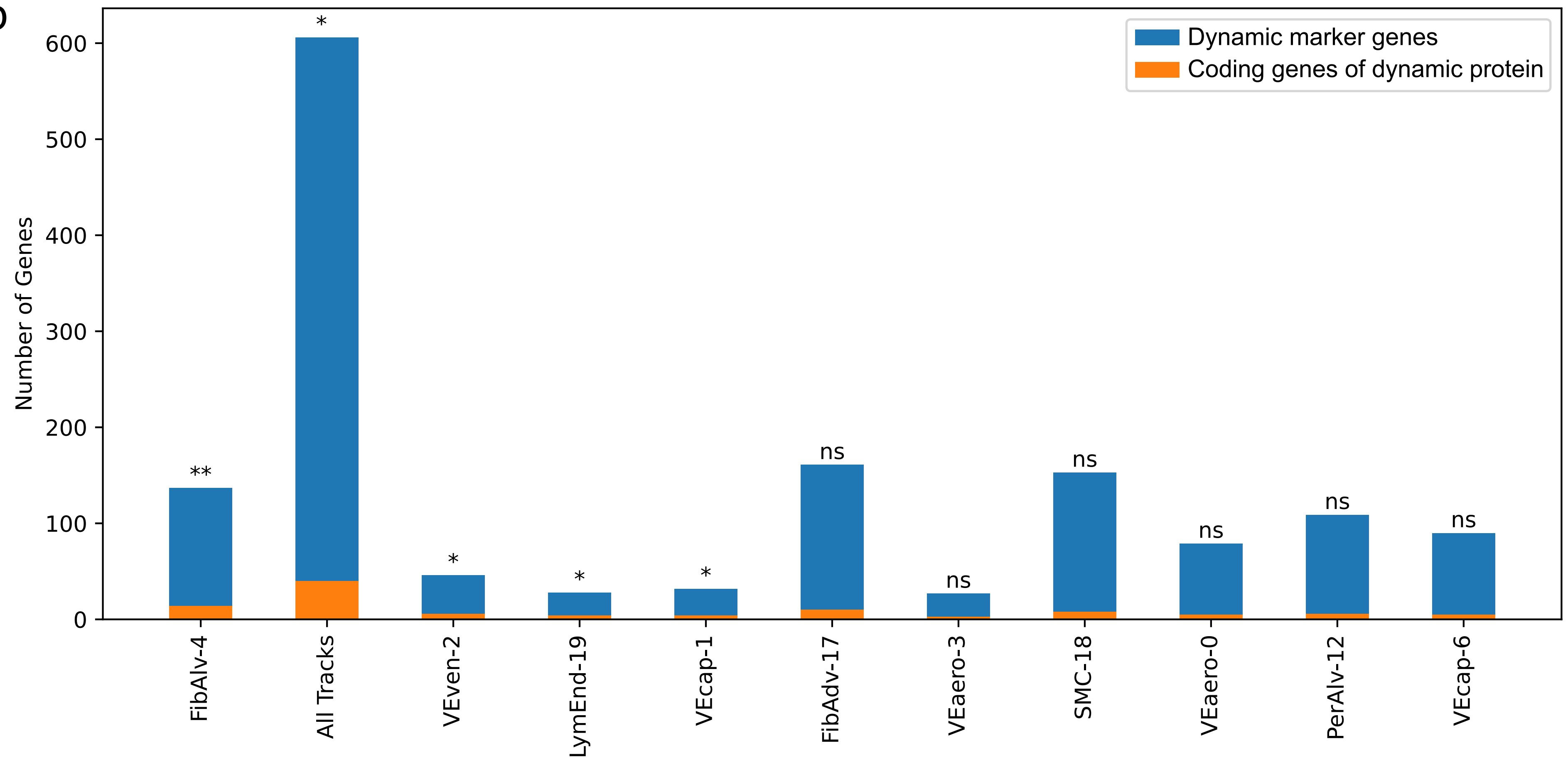

Supplementary Fig. 3 Dynamic gene markers align with dynamic proteins a, The Venn diagram illustrates the intersection between genes from the IPF snRNA-seq dataset, dynamic marker genes identified within the same dataset, and dynamic proteins identified in proteomics data. 40 dynamic proteins, which have corresponding protein-coding genes in the snRNA-seq dataset, are highlighted in orange. A hypergeometric test $p(k, M, n, N)$ is utilized to assess the overlapping ratio between dynamic marker genes and dynamic proteins, with 'N' representing the total of 2484 snRNA-seq genes, 'M' denoting 120 dynamic proteins overlapping with the snRNA-seq dataset, 'n' being 606 dynamic marker genes, and 'k' as the 40 dynamic genes that encode dynamic proteins. **b**, The bar displays the intersection of dynamic marker genes and dynamic protein coding genes in the snRNA-seq dataset. The one-sided hypergeometric test (referenced in panel **a**) calculates the P-value for the overlapping ratio of dynamic marker genes and dynamic proteins across individual tracks, providing a statistical assessment of their correlation. (*:0.005<P-value<0.05,**:0.0005<P-value<0.005 ns: not significant)

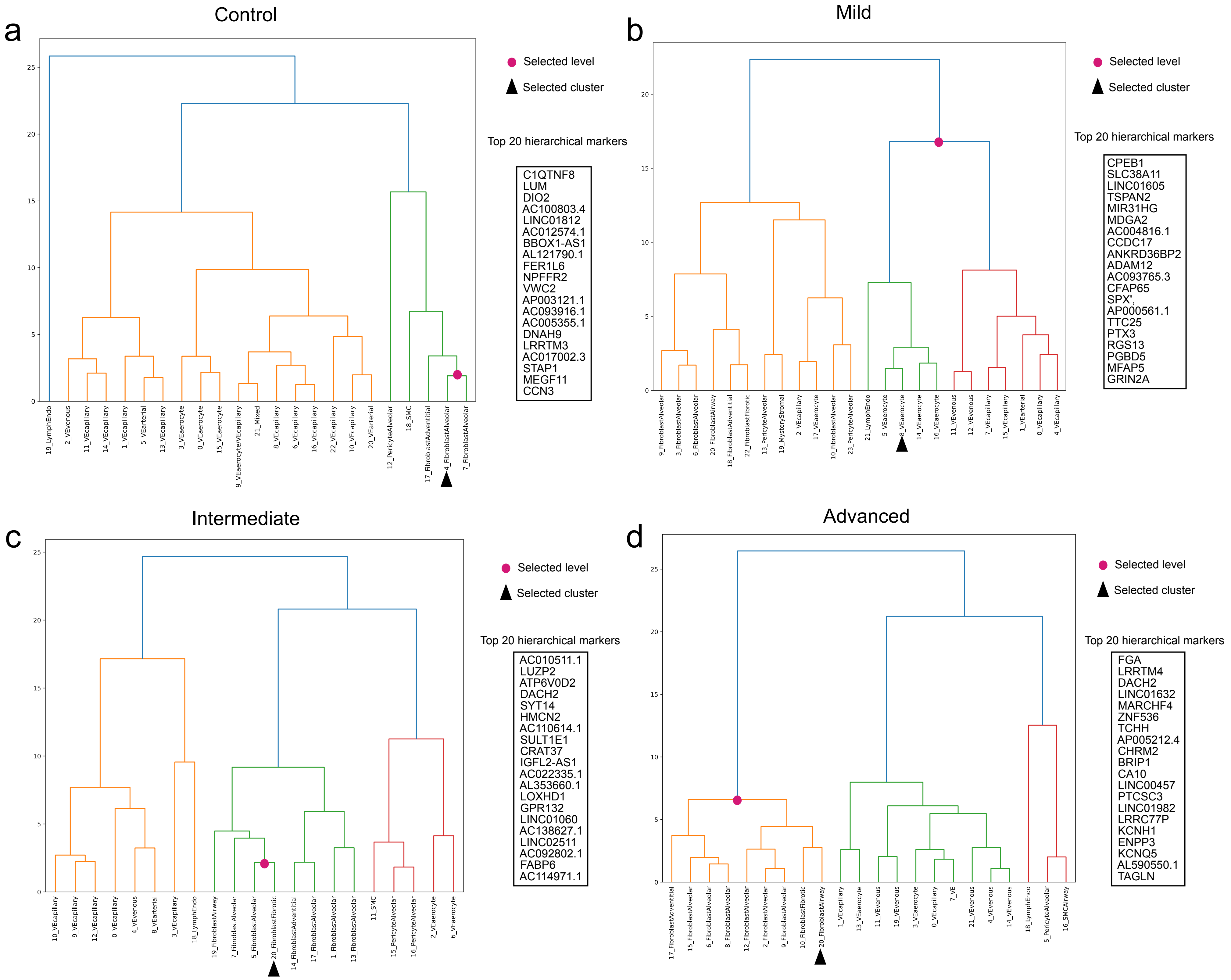

Supplementary Fig. 4 Dendrograms of the hierarchical static markers across tissue fibrosis grades. This figure displays the results of hierarchical static markers for different tissue fibrosis grades in IPF, with each cluster clearly labeled by its cluster ID and cell type. **a**, the top 20 hierarchical static markers of the cluster 4, level 5 at the Control. **b**, the top 20 hierarchical static markers of the cluster 8, level 1 at the Mild fibrosis. **c**, the top 20 hierarchical static markers of the cluster 20, level 5 at the Intermediate. **d**, the top 20 hierarchical static markers of the cluster 20, level 1 at the Advanced. The hierarchical static markers associated with each tissue fibrosis grade's dendrogram are available for detailed exploration and analysis through our web visualization application, accessible at [http://dinglab.rimuhc.ca/unagi/].

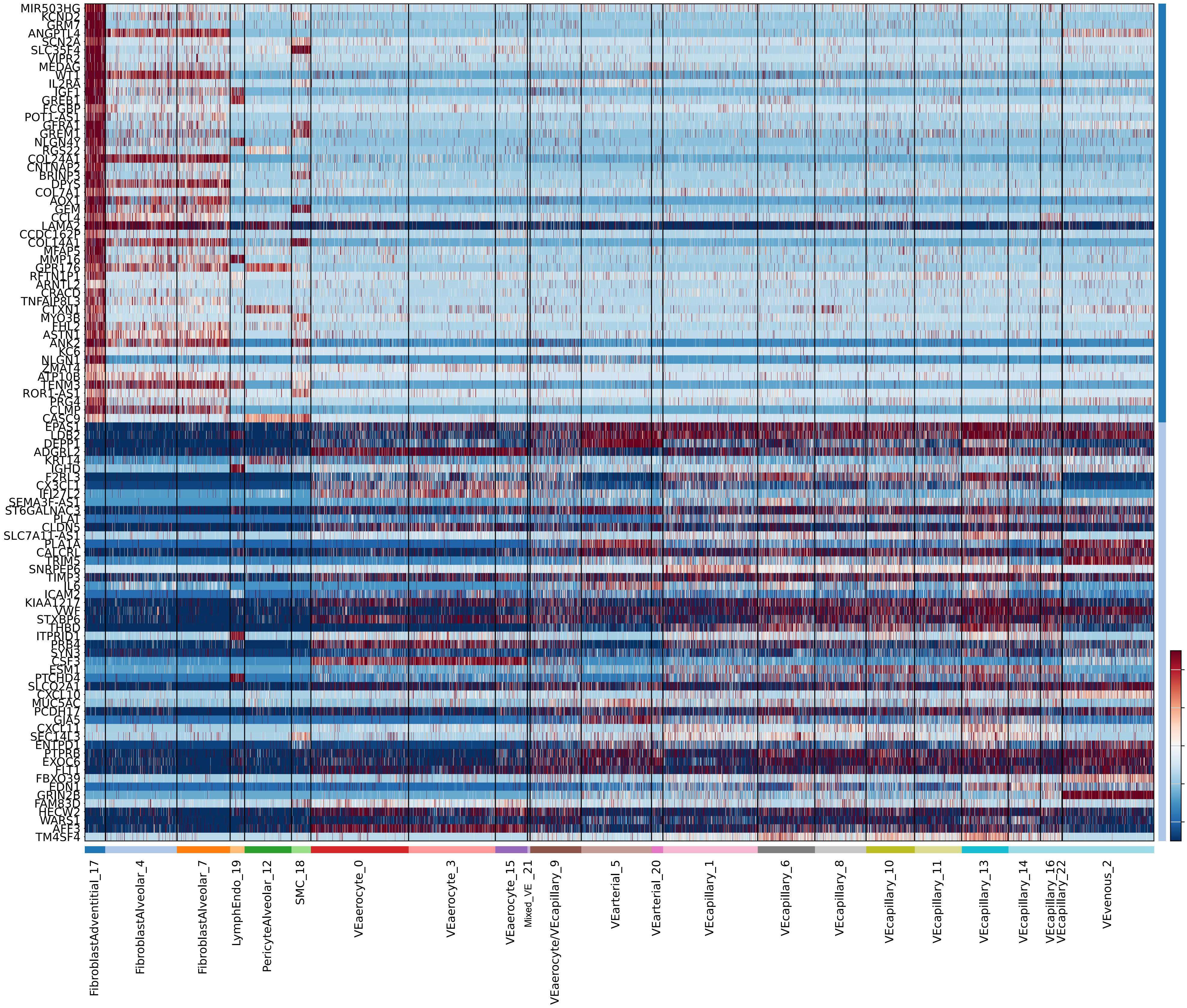

Supplementary Fig. 5 Heatmap of level 0 hierarchical static markers in the Fibroblast Adventitial cluster at the control. This heatmap illustrates the expression of the top 25 positive and negative level 0 hierarchical static markers for the Fibroblast Adventitial cluster from the control, with all values Z-score normalized for comparison. Each row in the heatmap represents a specific gene marker, while each column corresponds to an individual cell. The X-axis is annotated with labels indicating the type of cell population and the associated Leiden cluster ID. The heatmap uses a color gradient to depict gene expression levels, with high expression shown in red and low expression in blue, providing a visual representation of the expression patterns of these key markers.

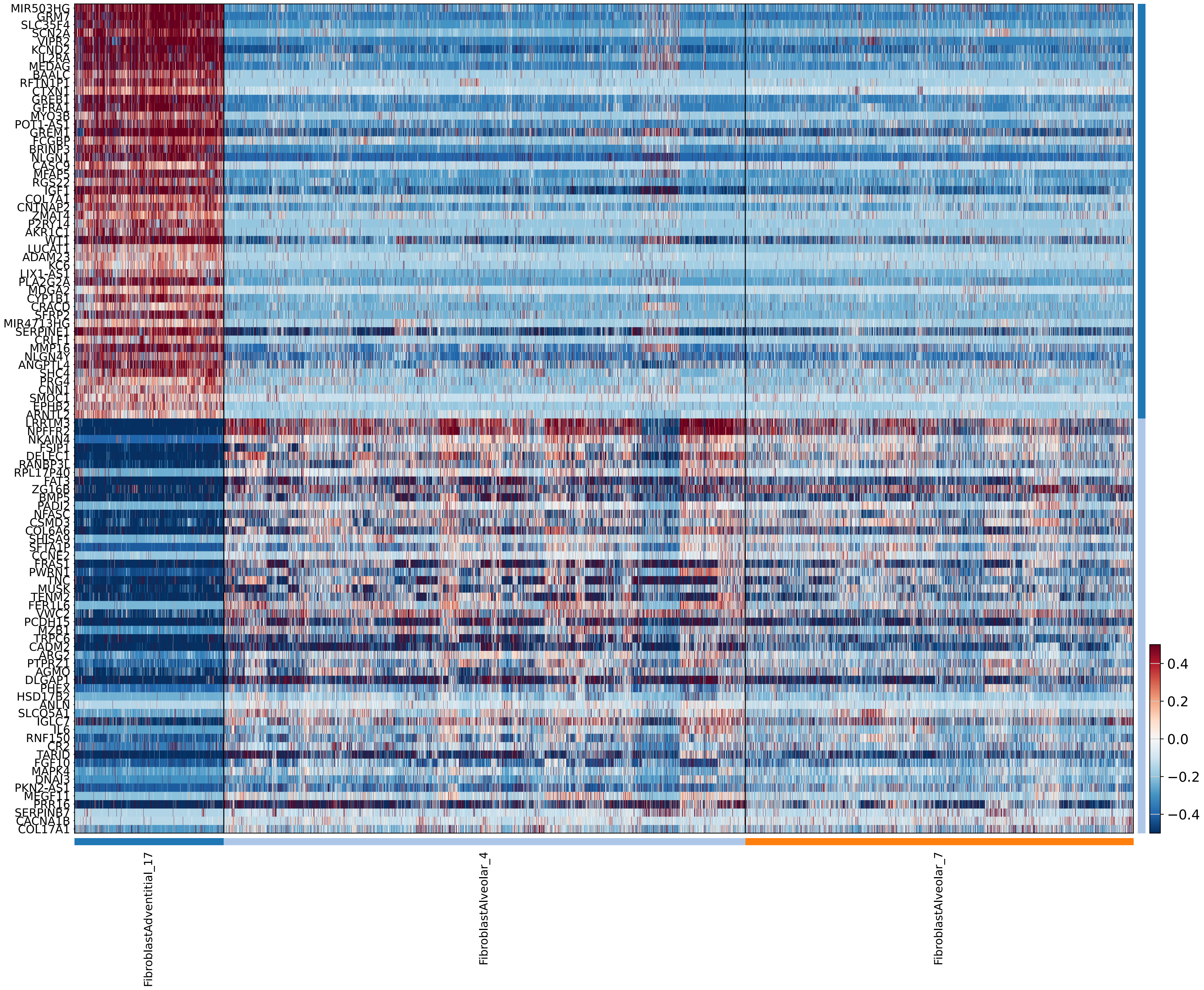

Supplementary Fig. 6 Heatmap of level 4 hierarchical static markers in the Fibroblast Adventitial cluster at the control. This heatmap illustrates the expression of the top 25 positive and negative level 4 hierarchical static markers for the Fibroblast Adventitial cluster from the control, with all values Z-score normalized for comparison. Each row in the heatmap represents a specific gene marker, while each column corresponds to an individual cell. The X-axis is annotated with labels indicating the type of cell population and the associated Leiden cluster ID. The heatmap uses a color gradient to depict gene expression levels, with high expression shown in red and low expression in blue, providing a visual representation of the expression patterns of these key markers.

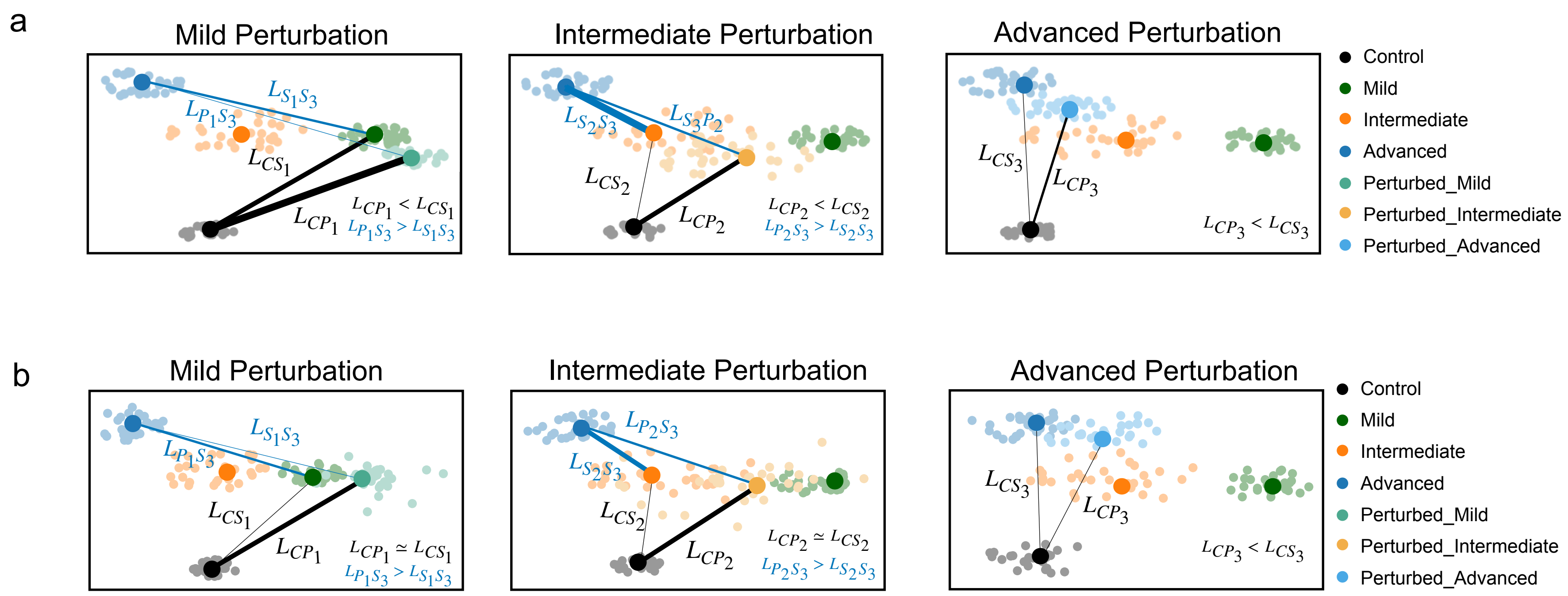

Supplementary Fig. 7 PCA plots visualization of in-silico perturbation effects. a, PCA plots of latent space Z of in-silico ECM organization pathway perturbation effects and dots represent cells from distinct tissue fibrosis grades. Lines connected to two nodes are the PAGA connectivity score between two clusters, where the width of a line is proportional to the strength of the score, and the length of the line can represent the distance between the UNAGI embeddings of the two connected clusters. (e.g., Line connecting Control and Perturbed Mild (L_{CP_1}). **b**, PCA plots of Nintedanib perturbation effectiveness.

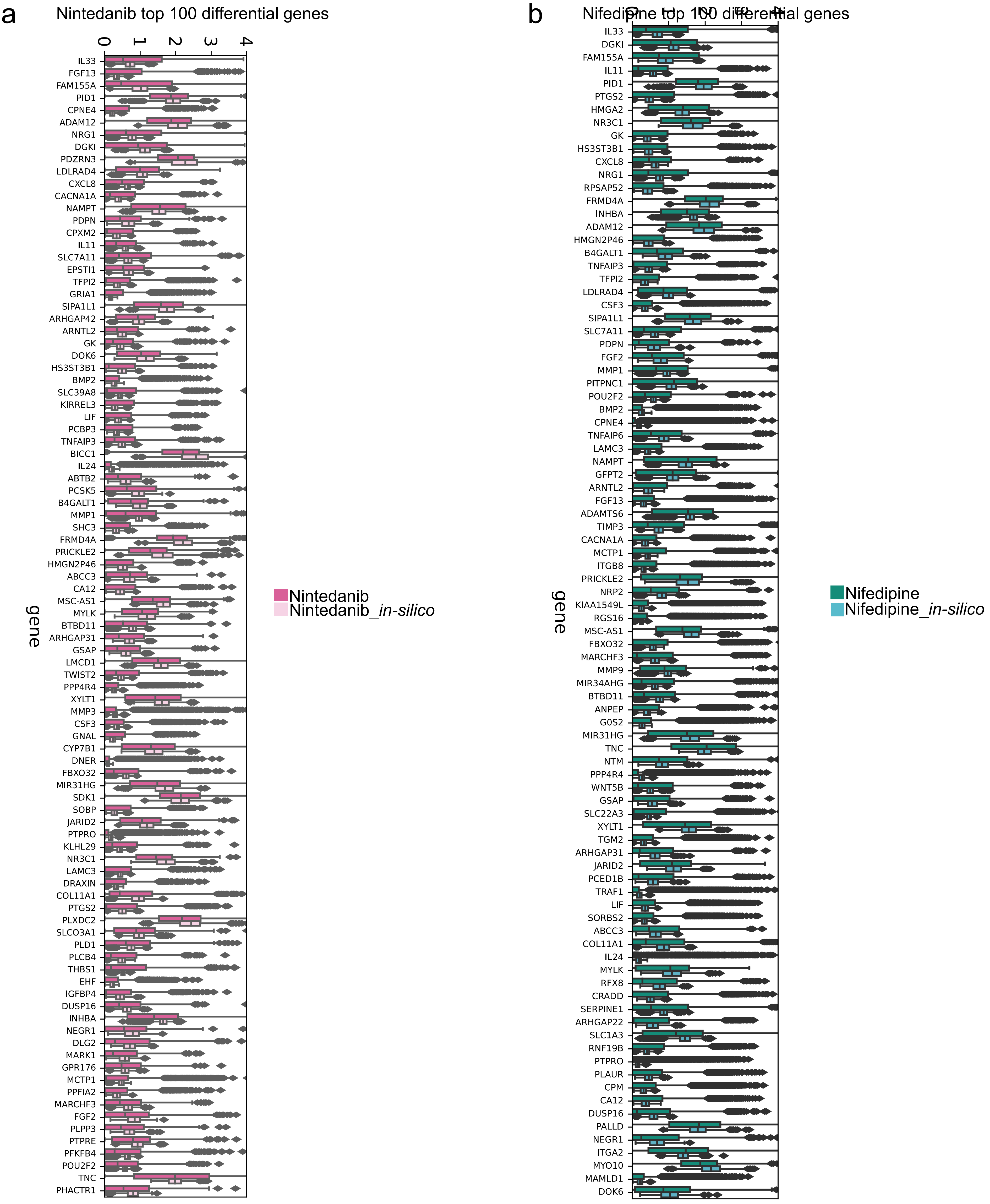

Supplementary Fig. 8 Compare the in-silico and real perturbation outcomes on top differentially expressed genes a, Box plots display the expression of the top 100 differentially expressed genes from Nintedanib treatments compared to fibrotic conditions, alongside their corresponding expression from in-silico perturbations. These top genes are ranked based on log-fold changes. **b**, Similarly, box plots illustrate the expression of the top 100 differentially expressed genes from actual Nifedipine treatment compared to fibrotic conditions, contrasted with in-silico perturbation outcomes. The genes are also arranged by log fold changes. The number of cells (n) of Nintedanib, Nintedanib_in-silico, Nifedipine, and Nifedipine_in-silico are 2264, 4918, 8024, and 4918. The boxes represent the interquartile ranges (IQRs), and the solid lines indicate the medians. The whiskers extend to points within 1.5 IQRs of the lower and upper quartiles.

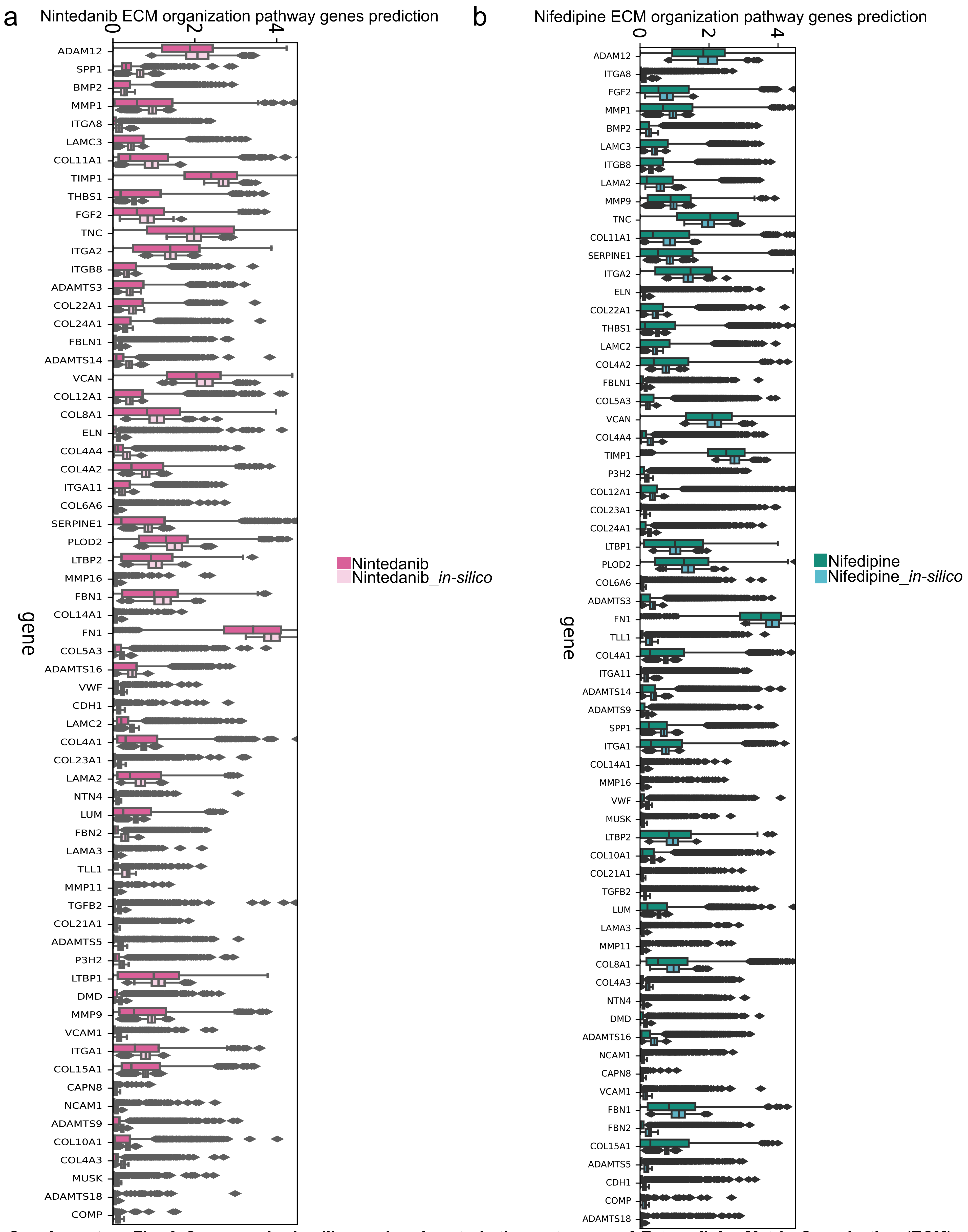

Supplementary Fig. 9 Compare the in-silico and real perturbation outcomes of Extracellular Matrix Organization (ECM) target genes a, Box plots display the expression of ECM organization target genes from actual Nintedanib treatments alongside their corresponding expression from in-silico perturbations. These genes are ranked based on their log-fold changes. **b**, Similarly, box plots illustrate the expression of the ECM organization target genes from actual Nifedipine treatments against in-silico perturbation outcomes. The genes are also arranged by log fold changes. The number cells (n) of Nintedanib, Nintedanib_in-silico, Nifedipine, and Nifedipine_in-silico are 2264, 4918, 8024, and 4918. The boxes represent the interquartile ranges (IQRs), and the solid lines indicate the medians. The whiskers extend to points within 1.5 IQRs of the lower and upper quartiles.

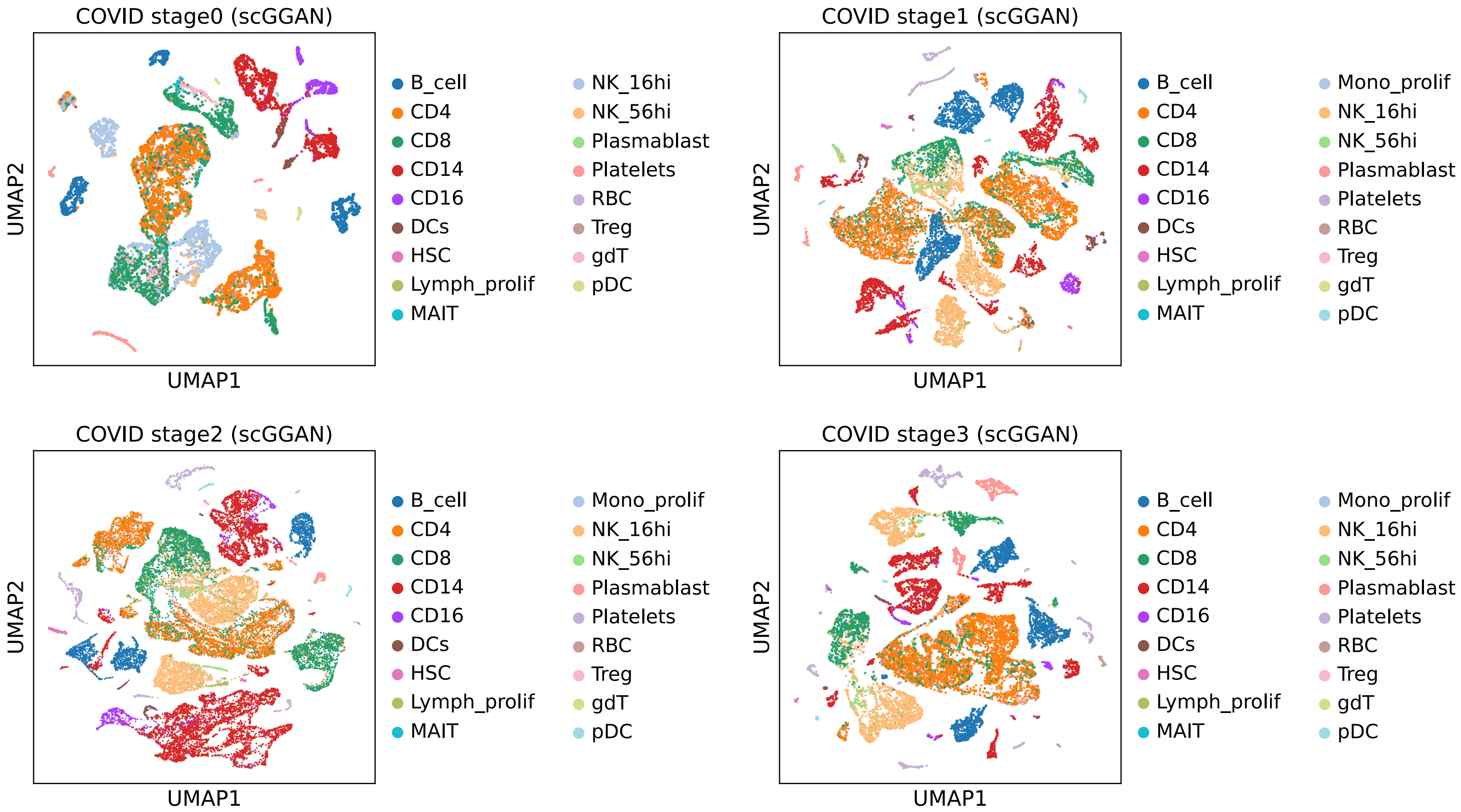

Supplementary Fig. 10 The UMAP visualization of scGGAN embeddings across individual COVID-19 stages. Cell populations with the same type were randomly distributed in the embedding space.

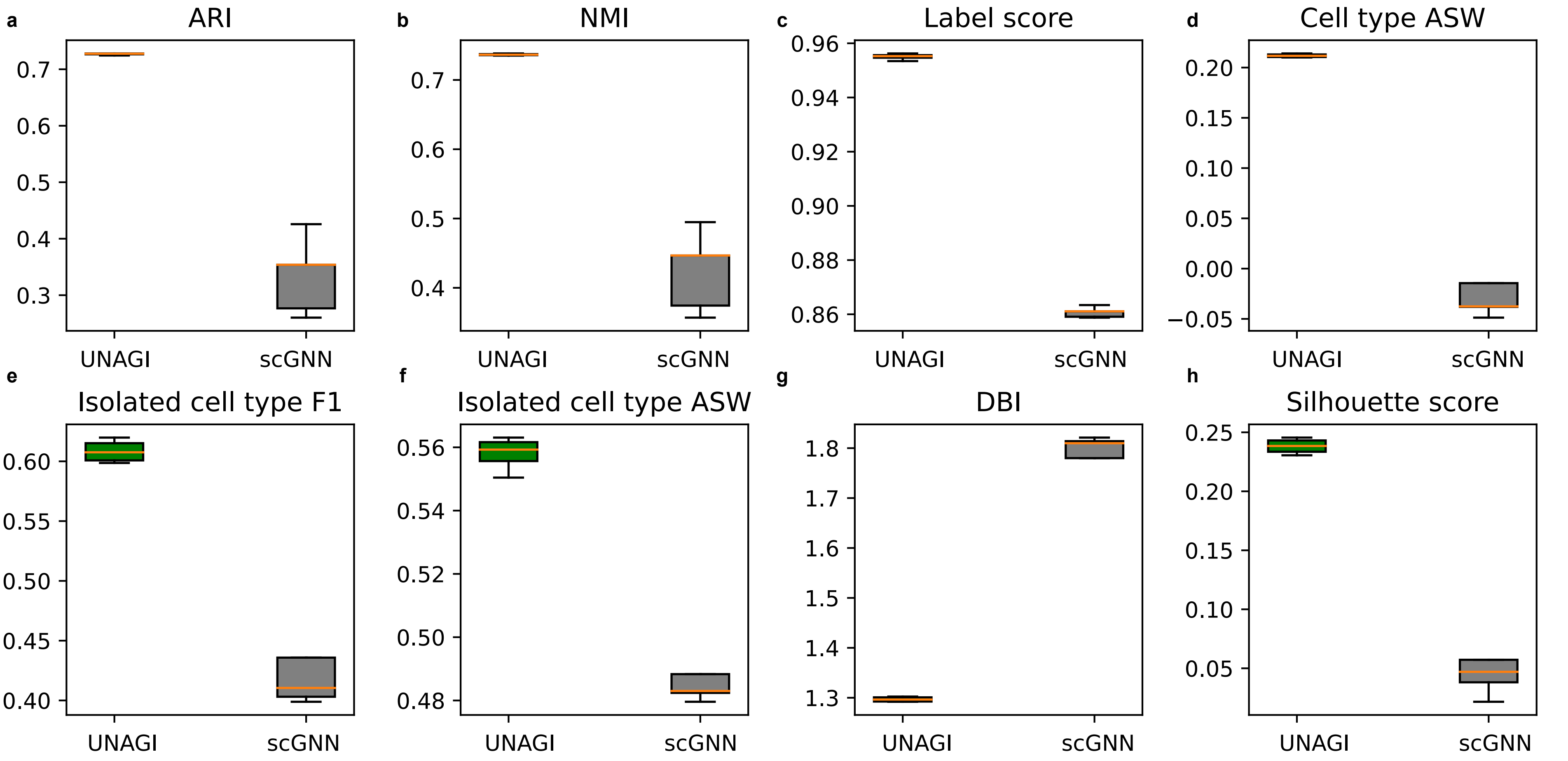

Supplementary fig. 11 The benchmarking of embedding quality of UNAGI and scGNN using 25% of the IPF dataset. a, Adjusted Rand Index (ARI). **b**, Normalized Mutual Information (NMI). **c,** Label score. d, Cell type ASW. **e**, Isolated cell type F1 score. **f**, Isolated cell type ASW. **g**, Davis-Bouldin index (DBI); a lower DBI signifies better clustering results. **h**, Silhouette score. The scores **d**, **e**, and **f** indicate the cell-type informativeness within the embedding space, where a higher score reflects a stronger ability to retain cell identity information. The scores of **g** and **h** focus on intra-cluster similarity, distinctness, and compactness. These results showed that UNAGI still clearly outperformed scGNN under the down-sampled experiment setting. The experiments in panels a-h run with different seeds (n=10). The boxes represent the interquartile ranges (IQRs), and the solid lines indicate the medians. The whiskers extend to points within 1.5 IQRs of the lower and upper quartiles.

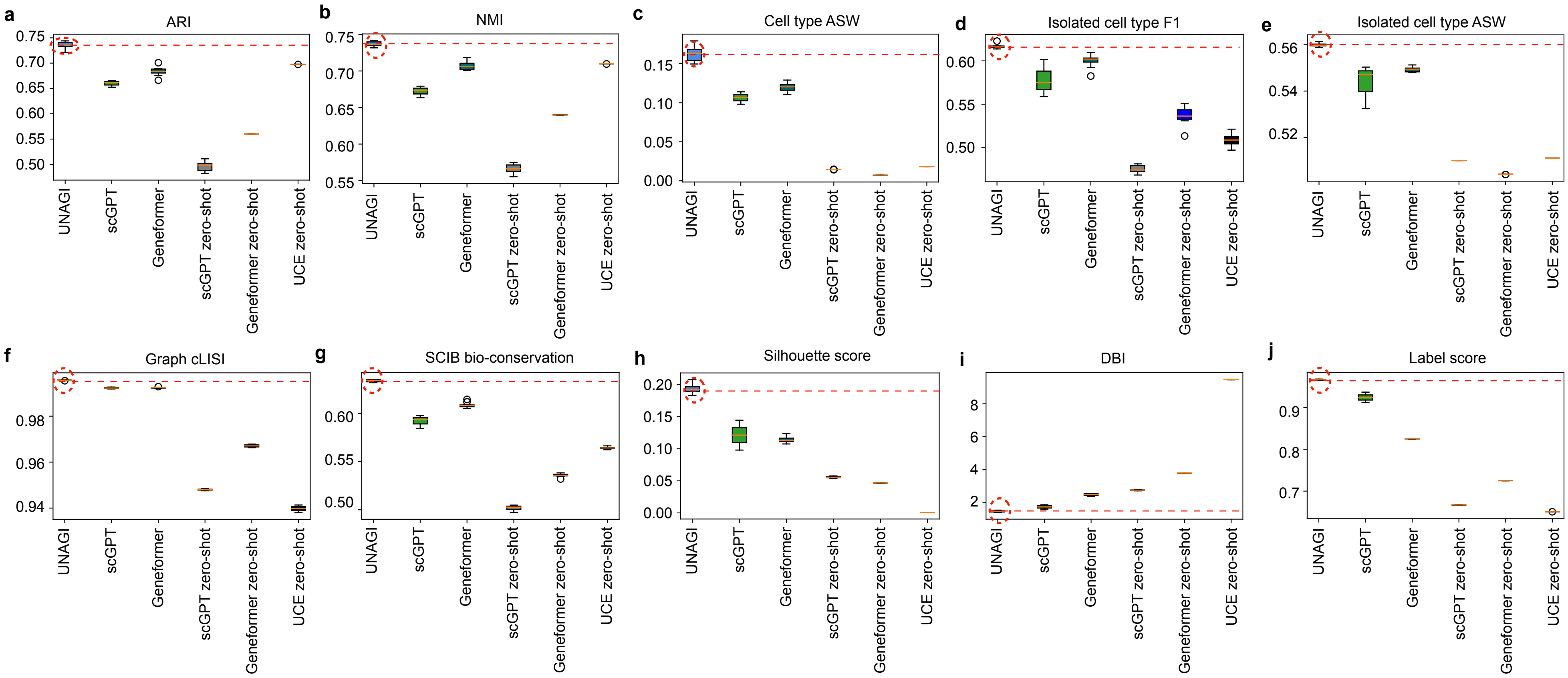

Supplementary Fig. 12, Benchmarking UNAGI against foundation models before and after fine-tuning using the IPF dataset. a, Adjusted Rand Index (ARI). **b**, Normalized Mutual Information (NMI). **c**, Cell type ASW. **d**, Isolated cell type F1 score. **e**, Isolated cell type ASW. **f**, Graph cLISI score. **g**, SCIB overall bio-conservation score. **h**, Silhouette score. **i**, Davis-Bouldin index (DBI); a lower DBI signifies better clustering. **j**, Label score. From left to right, the ablation models are UNAGI, scGPT (fine-tuned), Geneformer (fine-tuned), scGPT (zero-shot), Geneformer (zero-shot), and UCE (zero-shot). Scores **a** and **b** illustrate the effectiveness of the learned cell embeddings for clustering tasks. The scores **c**, **d**, and **e** indicate the cell-type informativeness within the embedding space, where a higher score reflects a stronger ability to retain cell identity information. The score **f** represents the similarity of cells within a KNN graph. SCIB bio-conservation overall score **g** is the summary over score **a-f**. of The scores of **h** and **i** focus on intra-cluster similarity, distinctness, and compactness. Score **j** represents the similarity between cells' neighborhoods in the embedding space. The experiments in panels a-j run with different seeds (n=10). The boxes represent the interquartile ranges (IQRs), and the solid lines indicate the medians. The whiskers extend to points within 1.5 IQRs of the lower and upper quartiles.

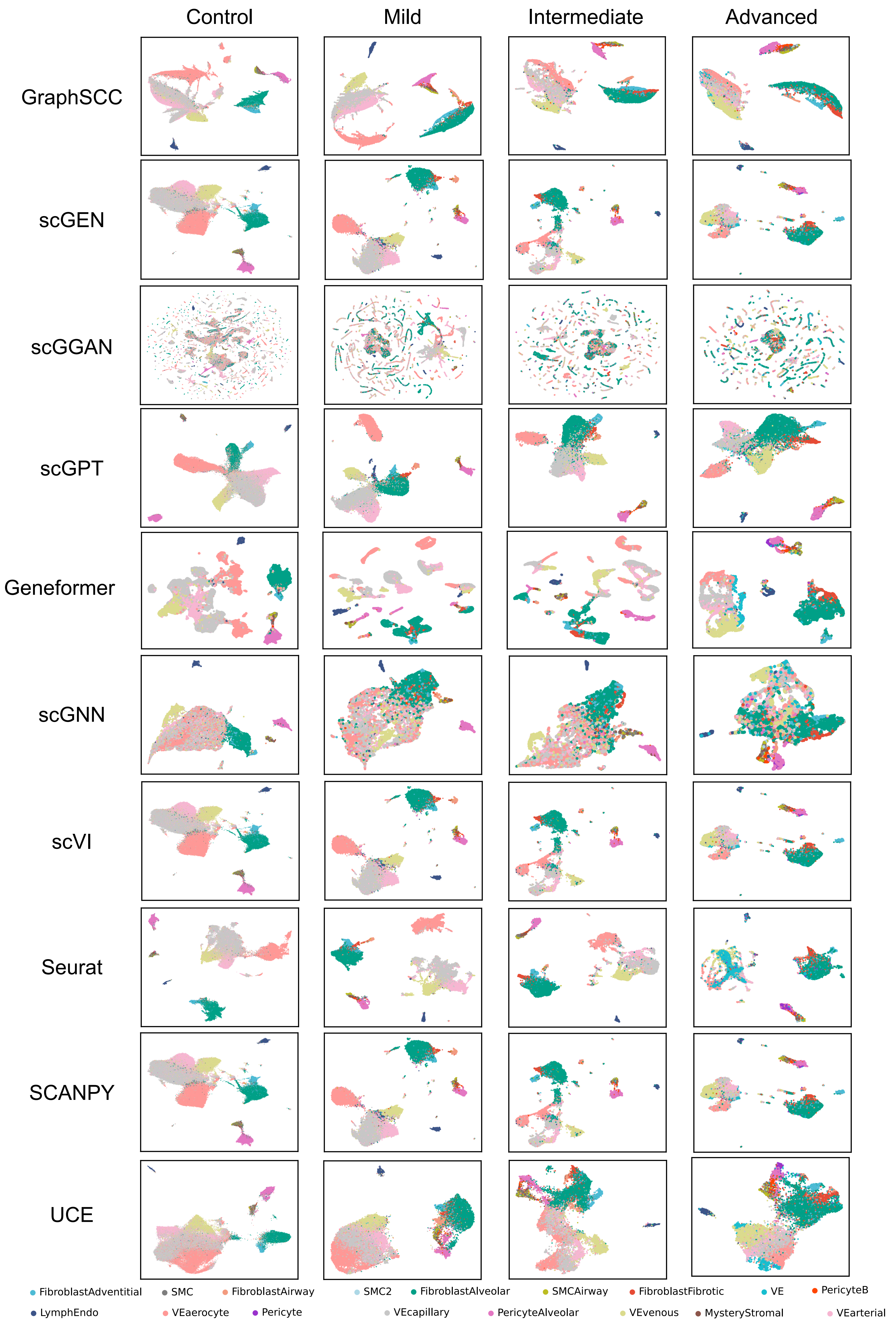

Supplementary Fig. 13 UMAP Visualizations of independent manual cell-type annotations for benchmarking methods in IPF data. This figure presents UMAP visualizations for each benchmarking method across four distinct tissue fibrosis grades in IPF: Control, Mild fibrosis, Intermediate fibrosis, and Advanced fibrosis, displayed from left to right. In these visualizations, each point represents a cell, with coloring based on cell type as determined by the independent and manual annotations.

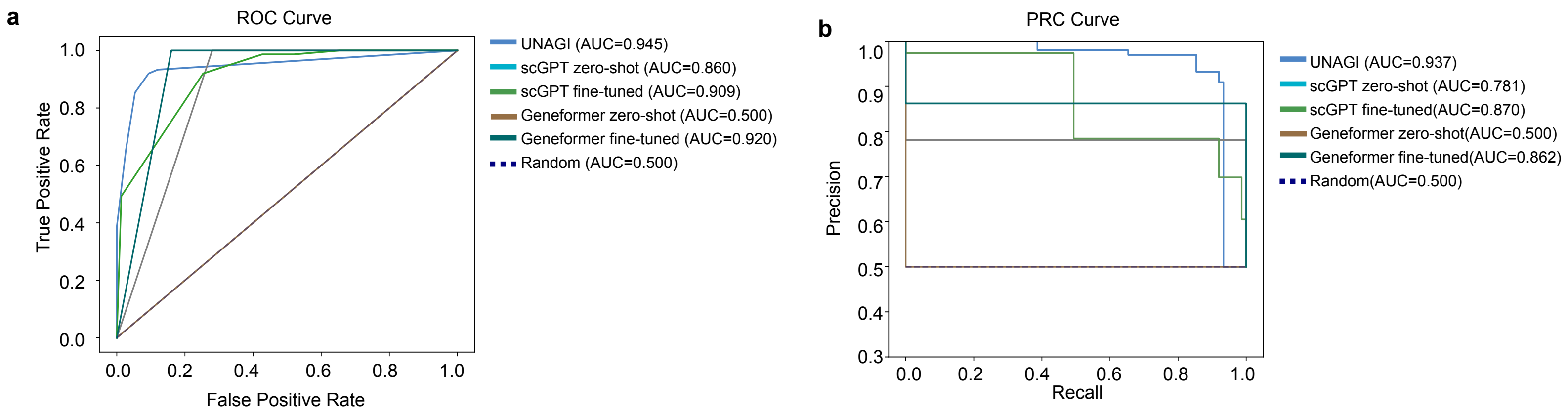

Supplementary Fig. 14, Benchmarking the in-silico drug screening capability of UNAGI against foundation models before and after fine-tune using the IPF dataset. a, ROC curves for in-silico drug screening performance, showing true positive rate vs. false positive rate. **b**, PRC curves for precision and recall in in-silico drug screening.

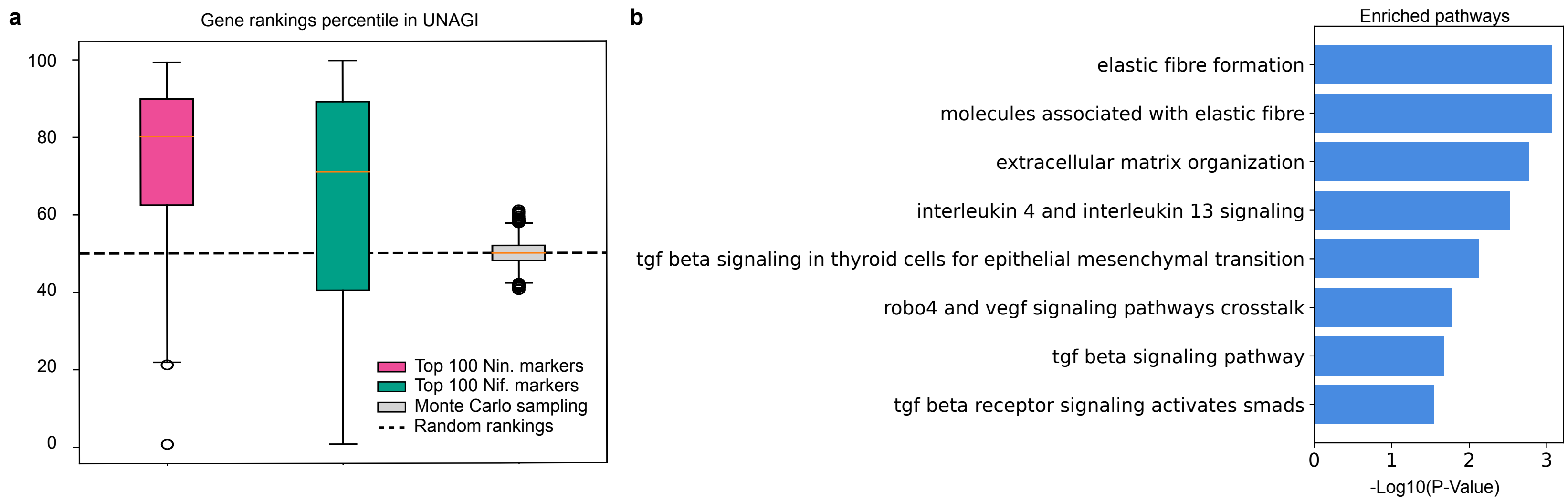

Supplementary Fig. 15 The analysis of top weighted genes learned by UNAGI in the scRNA-seq data from PCLS experiments. a, The gene-weight rank percentile of treatment markers and ECM markers (n=100). The median percentiles are: the median of top 100 Nintedanib treatment markers: 81st , and the median of the top 100 Nifedipine treatment markers: 71th. The boxes represent the interquartile ranges (IQRs), and the solid lines indicate the medians. The whiskers extend to points within 1.5 IQRs of the lower and upper quartiles. **b**, The pathways enriched in the top 100 weighted genes learned by UNAGI are closely related to the development of fibrosis. We applied one-sided hypergeometry test and FDR correction using the BH procedure in panel b.

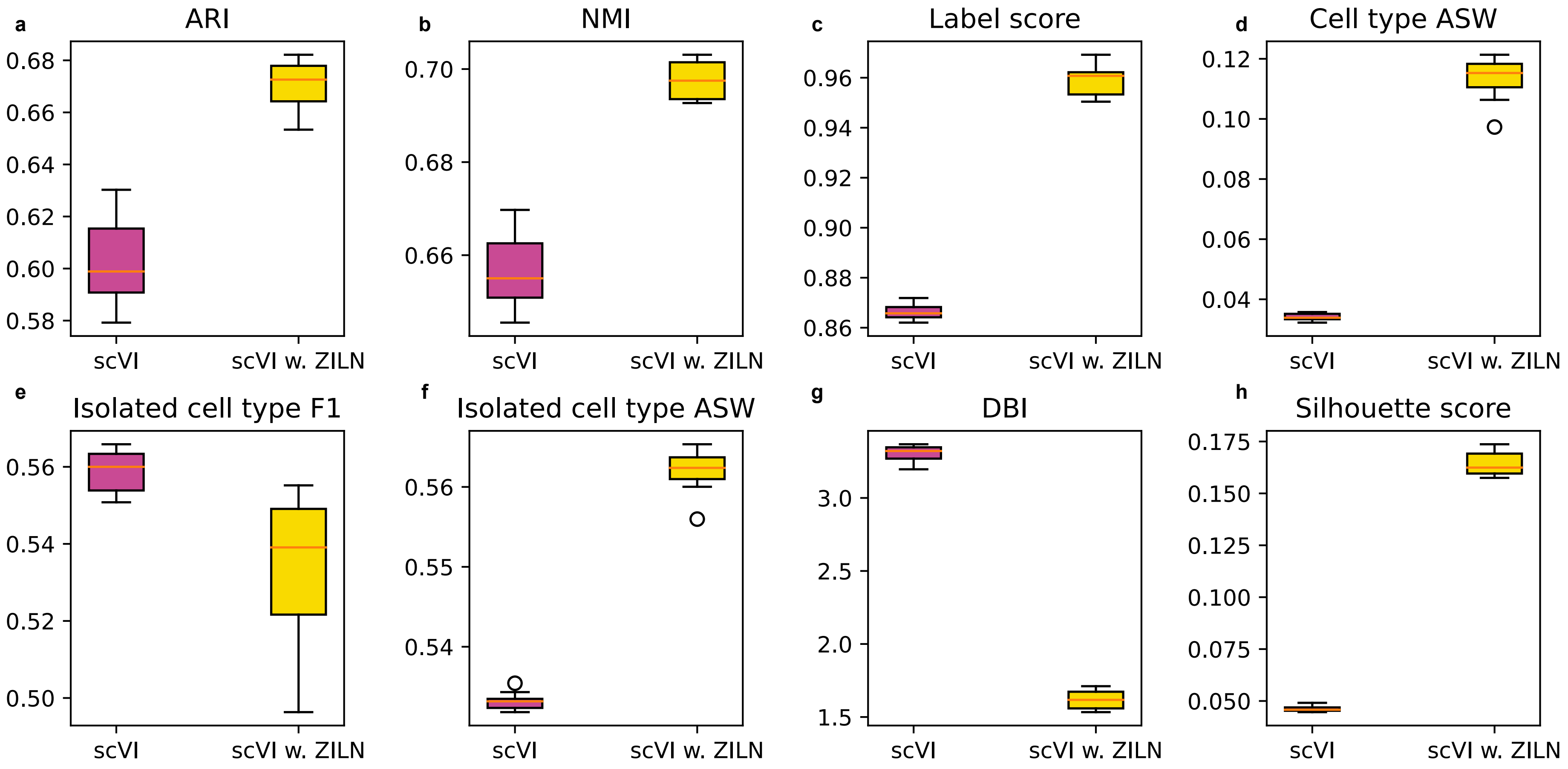

Supplementary Fig. 16 ZILN distribution can help other models improve their embedding quality in the IPF dataset. a, Adjusted Rand Index (ARI). **b**, Normalized Mutual Information (NMI). **c**, Label score. **d**, Cell type ASW. **e**, Isolated cell type F1 score. **f**, Isolated cell type ASW. **g**, Davis-Bouldin index (DBI); a lower DBI signifies better clustering. **h**, Silhouette score. After applying ZILN distribution to the scVI model, it helps scVI to better model the IPF dataset. The experiments in panels a-h run with different seeds (n=10).The boxes represent the interquartile ranges (IQRs), and the solid lines indicate the medians. The whiskers extend to points within 1.5 IQRs of the lower and upper quartiles.

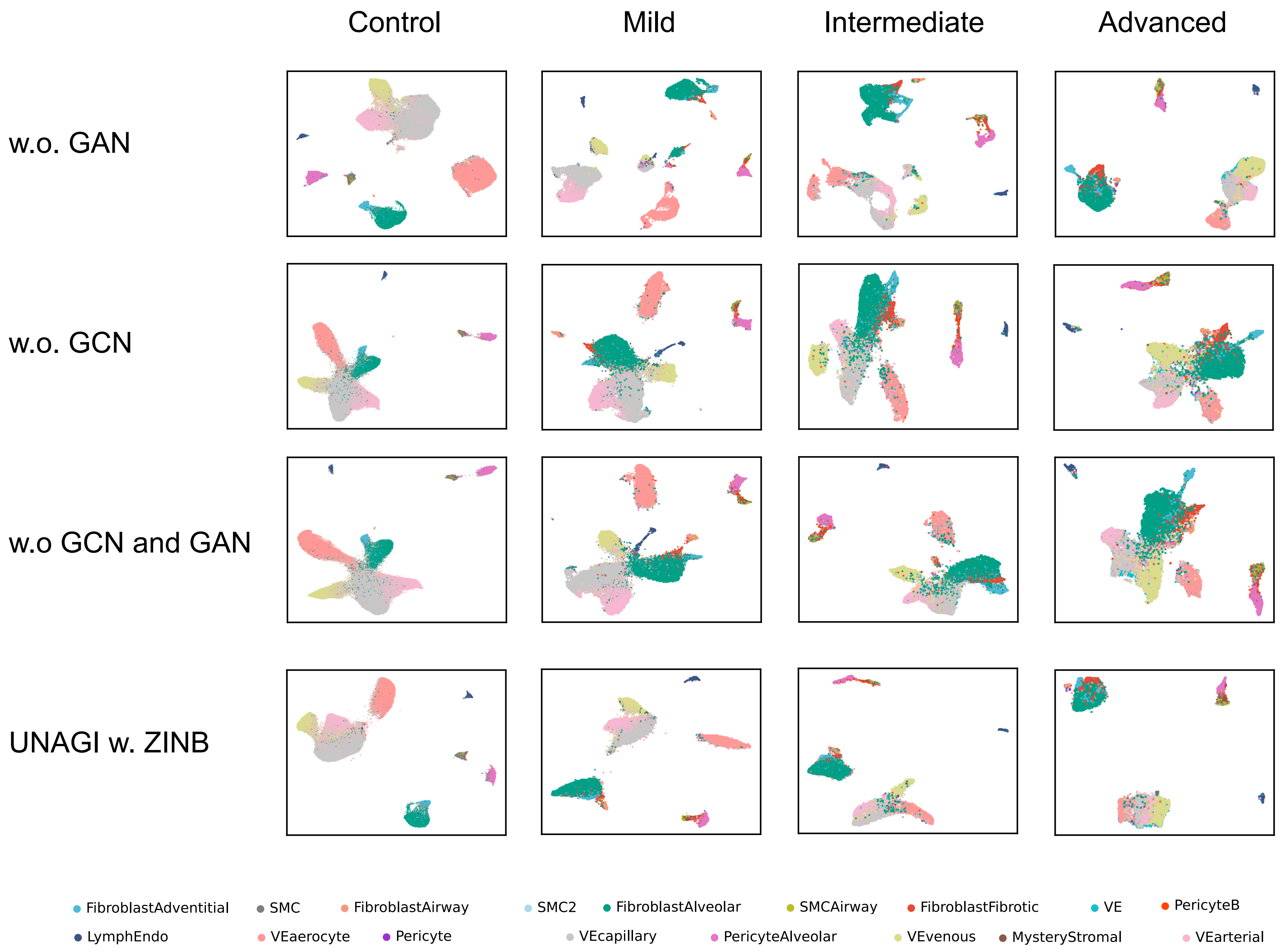

Supplementary Fig. 17 UMAP Visualizations of independent manual cell-type annotations for ablations. This figure presents UMAP visualizations for ablation studies across four distinct tissue fibrosis grades in IPF: Control, Mild fibrosis, Intermediate fibrosis, and Advanced fibrosis, displayed from left to right. In these visualizations, each point represents a cell, with coloring based on cell type as determined by the independent and manual annotations.

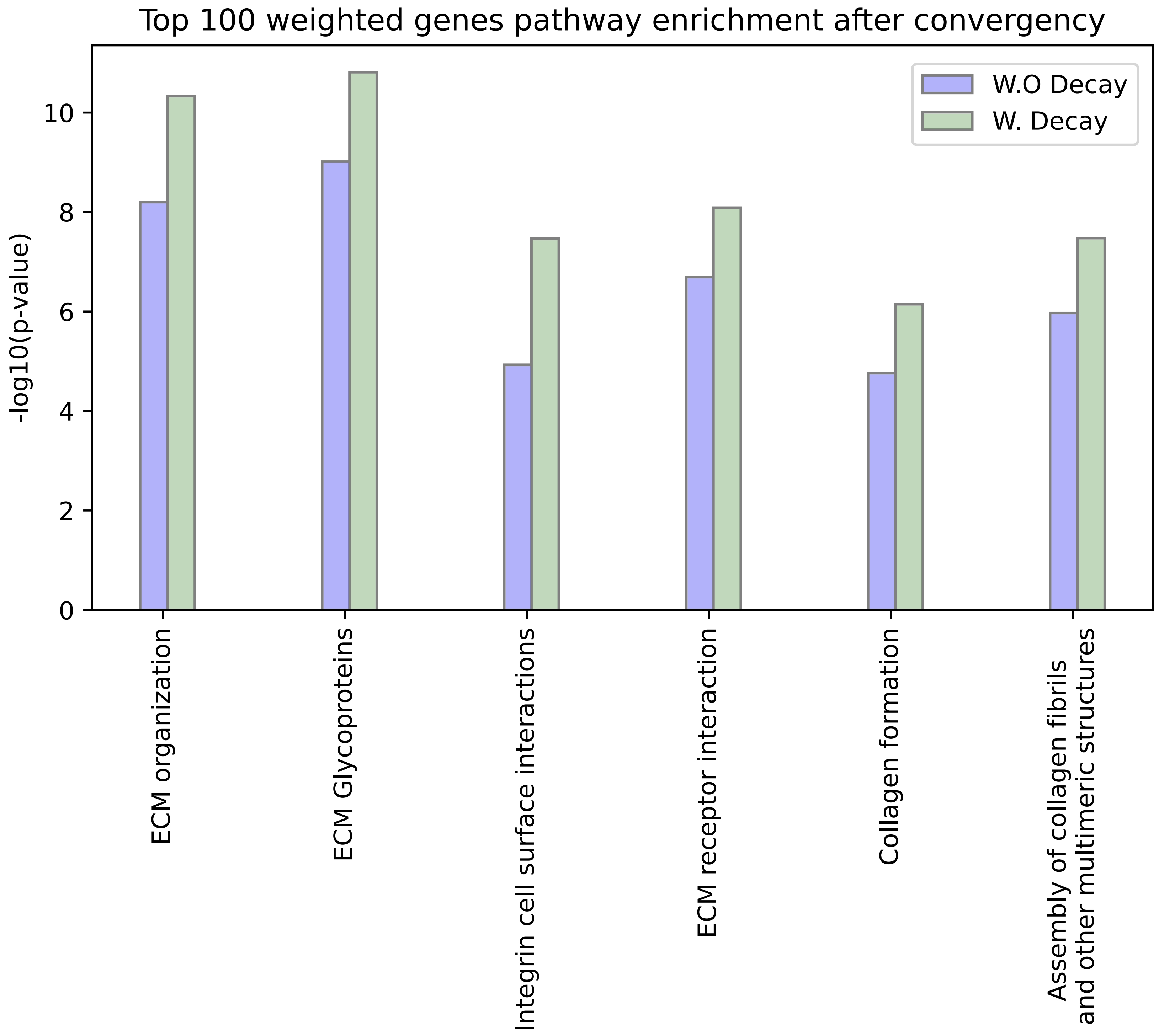

Supplementary Fig. 18 The bar plots display the P-values of the overlapped pathways enriched in the top 100 weighted genes from the converged UNAGI model using or not using gene-weight decaying strategy. We applied one-sided hypergeometry test and FDR correction using the BH procedure

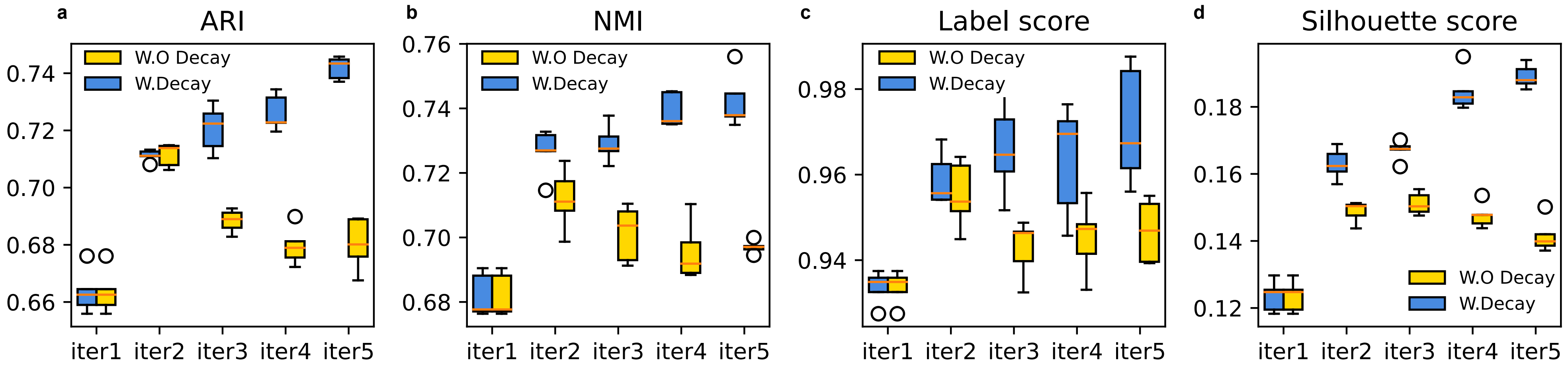

Supplementary Fig. 19 The metrics of embedding quality of UNAGI with and without gene weight-decay strategies across iterations. In the iteration 1, genes were weighted equally. Both strategies were used for subsequent training on the same model, which was pre-trained on iteration 1. The experiments in panels a-d run with different seeds (n=5). The boxes represent the interquartile ranges (IQRs), and the solid lines indicate the medians. The whiskers extend to points within 1.5 IQRs of the lower and upper quartiles.

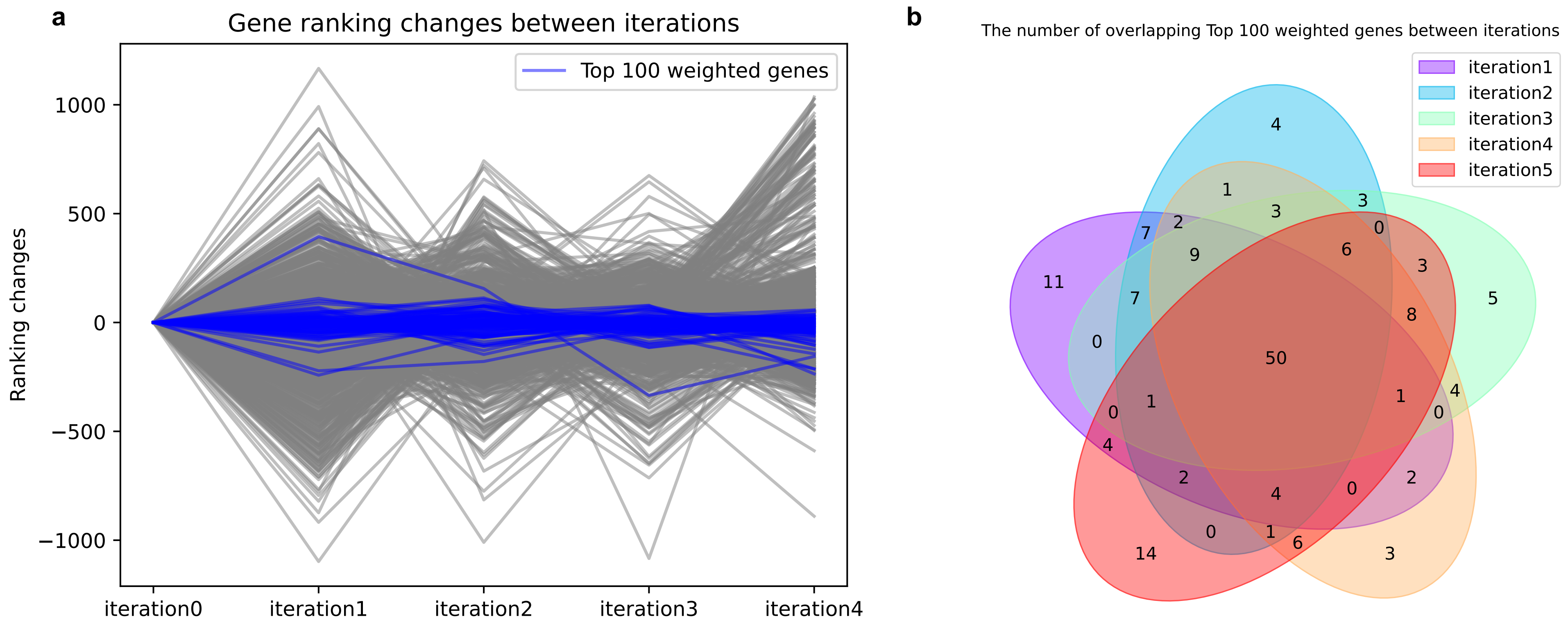

Supplementary Fig. 20 Gene ranking changes between iterations of training on the IPF dataset. a, The line plot displays gene ranking changes between iterations. The top 100 weighted genes in iteration 5 are highlighted in blue. **b,** The Venn5 plot shows the number of overlapped top 100 between iteration 1 and iteration 5. Half of the top 100 weighted genes is consistent in all 5 iterations.

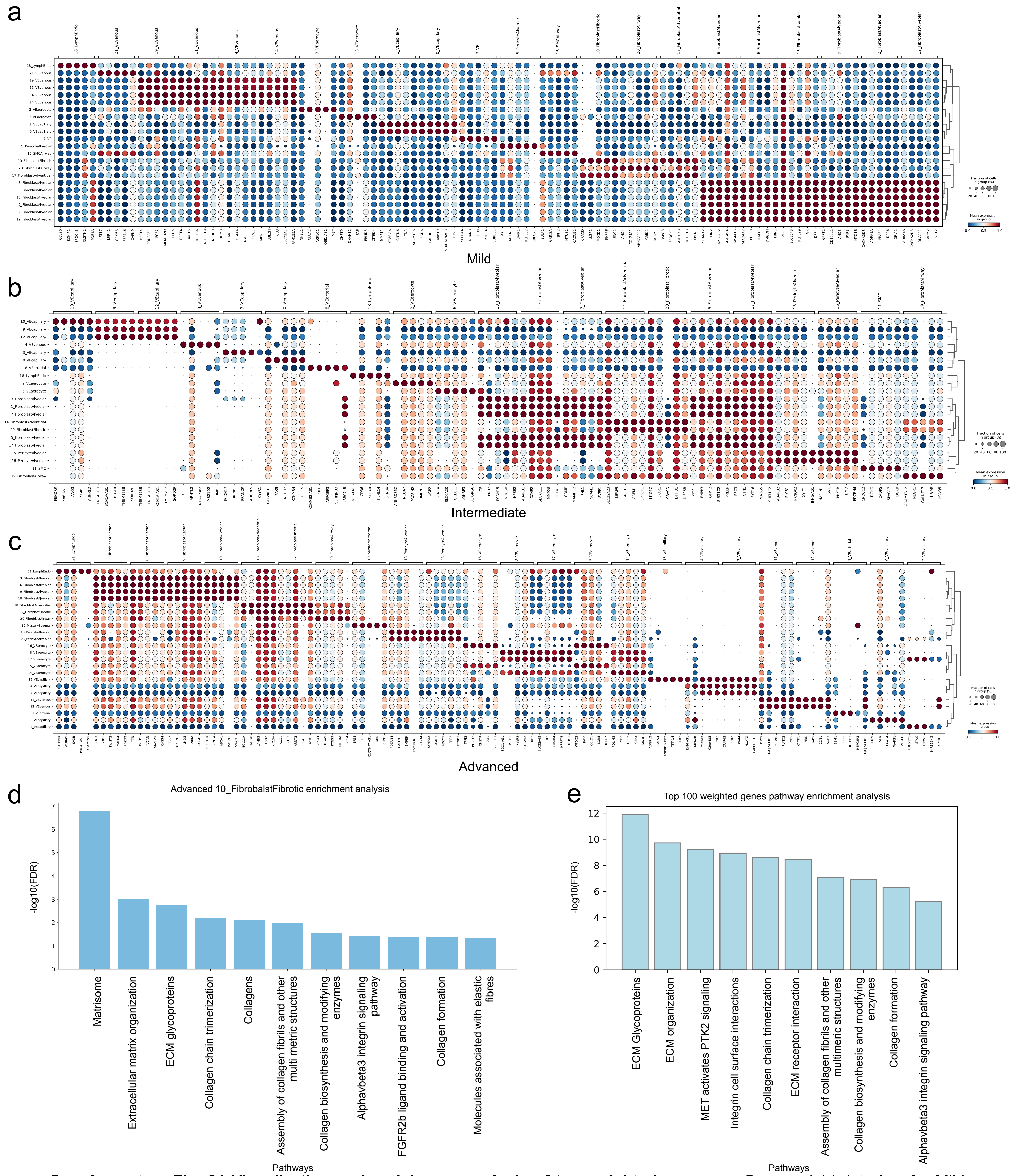

Supplementary Fig. 21 Visualization and enrichment analysis of top weighted genes a, Gene weight dot plots for Mild fibrosis, displaying the top weighted genes for each cluster. Weights are normalized on a scale from 0 (no weight) to 1 (peak weight). **b**, Gene weight dot plots for Intermediate fibrosis with the same normalization **p**arameters as panel a. **c,** Gene weight dot plots for IPF Advanced, again showing weights normalized between 0 and 1. **d**, Bar plot illustrating the pathway enrichment score for the top 100 weighted genes in the 10_FibroblastFibrotic cluster of Advanced fibrosis. The y-axis represents the $-\log 10(\mathrm{FDR})$, while the x-axis lists the pathways. Pathways enriched among the top 100 weighted genes in the 10_FibroblastFibrotic cluster show significant associations with IPF. **e**, Bar plot of pathway enrichment score of top 100 weighted genes in the IPF dataset, where the x-axis is the pathways, and y-axis is the –log10(FDR). The pathway enrichment analysis reveals a strong association between top 100 weighted genes and the IPF progression. We applied one-sided hypergeometry test and FDR correction using the BH procedure in panels d and e.

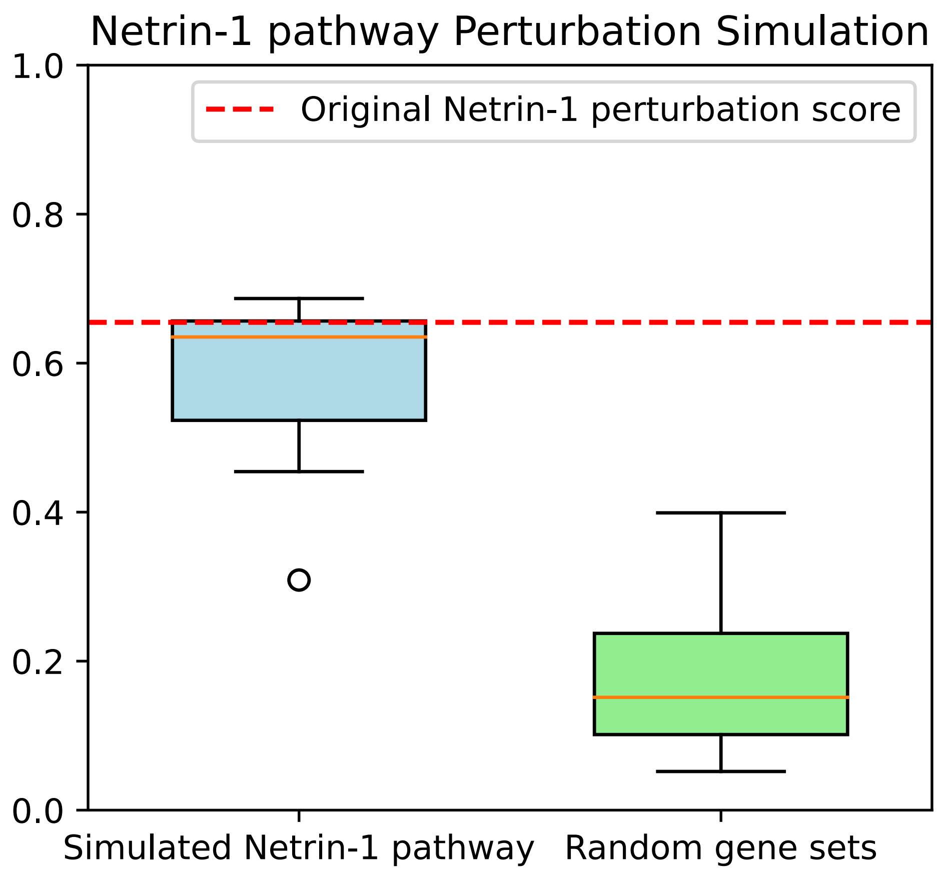

Supplementary Fig. 22. The boxplot shows the perturbation results using the simulated Netrin-1 pathway perturbation and random gene sets in FibAlv-4 track. The red line represents the original Netrin-1 pathway perturbation scores from FibAlv-4 track. The experiments run with different seeds (n=100). The boxes represent the interquartile ranges (IQRs), and the solid lines indicate the medians. The whiskers extend to points within 1.5 IQRs of the lower and upper quartiles.

Supplementary Note 1

Model Architecture

Model architecture of Graph VAE

Layer	Shape
GCN1(graph convolution layer)	[input_genes, 1024]
Encoder_FC1 (linear layer)	[1024, 256]
Encoder_FC2_mean (linear layer)	[256, 64]
Encoder_FC2_log_var (linear layer)	[256, 64]
Decoder_FC3 (linear layer)	[64, 256]
Decoder_FC4_expectation (linear layer)	[256, input_genes]
Decoder_FC4 _dropout_logits (linear layer)	[256, input_genes]

Model architecture of Discriminator

Layer	Shape
FC1 (linear layer)	[input_genes, 128]
FC2 (Linear layer)	[128, 64]
FC_classifier (linear layer)	[64, 1]

Default training parameters

Parameters	value
Graph VAE learning rate	1e-4
Discriminator learning rate	5e-4
Distribution	'ZILN'
Batch Size	2048
Epoch for initial iteration	3
Epoch for subsequent iterations	5
Number of neighbors in the KNN graph	50
τ (Parameter controls the influence of gene weights of the reconstruction loss in the iterative training)	0.5
Decrement rate of iterative training	0.3
steepness weight to build the Gene interaction network for in-silico perturbation	0.2
Scaling factor for the perturbation score	100

Supplementary Note 2

Gaussian mixture density estimator

In this study, we utilized micro-CT technology to quantify tissue fibrosis grades based on alveolar surface density. This measurement does not necessarily correspond to traditional clinical stages of IPF, as is often the case with other diseases like COVID-19. To avoid potential confusion, we opted to describe these levels as 'tissue fibrosis grades rather than 'disease grades' in the context of IPF. The Gaussian Mixture Model (GMM) clustering method[1] leverages multivariate Gaussian components to characterize various tissue fibrosis grades of IPF samples. This approach aims to

categorize samples into different grades of the disease, optimizing the probability or density representation of these grades. In this study, the GMM clustering approach is founded on the concept of surface density, serving as a measure of the extent of tissue fibrosis. We assume that the surface density of all samples is independently and identically distributed, represented as $S_{density} = \{s_1, s_2, \dots . s_{\aleph}\}$, where $\aleph$ is the total number of samples. The GMM is fitted to the data to identify optimal components that maximize the log-likelihood: $P\left(S_{density} \middle| \mu_{1,\dots,c}, \sigma_{1,\dots,c}\right) = \sum_i^c \log \mathcal{N}\left(S_{density} \middle| \mu_i, \sigma_i\right)$. Here, $\mathcal{N}$ represents the Gaussian density function of the GMM for each sample, and c is the total number of Gaussian components, each characterized by a mean μ_i and standard deviation σ_i . After identifying the optimal components, the samples S and their corresponding cells are softly classified into different tissue fibrosis grades, constructing the dataset $X = X_1, \dots, X_T$ where each $X \in R^{m_T \times n}$. Here, T refers to the number of tissue fibrosis grades, and each tissue fibrosis grade has m_T cells with n genes.

Supplementary Note 3

Sanity perturbation approach

To evaluate the effectiveness of our proposed in-silico perturbation strategy, we also employed a sanity perturbation approach. This involved randomly selecting a specific track from the temporal dynamics graph and calculating the average gene expression at each disease grade along this track to determine the centroid for each grade. For all disease grades other than the control, we adjusted the gene expression of all cells within each disease grade to match that of the preceding disease grade. This was achieved by subtracting the centroid differences between these disease grades on each of the cells from the grade. Subsequently, the perturbed cells were input into the UNAGI model, following the 'In-silico perturbation strategies' and 'In-silico perturbation scoring' sections above to obtain the perturbed cell embeddings. We then calculated the perturbation scores, which served as a metric to evaluate the effectiveness of our perturbation strategy. Sanity perturbations should result in positive and far larger therapeutic scores compared to random perturbations.

Supplementary Note 4

Details of dataset description and preprocessing

Reasons for using snRNA-seq than scRNA-seq

The advantage is that snRNA-seq technology can be conducted on frozen samples while scRNA-seq can only process fresh samples[2]. snRNA-seq's drawback is the loss of cytoplasmic transcripts and lower data quality compared to scRNA-seq. It is challenging to collect fresh tissue samples from rare diseases like IPF, though scRNA-seq can better profile cells. In addition, the quantification of surface density can only be achieved through the microCT characterization of frozen tissues, which imposes limitations on the utilization of scRNA sequencing.

Step-by-step IPF data preprocessing

After sequencing, raw fastq files were trimmed with cutadapt[3] (version 1.17) to remove read2 contamination of 5-prime template switch oligo and 3-prime polyadenylated tails; read pairs were discarded if read2 was trimmed below 30 bases. Trimmed reads were mapped to GRCh38 annotated with GENCODE[4] (release 37) with the STARsolo[5] implementation of STAR (v2.7.6a); the barcode whitelist file and barcode length parameters were based on the manufacturer's (10X Genomics) guidelines for 3-prime v3.1 scRNAseq assays. Transcript count information was taken from STAR's unfiltered 'GeneFull' output, all barcodes with at least 300 transcripts were imported into R (v 4.0.5) alongside statistics for each barcode's percent of transcripts spliced, unspliced, or ambiguous from STAR's 'velocyto' output. Initially, we have 60,651 genes of each cell in the IPF data. Cell barcode cleaning and cell type annotation were performed with tools from the R package Seurat (v4.1.1). To conduct an independent and manual cell type annotation, each sample of data was subjected to an iterative and recursive process of dimension reduction, graph embedding, and cluster analysis. After each iteration, the cell type labelling is refined, spurious nuclei are removed, and a subset of relatively similar cell types is isolated for the next iteration. This process was repeated until all spurious nuclei were removed and new cell subpopulations could no longer be resolved. After each sample was cleaned and annotated, all samples were combined. Seurat's reciprocal PCA integration method was used to adjust for batch effects at the cDNA library level. The gene expression results generated from integration were used for a final iteration of UMAP embedding and clustering. Final cell type assignments (the 'Ground Truth' column in Supplementary Fig 1) were determined by evaluating the true (not integrated) gene expression marker signatures of a cluster and confirming that the pattern was consistent across each sample. Please note that all subsequent analyses using the deep generative model solely utilize the normalized cell-by-gene matrix obtained from this preprocessing step. These analyses are augmented with manual cell type annotations performed independently via the Seurat pipeline, as detailed in this subsection. Crucially, all cell embeddings and clustering results presented in this manuscript were produced using our deep generative neural network framework, not the Seurat pipeline. The role of the Seurat framework was strictly confined to data preprocessing.

Supplementary Note 5

Clustering parameters optimization

To maintain consistency in cluster numbers and sizes, as well as the distances between cell neighbors, across various disease grades, we introduce a Clustering Parameter Optimization (CPO) method. Connectivity graph-based community detection clustering methods, like Leiden clustering[6], can automatically identify the number of clusters. However, an improper number of neighbors in the neighborhood graph or an improper resolution setting can lead to over-clustering or under-clustering, introducing complications in the analysis process. The consistency in the number and size of clusters is important for tracing the lineage of cell populations through various grades of development or disease progression. The proposed CPO method encompasses two primary steps. (1) Searching for the optimal number of neighbors to construct graphs with

consistent cell-neighbor distances across different disease grades. We start by selecting an anchor disease grade, which is the disease grade with a cell count closest to the median count of all disease grades, denoted as N_{anchor}. We then calculate the average distance between cells and their neighbors in this anchor disease grade, establishing the anchor neighbor distance. The goal is to find a number of neighbors that yields a neighbor-distance similar to that of the anchor disease grade. (2) The second step involves determining the optimal clustering resolution. We aim to find a set of resolutions within the predefined range [R_{min} = 0.8, R_{max}=1.5] for different disease grades that result in a similar median number of cells per cluster across these grades. For different application scenarios, users have the option to select a resolution range larger than the default setting. This flexibility enables adaptation to various analytical needs and preferences. By employing the CPO method, we ensure that the neighborhood graphs for different disease grades maintain similar cell-neighbor distances. Additionally, this approach ensures a consistent number and size of clusters across different disease grades, thereby enhancing the coherence and robustness of our analytical framework.

Supplementary Note 6

Using iDREM (Interactive Dynamic Regulatory Events Miner) to reconstruct the temporal gene regulatory network underlying disease progression

We employ iDREM (Interactive Dynamic Regulatory Events Miner)[7], a machine learning model based on an Input-Output Hidden Markov Model, to reconstruct the temporal gene regulatory network underlying each track (i.e., associated with each cell population) in the reconstructed cellular dynamics graph $G_{dynamic}$. This gene regulatory network consists of co-expressed genes and gene regulators that modulate the temporal progression of the disease within each cell population. For each track in $G_{dynamic}$, iDREM identifies the genes that undergo similar expression change patterns throughout the disease progression, which were termed as gene paths, some with increasing expression patterns while others with decreasing patterns. For each of the identified co-expressed gene paths, iDREM also provides its enriched GO terms and pathways. Beyond the identification of co-expressed gene paths, iDREM also captures the gene regulators that modulate those gene paths during disease progression. The dynamic genes and gene regulators identified through this process are considered dynamic marker candidates and hold potential as therapeutic targets for the disease.

Supplementary Note 7

Weight-decay based iterative training strategy

Cell mis-clustering can lead to incorrect weight accumulation, causing noisy genes to receive high weights by chance and disrupting the iterative training process. With weight decay, genes strongly linked to disease progression maintain consistent weight increases, while the weights of noisy

genes are reduced in every iteration, preventing them from accumulating by the end of the training. Supplementary Fig. 18 demonstrates that using the weight-decay strategy results in more significant FDR for disease-associated pathways enriched in the top 100 weighted genes. This strategy also improves the embedding quality across iterations, as shown in Supplementary Fig. 19. The decrement rate, a hyperparameter, was empirically set to 30%. While decreasing weights can address mis-clustering issues, zeroing them out or using a higher decrement rate may erase the model's understanding of disease. A moderate decrease rate maintains the model's adaptability, dynamically adjusting and enhancing its understanding of gene significance throughout iterations. Despite fluctuations in gene rankings between iterations, the top 100 weighted genes exhibit consistent stability across various iterations when employing the weight-decay approach (Supplementary Fig. 20).

Supplementary Note 8

Dynamic and hierarchical static markers discovery

To characterize the temporal progression of the disease for each cell population, we identify dynamic markers which are genes that change considerably throughout the disease's progression. For each track in the cellular dynamics graph, iDREM identifies the gene paths with co-expression patterns during disease progression. Next, we compute the sum of fold changes for each gene in each of the gene paths across all disease grades associated with each cell dynamic track (i.e., a cell type or subtype). Genes in those identified gene paths were considered as candidate dynamic biomarkers. To calculate the statistical significance of the candidate markers, we randomly shuffle cells within each disease grade of the track to generate the background simulation tracks. We then calculate the accumulated fold changes among all neighboring grades of these candidate markers across all simulation tracks. This simulation process was repeated N times (N >1000) to establish a random background fold change distribution. We then evaluated the P-values for each candidate marker based on its accumulated sum fold change against this background distribution. To ensure a high level of confidence in our selection of dynamic markers, we impose a more stringent FDR cut-off (FDR < 0.01) than the default (FDR <0.05). These selected dynamic markers are important in delineating the progression tracks and provide a detailed understanding of the longitudinal evolution of the disease within each distinct cell population.

The hierarchical static marker discovery approach supports the identification of intra-disease grade static markers through hierarchical clustering. UNAGI conducts hierarchical clustering based on the embeddings of cell populations at each disease grade, thereby generating dendrograms to depict the relationships among these populations. Initially, we identify distinct cell populations using their latent embeddings Z. Hierarchical clustering is then applied to these embeddings to construct a dendrogram for the cell populations within the same disease grade. This dendrogram serves as a tool to explore the hierarchical structure of cell populations. In this dendrogram, when focusing on a particular cluster, we analyze it at various levels to identify hierarchical static

markers. At lower levels of the dendrogram, the selected cluster compares with a broader range of sibling clusters. Here, the hierarchical static markers identified tend to represent general features of the cell population, as the siblings encompass a wider scope. For instance, at level 0, the selected cluster is compared against all other clusters within that disease grade. Conversely, at higher dendrogram levels, the siblings are more closely related to the selected cluster. This closeness allows for the identification of markers that highlight the subtle heterogeneities among cell subpopulations within the same cell type. By examining these differences, we can gain a deeper understanding of the cell subpopulation's characteristics.

Supplementary Note 9

Proteomics data validation protocols, experiments and preprocessing

Briefly, the tissue samples were then homogenized using a Qiagen TissueLyser II with a 2 × 24 adapter (chilled to −20°C) following vendor's instruction[8]. The aqueous polar metabolites and proteins were extracted from each homogenate using the MPLEx protocol. Keeping each sample on ice, a volume of chilled chloroform and water were added to a final ratio of 3:8:4 water-chloroform-methanol, mixing gently after each addition. The samples were chilled on ice for 5 min before mixing well and separating the layers by centrifugation (10,000 g, 10 min, 4°C). Proteins were isolated and concentrated to dryness in a vacuum concentrator and stored at −70°C until ready for further processing. For each sample proteins were denatured, alkylated, digested with trypsin, and desalted on a C18 solid-phase extraction (SPE) cartridge using previously detailed methods[9]. For the analyses, 5 µL of resulting peptides at the concentration of 0.1 µg/µL were analyzed by reverse phase liquid chromatography coupled with an Orbitrap Lumos instrument (Thermo Scientific) in data-dependent mode (DDA). Briefly, samples were loaded on an SPE column via a 5 µL sample loop separated by the C18 column using a 120 min gradient. The effluents from the LC column were ionized by electrospray ionization and introduced into the mass spectrometer via a heated capillary maintained at 250°C for ion desolvation. The resulting ions were mass analyzed by the Orbitrap at a resolution of 60,000 covering the mass range from 300 to 1,800 Da. The top 12 most intense ions were then targeted for fragmentation per cycle time. Tandem mass MS2, ions were isolated by quadrupole mass filter in monoisotopic peak selection mode using an isolation window of 0.7 Da, maximum injection time of 50 ms with AGC setting at 1E5 ions and fragmented by high-energy collision dissociation (HCD) with nitrogen at 32% normalized collision energy. Fragment ions were mass analyzed by the Orbitrap at a resolution of 7,500, and spectra were recorded in the centroid mode. Ions once selected for MS2 were dynamically excluded for the next 45s. The instrument raw files are publicly available on MassIVE (Server: massive.ucsd.edu, User: MSV000093129, Password: Lung5172). The raw files were analyzed using MaxQuant v1.6.0.16 LFQ quantification. Downstream data processing and statistics were performed using RomicsProcessor[10]. The resulting LFQ intensities were log2 transformed and median-centered. ANOVA and Student's T-test were performed, and FDR

corrections were applied. The data processing code is available on GitHub (https://github.com/GeremyClair/IPF_DDA_proteomics/).

Supplementary Note 10

Precision-cut lung slice (PCLS) data preprocessing

Basecalls were converted to reads with the software Cell Ranger's[11] (v4.0.0) implementation mkfastq. Multiple fastq files from the same library and strand were catenated to single files. Read2 files were subject to two passes of contaminant trimming with Cutadapt[3] (4.1) for the template switch oligo sequence anchored on the 5′ end and for poly(A) sequences on the 3′ end. Following trimming, read pairs were removed if the read2 was trimmed below 30 bp.

Paired reads were filtered if either the cell barcode or unique molecular identifier (UMI) sequence had more than 1 bp with a phred of <20. Reads were aligned with STAR[5] (v2.7.9a) to the human genome reference GRCh38[4] release 99. After preprocessing, analysis of the ex vivo human PCLS snRNA-seq data was conducted using the Seurat[12] package (version 1.8.2). Cells with less than 750 transcripts profiled were then removed.

To minimize the possible effect of potential batch correction methods, we first processed and annotated each library separately, before integrating them together and annotating them jointly. To integrate the multiple snRNA-seq datasets, we employed Robust Principal Component Analysis (RPCA)[13]. Based on the cellular diversity, we chose to use PCLS treated with DMSO as the reference for the integration. Following the RPCA decomposition, we utilized the low-rank component as the integrated representation of the snRNA-seq datasets. This component captured shared biological signals across conditions while mitigating dataset-specific variations. Subsequent analyses, such as clustering and differential expression analysis, were performed on the non-integrated but normalized gene expression values. To validate the effectiveness of the integrated representation, we performed various analyses, including cell-type clustering, and identification of marker genes. We also compared the results of these analyses to those obtained from individual datasets to evaluate the improvement gained through the integration process. Marker genes were computed using a Wilcoxon rank-sum test, and genes were considered marker genes if the FDR was below 0.05 and the log2 fold change was above 0.5.

Supplementary Note 11

Benchmarking against Geneformer

Geneformer[14] is a Bert-based single-cell foundation model to generate low-dimensional single-cell embeddings. We adopted the latest version (April 2024) of Geneformer model which was trained on 95 million cells. Originally, Geneformer was designed for zero-shot single-cell analysis tasks. The Geneformer is built based on the Bert architecture provided by Hugging-face which

allowed us to fine tune the model using Hugging-face's APIs. Supplementary Note Fig. 1 shows that fine-tuning the Geneformer can largely improve the performance of the Geneformer model. Thus, we benchmarked UNAGI against Geneformer under the fine-tuning setting. By sending the perturbed gene expression to Geneformer, it can generate embeddings of the perturbed cells. Thus, Geneformer can implement the same unsupervised in-silico drug discovery function as UNAGI. Geneformer does not natively support predicting the gene expression after the perturbation due to its Bert-based structure, which only has the encoder to generate low-dimensional cell embeddings.

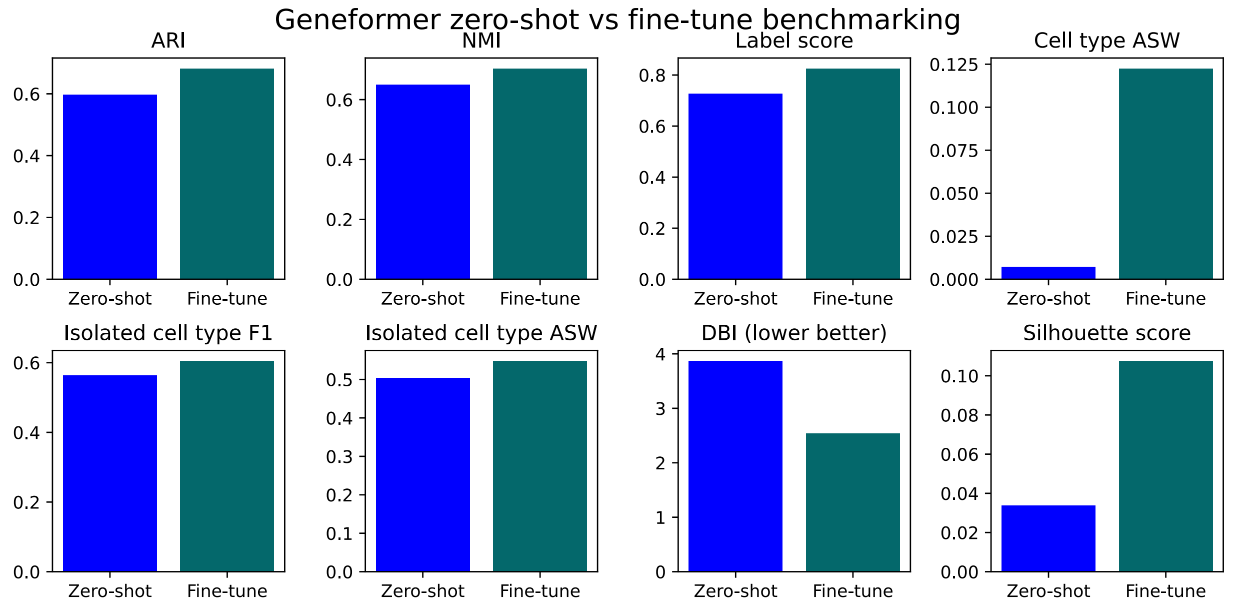

Supplementary Note Fig. 1 Embedding quality of IPF dataset generated by the zero-shot and fine-tune modes of the Geneformer model. The fine-tune scGPT model outperforms the zero-shot setting.

Benchmarking against Universal Cell Embeddings

Universal Cell Embeddings[15] (UCE) is a single-cell foundation model which was trained on the 36 million cells. UCE is specifically designed for zero-shot tasks and does not support fine-tuning. Thus, the benchmarking of UCE can only be conducted in the zero-shot setting. We showed the quality of cell embeddings generated by UCE and other single-cell foundation models in both zero-shot and fine-tuning settings in the Supplementary Fig. 12.

We excluded UCE from the in-silico drug screening benchmarking due to its high computational complexity (Extended Data Fig. 4c).

Supplementary Note 12

Details of embedding quality benchmarking

In our IPF dataset, we provided hand-craft cell type annotations derived by recursively annotating and refining cell types on individual samples (Please refer to "Dataset description and preprocessing" for details). Thus, the results from the standard Seurat pipeline cannot serve as a perfect proxy for the ground truth scores to evaluate the clustering results from other methods. For individual methods, we set the size of embedding dimension as 64, except for scGPT, Geneformer, and UCE which are restricted to generating embeddings with 512, 896, and 1280 dimensions, respectively. We conducted fine-tuning of the scGPT and Geneformer model using benchmarking datasets, and subsequently utilized the acquired cell embeddings to carry out clustering and other downstream tasks. GraphSCC, scGGAN, and scGNN require predefined information on the number of cell types, influencing their clustering approaches. GraphSCC and scGGAN use the number of cell types directly in k-means clustering, and scGNN uses it in the k-means followed by Louvain clustering. We adjusted parameters for individual methods to have similar number of clusters for fairness in comparison.

Supplementary Note 13

Build the simulation data for in-silico drug screen simulation

We ranked drugs based on their targets' average expression and using the lowest 25 drugs for the simulation study. The rationale behind this approach is to avoid the influence of genes that have substantial signals in disease progression, thus ensuring that the simulation results are due to the simulation itself rather than pre-existing conditions. It allows us to demonstrate the effectiveness of the simulation by focusing on target genes rather than the types of drugs chosen. We only simulate one drug in each simulation dataset. To build a positive drug simulation dataset, we first shuffled gene expression of the snRNA-seq IPF data at individual tissue fibrosis grades. Then, for down-regulated drugs, we manually added $[B, 2B, .., T * B]$ ($B > 0$, T is the total number of tissue fibrosis grades) to the drug target gene expression in fibroblast cells. Similarly, for up-regulated drugs, we manually added $[T * B, (T-1) * B, ..., B]$ to the drug target gene expression in fibroblast cells. For the negative drug simulation dataset, we randomly sampled from a normal distribution at individual tissue fibrosis grades and added it to the shuffled dataset. If the new expression value is negative, it is set as 0. For simulated datasets, we randomly masked target gene expression according to the dropout rate in the original dataset. We set B as [0.2, 0.3, 0.4] in our experiment and each drug had three corresponding positive and negative simulation datasets.

Supplementary Note 14

Benchmarking against scGPT

scGPT[16] is a foundational single-cell model for generating low-dimensional single-cell embeddings and supporting supervised in-silico perturbation. scGPT can be deployed in either a zero-shot setting (directly applying the pre-trained foundation model to new data) or a fine-tuning setting (fine-tuning the pre-trained model using new data). We examined the performance of

scGPT in generating cell embeddings for the IPF dataset and decided to use the fine-tune strategy (Supplementary Note Fig. 2).

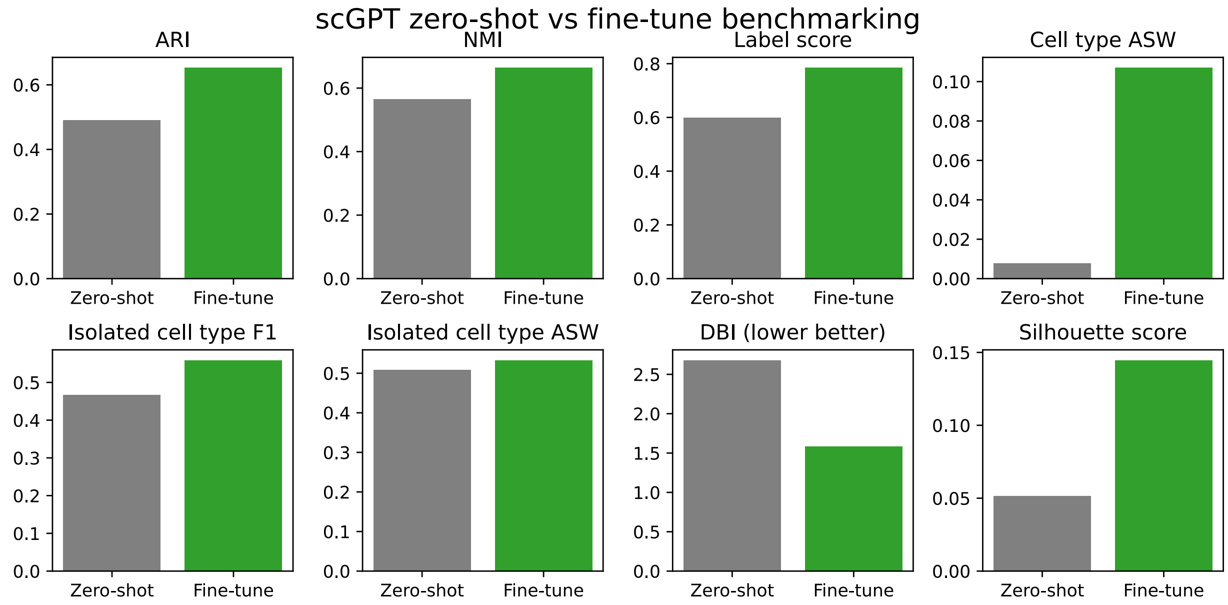

Supplementary Note Fig. 2 Embedding quality of IPF dataset generated by the zero-shot and fine-tune modes of the scGPT model. The fine-tune scGPT model outperforms the zero-shot setting.

Extending scGPT to unsupervised in-silico drug discovery tasks

By perturbing the gene expression of drug targets in cells and sending them to scGPT to generate embeddings of the perturbed cells, scGPT can implement the same unsupervised in-silico drug discovery function as UNAGI.

Supplementary Note 15

Evaluating the effectiveness of scGPT's drug perturbation ability using the snRNA-seq from the Precise Cut Lung Splice (PCLS) experiments

To predict gene expression changes after perturbation, scGPT needs to be trained in a supervised manner, like using perturb-seq datasets, which contain both control cells and cells after perturbation. Each perturbation experiment in the perturb-seq dataset usually targets only one or two genes. However, instead of targeting specific genes (e.g., knocking out one or two specific genes), our PCLS experiments directly treated cells with drugs in the ex-vivo treatments. In this scenario, the exact genes that were perturbed are not clear.

Training scGPT on perturbation tasks requires specifying the perturbed genes between two cell states (e.g., fibrosis cells and fibrosis cells with Nintedanib treatments). For this reason, we specified that all genes were perturbed in the ex-vivo treatments to fine-tune the scGPT models.

For our post-treatment gene expression prediction task, we perturbed only the top 10 differentially expressed genes (DEGs) between fibrosis cells and ex-vivo treatments to evaluate model effectiveness. We sent these fibrosis cells and the top 10 treatment DEGs of Nintedanib or Nifedipine to the fine-tuned scGPT to predict gene expression changes.

Supplementary Note 16

Details of third party packages and software used in this work

The custom software UNAGI, available at https://github.com/mcgilldinglab/UNAGI, is developed based on Python (version 3.9, available at https://www.python.org/). It also uses several public packages for data analysis, including pyro-ppl (version 1.8.6, available at https://pyro.ai/), scanpy (version 1.9.5, available at https://github.com/scverse/scanpy), PyTorch (version 2.0.0, available at https://github.com/pytorch/pytorch), numpy (version 1.24.1, available at https://github.com/numpy/numpy), scikit-learn (version 1.3.0, available at https://github.com/scikit-learn/scikit-learn), matplotlib (version 3.7.1, available at https://github.com/matplotlib/matplotlib), and pandas (version 2.1.0, available at https://github.com/pandas-dev/pandas), Cell Ranger (version 4.0.0, available at https://www.10xgenomics.com/support/software/cell-ranger/downloads), Cutadapt (version 4.1, available at https://cutadapt.readthedocs.io/en/stable/) and STAR (version 2.7.9a, available at https://github.com/alexdobin/STAR/tree/master) and Seurat (version 1.8.2, available at https://github.com/satijalab/seurat).

Reference

1. Yang, M.-S., Lai, C.-Y. & Lin, C.-Y. A robust EM clustering algorithm for Gaussian mixture models. *Pattern Recognition* **45**, 3950–3961 (2012).
2. Hu, P. *et al.* Dissecting Cell-Type Composition and Activity-Dependent Transcriptional State in Mammalian Brains by Massively Parallel Single-Nucleus RNA-Seq. *Molecular Cell* **68**, 1006-1015.e7 (2017).
3. Martin, M. Cutadapt removes adapter sequences from high-throughput sequencing reads. *EMBnet j.* **17**, 10 (2011).

4. Frankish, A. *et al.* GENCODE reference annotation for the human and mouse genomes. *Nucleic Acids Research* **47**, D766–D773 (2019).

5. Dobin, A. *et al.* STAR: ultrafast universal RNA-seq aligner. *Bioinformatics* **29**, 15–21 (2013).

6. Traag, V. A., Waltman, L. & Van Eck, N. J. From Louvain to Leiden: guaranteeing well-connected communities. *Sci Rep* **9**, 5233 (2019).

7. Ding, J., Hagood, J. S., Ambalavanan, N., Kaminski, N. & Bar-Joseph, Z. iDREM: Interactive visualization of dynamic regulatory networks. *PLoS Comput Biol* **14**, e1006019 (2018).

8. Moghieb, A. *et al.* Time-resolved proteome profiling of normal lung development. *Am J Physiol Lung Cell Mol Physiol* **315**, L11–L24 (2018).

9. Dylag, A. M. *et al.* New insights into the natural history of bronchopulmonary dysplasia from proteomics and multiplexed immunohistochemistry. *Am J Physiol Lung Cell Mol Physiol* **325**, L419–L433 (2023).

10. Woo, J. *et al.* Three-dimensional feature matching improves coverage for single-cell proteomics based on ion mobility filtering. *Cell Syst* **13**, 426-434.e4 (2022).

11. Zheng, G. X. Y. *et al.* Massively parallel digital transcriptional profiling of single cells. *Nat Commun* **8**, 14049 (2017).

12. Hao, Y. *et al.* Integrated analysis of multimodal single-cell data. *Cell* **184**, 3573-3587.e29 (2021).

13. Candès, E. J., Li, X., Ma, Y. & Wright, J. Robust principal component analysis? *J. ACM* **58**, 1–37 (2011).

14. Theodoris, C. V. *et al.* Transfer learning enables predictions in network biology. *Nature* **618**, 616–624 (2023).

15. Rosen, Y. *et al.* Universal Cell Embeddings: A Foundation Model for Cell Biology. Preprint at https://doi.org/10.1101/2023.11.28.568918 (2023).

16. Cui, H. *et al.* scGPT: toward building a foundation model for single-cell multi-omics using generative AI. *Nat Methods* (2024) doi:10.1038/s41592-024-02201-0.
