# Supplementary material for: A deep generative model for deciphering cellular dynamics and in silico drug discovery in complex diseases
Source: Nat Biomed Eng. 2025 Jun 20;9(12):2155–80. doi: 10.1038/s41551-025-01423-7 (PMC12705450; doi:10.1038/s41551-025-01423-7)
Supplement: Supplementary file 2 — Reporting Summary [file 41551_2025_1423_MOESM2_ESM.pdf]

nature portfolio

Corresponding author(s): Naftali Kaminski, Jun Ding

Last updated by author(s): Apr 18, 2025

Reporting Summary

Nature Portfolio wishes to improve the reproducibility of the work that we publish. This form provides structure for consistency and transparency in reporting. For further information on Nature Portfolio policies, see our Editorial Policies and the Editorial Policy Checklist.

Statistics

For all statistical analyses, confirm that the following items are present in the figure legend, table legend, main text, or Methods section.

n/a	Confirmed	
☐	☒	The exact sample size (*n*) for each experimental group/condition, given as a discrete number and unit of measurement
☐	☒	A statement on whether measurements were taken from distinct samples or whether the same sample was measured repeatedly
☐	☒	The statistical test(s) used AND whether they are one- or two-sided<br>*Only common tests should be described solely by name; describe more complex techniques in the Methods section.*
☒	☐	A description of all covariates tested
☐	☒	A description of any assumptions or corrections, such as tests of normality and adjustment for multiple comparisons
☐	☒	A full description of the statistical parameters including central tendency (e.g. means) or other basic estimates (e.g. regression coefficient) AND variation (e.g. standard deviation) or associated estimates of uncertainty (e.g. confidence intervals)
☐	☒	For null hypothesis testing, the test statistic (e.g. *F*, *t*, *r*) with confidence intervals, effect sizes, degrees of freedom and *P* value noted<br>*Give P values as exact values whenever suitable.*
☒	☐	For Bayesian analysis, information on the choice of priors and Markov chain Monte Carlo settings
☒	☐	For hierarchical and complex designs, identification of the appropriate level for tests and full reporting of outcomes
☐	☒	Estimates of effect sizes (e.g. Cohen's *d*, Pearson's *r*), indicating how they were calculated

Our web collection on statistics for biologists contains articles on many of the points above.

Software and code

Policy information about availability of computer code

Data collection	No software was used for data collection.
Data analysis	The custom software UNAGI, available at https://github.com/mcgilldinglab/UNAGI, is developed based on Python (version 3.9, available at https://www.python.org/). It also uses several public packages for data analysis, including pyro-ppl (version 1.8.6, available at https://pyro.ai/), scanpy (version 1.9.5, available at https://github.com/scverse/scanpy), PyTorch (version 2.0.0, available at https://github.com/pytorch/pytorch), numpy (version 1.24.1, available at https://github.com/numpy/numpy), scikit-learn (version 1.3.0, available at https://github.com/scikit-learn/scikit-learn), matplotlib (version 3.7.1, available at https://github.com/matplotlib/matplotlib), and pandas (version 2.1.0, available at https://github.com/pandas-dev/pandas), Cell Ranger (version 4.0.0, available at https://www.10xgenomics.com/support/software/cell-ranger/downloads), Cutadapt (version 4.1, available at https://cutadapt.readthedocs.io/en/stable/) and STAR (version 2.7.9a, available at https://github.com/alexdobin/STAR/tree/master) and Seurat (version 1.8.2, available at https://github.com/satijalab/seurat)

For manuscripts utilizing custom algorithms or software that are central to the research but not yet described in published literature, software must be made available to editors and reviewers. We strongly encourage code deposition in a community repository (e.g. GitHub). See the Nature Portfolio guidelines for submitting code & software for further information.

Data

Policy information about availability of data

All manuscripts must include a data availability statement. This statement should provide the following information, where applicable:

- Accession codes, unique identifiers, or web links for publicly available datasets
- A description of any restrictions on data availability
- For clinical datasets or third party data, please ensure that the statement adheres to our policy

IPF snRNA-seq (GSE286182) can be publicly accessible at https://www.ncbi.nlm.nih.gov/geo/query/acc.cgi?acc=GSE286182. The COVID-19 dataset (COVID-19 PBMC Ncl-Cambridge-UCL) is currently available from the COVID-19 Cell Atlas at https://covid19cellatlas.org/. The proteomics data are publicly available on MassIVE (Server:massive.ucsd.edu, User: MSV000093129, Password: Lung5172). The preprocessed PCLS data is available at our GitHub repository (https://github.com/mcgilldinglab/UNAGI). Hippie database can be publicly accessed at https://cbdm-01.zdv.uni-mainz.de/~mschaefer/hippie/download.php. STRINGDB is publicly available at https://string-db.org/. REACTOME can be accessed at https://reactome.org/, MatrisomeDB is available at https://matrisomedb.org/, and KEGG can be found at https://www.genome.jp/kegg/pathway.html. The Connectivity MAP (CMAP) database is publicly available on https://www.broadinstitute.org/connectivity-map-cmap.

Research involving human participants, their data, or biological material

Policy information about studies with human participants or human data. See also policy information about sex, gender (identity/presentation), and sexual orientation and race, ethnicity and racism.

Reporting on sex and gender	snRNA-seq data and proteomics were obtained from 9 patients with IPF, comprising 9 males, and 10 control subjects, comprising 9 males and one female. Similarly, PCLS snRNA-seq data were sourced from 6 males and 4 females. Sex- and gender-based analysis were not performed due to sample size limitations.
Reporting on race, ethnicity, or other socially relevant groupings	Biobanked samples are anonymized and we do not have any information regarding race, ethnicity, or other socially relevant groupings
Population characteristics	For the snRNA-seq IPF dataset, age of controls subjects ranging from 48 to 74 years, and from IPF subjects ranging from 51 to 64 years. All samples in the snRNA-seq IPF dataset were from Belgian donors and patients. For the PCLS data, age of subjects ranged from 24 to 68 years from American patients.
Recruitment	Biobanked tissue samples of patients with IPF undergoing lung transplantation as well as donor lungs not suitable for transplantation as controls were obtained. For the PLCS experiment, Donor lung samples, unsuitable for lung transplantation were obtained from the Center for Organ Recovery and Education (CORE) at the University of Pittsburgh.
Ethics oversight	Biobanking was approved by the local medical ethics committee of the KU Leuven University Hospital, Belgium (ML6385). A secondary approval (# 2000025427) at Yale Institutional Review Board was obtained. PCLS biobanking was approved by the University of Pittsburgh (IRB PRO14010265)

Note that full information on the approval of the study protocol must also be provided in the manuscript.

Field-specific reporting

Please select the one below that is the best fit for your research. If you are not sure, read the appropriate sections before making your selection.

☒ Life sciences ☐ Behavioural & social sciences ☐ Ecological, evolutionary & environmental sciences

For a reference copy of the document with all sections, see nature.com/documents/nr-reporting-summary-flat.pdf

Life sciences study design

All studies must disclose on these points even when the disclosure is negative.

Sample size	For the IPF snRNA-seq dataset, we analyzed 231,477 cells from 10 healthy donors and 9 IPF patients; The proteomics data was obtained from the same samples as those used for the IPF snRNA-seq dataset. In the case of the PCLS snRNA-seq dataset, it included 23,927 fibroblast cells from a total of 10 donors, comprising 6 male and 4 female donors. and for the COVID-19 PBMC Ncl-Cambridge-UCL dataset, we analyzed a total of 246,948 cells from 47 patients, comprising 26 male and 21 female donors.
Data exclusions	In the IPF snRNA-seq dataset, low-quality cells were removed as described in the 'Dataset description and preprocessing' section of the Method. For the PCLS snRNA-seq dataset, low-quality cells were excluded according to the procedures detailed in the 'Precision-cut lung slice (PCLS) experiments' section of the Method. Additionally, only fibroblast cells from the PCLS dataset were utilized. Regarding the COVID-19 dataset, the patient age range was limited to 50-70 years to minimize the sample size. Any cells expressing fewer than 300 genes and the proportion of total counts for a cell are mitochondrial >4% were considered low quality and subsequently removed.
Replication	We confirmed all the computational results were reproducible by running our UNAGI framework (https://github.com/mcgilldinglab/UNAGI)

Replication	multiple times using random initializations.
Randomization	To identify dynamic markers, we generated a random background foldchange distribution by permuting gene expressions multiple times (N=1000). For evaluating pathway and drug in-silico perturbations, we created a background perturbation score distribution by randomly sampling gene sets for in-silico perturbation. Furthermore, in our benchmarking and ablation experiments, multiple random seeds were used to train UNAGI (N=15).
Blinding	In the snRNA-seq IPF and snRNA-seq PCLS datasets, patient samples have been de-identified to ensure privacy and confidentiality. For detailed information on the COVID-19 PBMC Ncl-Cambridge-UCL dataset, please refer to the COVID-19 Cell Atlas website (https://covid19cellatlas.org)

Reporting for specific materials, systems and methods

We require information from authors about some types of materials, experimental systems and methods used in many studies. Here, indicate whether each material, system or method listed is relevant to your study. If you are not sure if a list item applies to your research, read the appropriate section before selecting a response.

Materials & experimental systems

n/a	Involved in the study	
☒	☐	Antibodies
☒	☐	Eukaryotic cell lines
☒	☐	Palaeontology and archaeology
☒	☐	Animals and other organisms
☒	☐	Clinical data
☒	☐	Dual use research of concern
☒	☐	Plants

Methods

n/a	Involved in the study	
☒	☐	ChIP-seq
☒	☐	Flow cytometry
☒	☐	MRI-based neuroimaging

Plants

Seed stocks	*Report on the source of all seed stocks or other plant material used. If applicable, state the seed stock centre and catalogue number. If plant specimens were collected from the field, describe the collection location, date and sampling procedures.*
Novel plant genotypes	*Describe the methods by which all novel plant genotypes were produced. This includes those generated by transgenic approaches, gene editing, chemical/radiation-based mutagenesis and hybridization. For transgenic lines, describe the transformation method, the number of independent lines analyzed and the generation upon which experiments were performed. For gene-edited lines, describe the editor used, the endogenous sequence targeted for editing, the targeting guide RNA sequence (if applicable) and how the editor was applied.*
Authentication	*Describe any authentication procedures for each seed stock used or novel genotype generated. Describe any experiments used to assess the effect of a mutation and, where applicable, how potential secondary effects (e.g. second site T-DNA insertions, mosiacism, off-target gene editing) were examined.*
